# Gene discovery and virus-induced gene silencing reveal branched pathways to major classes of bioactive diterpenoids in *Euphorbia peplus*

**DOI:** 10.1073/pnas.2203890119

**Published:** 2022-05-18

**Authors:** Tomasz Czechowski, Edith Forestier, Sandesh H. Swamidatta, Alison D. Gilday, Amy Cording, Tony R. Larson, David Harvey, Yi Li, Zhesi He, Andrew J. King, Geoffrey D. Brown, Ian A. Graham

**Affiliations:** ^a^Department of Biology, Centre for Novel Agricultural Products, University of York, York YO10 5DD, United Kingdom;; ^b^Department of Chemistry, University of Reading, Reading RG6 6AD, United Kingdom

**Keywords:** terpenoids, oxidation, cytochrome P450s, short-chain dehydrogenases/reductases, virus-induced gene silencing

## Abstract

Most macro- and polycyclic Euphorbiaceae diterpenoids derive from the common C_20_ precursor casbene. While the biosynthetic pathway from casbene to the lathyrane jolkinol C is characterized, pathways to other more complex classes of bioactive diterpenoids remain to be elucidated. A metabolomics-guided transcriptomic approach and a genomics approach that led to the discovery of two casbene-derived diterpenoid gene clusters yielded a total of 68 candidate genes that were transiently expressed in *Nicotiana benthamiana* for activity toward jolkinol C and other lathyranes. We report two short-chain dehydrogenases/reductases (SDRs), identified by RNA sequencing to be highly expressed in *Euphorbia peplus* latex. One of these, *EpSDR-5*, is a C3-ketoreductase, converting jolkinol C to the lathyrane jolkinol E. Gene function of *EpSDR-5* was further confirmed by heterologous expression in *Saccharomyces cerevisiae*. To investigate the *in vivo* role of *EpSDR-5*, we established virus-induced gene silencing (VIGS) in *E. peplus*, resulting in a significant reduction in jatrophanes and a corresponding increase in ingenanes. VIGS of *Casbene Synthase* results in a major reduction in both jatrophanes and ingenanes, the two most abundant classes of *E. peplus* diterpenoids. VIGS of *CYP71D365* had a similar effect, consistent with the previously determined role of this gene in the pathway to jolkinol C. These results point to jolkinol C being a branch point intermediate in the pathways to ingenanes and jatrophanes with EpSDR-5 responsible for the first step from jolkinol C to jatrophane production.

## Significance

*Euphorbia peplus*, a member of the *Euphorbia* genus, is rich in jatrophane and ingenane diterpenoids. Using a metabolomics-guided transcriptomic approach to gene candidate identification, we have discovered a short-chain dehydrogenase gene involved in the production of the lathyrane jolkinol E. We have developed a virus-induced gene-silencing method in *E. peplus* that has allowed us to demonstrate the direct relationship between casbene and polycyclic diterpenoids and that jolkinol C acts as a key branch point intermediate in the production of ingenanes and jatrophanes. This work contributes both knowledge and tools for engineering production of bioactive diterpenoids in heterologous host systems, thus enabling their further evaluation and development.

The Euphorbiaceae family of flowering plants produces a diverse range of 20-carbon casbene-derived diterpenoids (1) including the lathyranes, which are inhibitors of ATP-binding cassette transporters. These transporters are responsible for the efflux of chemotherapy drugs in multidrug-resistant cancers (2) as well as an efflux of drugs for the treatment of fungal (3) and protozoal (4) pathogens. The lathyranes are also believed to serve as precursors of many other bioactive diterpenoids including ingenol mebutate (IM), a licensed pharmaceutical used for the treatment of actinic keratosis, which is extracted from the aerial parts of *Euphorbia peplus* (5); tigilanol tiglate, an antitumor compound that is extracted from seeds of *Fontainea picrosperma* (6); and resiniferatoxin, a capsaicin analog currently in phase 1b clinical trials for the treatment of cancer-related intractable pain, which can be extracted from the latex of *Euphorbia resinifera*, *Euphorbia poissonii*, and *Euphorbia fischeriana* (7). Several casbene-derived phorbol esters exhibit in vitro antiviral activities including prostratin, a lead compound for the treatment of latent HIV infections (8), and others that exhibit activity against chikungunya virus and HIV (9). With over 500 isolated structures, jatrophanes make up a particularly large class with members exhibiting various biological activities including a reversal of multidrug resistance in cancer cell lines overexpressing P-glycoprotein (10, 11). A low abundance of many of these compounds in their natural hosts (12, 13) together with challenging chemical synthesis due to high structural complexity (14–16) means that alternative production platforms are needed if the full potential for industrial applications is to be realized. Metabolic engineering of microbial, algal, or plant-based production platforms offers such an alternative once the gene toolkit becomes available.

Jolkinol C [1] biosynthesis has been determined in several Euphorbiaceae species including *Jatropha curcas* (17), *Euphorbia lathyris* (18), and *E. peplus* (18). Two P450-mediated oxidations introduce keto- and hydroxy-groups at positions 5-, 6-, and 9-, of casbene, thereby enabling a spontaneous intramolecular aldol reaction that forms a new carbon–carbon bond between the 6- and 10-positions. The introduction into *Saccharomyces cerevisiae* of *J. curcas* casbene synthase (*JcCAS*) and two cytochrome P450 oxidases (*CYP726A20* and *CYP71D495*) along with an alcohol dehydrogenase (*JcADH1*) results in jolkinol C [1] production at 800 mg/L culture medium (19). Casbene synthesis has also recently been achieved in the green algae *Chlamydomonas reinhardtii* at a 19-mg/L culture (20). Lathyranes, such as jolkinol C [1], have been proposed as advanced precursors for the biosynthesis of several classes of diterpenoids including ingenanes and tiglanes (21). Recent work has identified several candidate acyl-coenzyme A ligases potentially involved in angeloylation of ingenol to IM (22). However, the biosynthetic pathways leading from casbene to jatrophanes, ingenanes, and other polycyclic diterpenoids remain hypothetical. and the present study addresses this knowledge gap.

## Results and Discussion

### A Combined Metabolomics and Transcriptomics Approach to Diterpenoid-Related Gene Discovery in *E. peplus*.

**Fig. 1.**
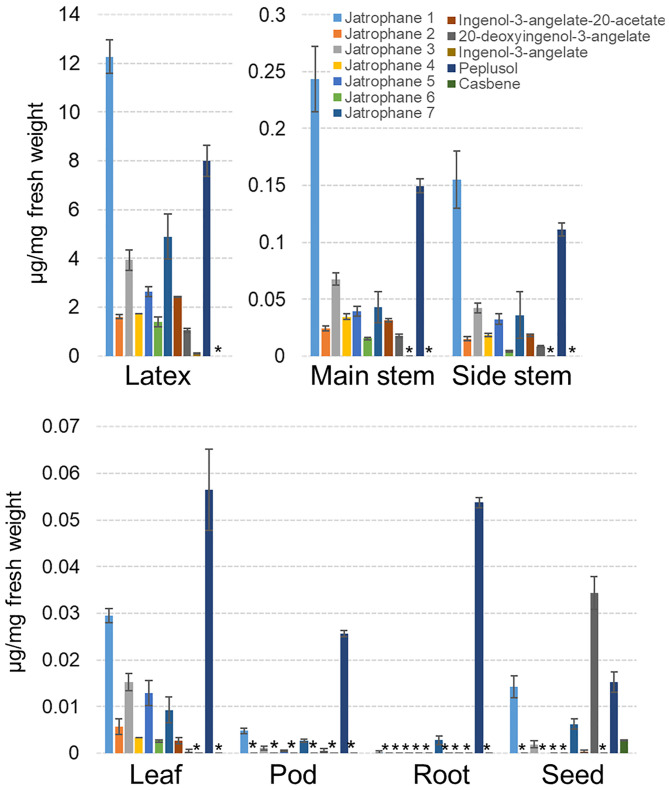
Liquid chromatography (LC)-MS and gas chromatography (GC)-MS quantitation of selected terpenoids from *E. peplus* tissues. Plant material from 8-wk-old plants was extracted and analyzed by LC-MS and GC-MS as described in the *SI Appendix*, *SI Materials and Methods*. Error bars represent SE from four biological replicates. Asterisks indicate where the compound was not detectable by LC-MS and GC-MS, with compound order in bar charts consistent with the color key. Note that peplusol represents the total concentration of the two peaks identified for this compound (Table S1).

Metabolomic and NMR analysis were performed to establish the diterpenoid composition of various tissues of *E. peplus* and to guide subsequent RNA sequencing (RNAseq) analysis aimed at the discovery of candidate genes involved in the biosynthesis of IM (Fig. 1 and *SI Appendix*, Figs. S1 and S2 *A* and *B* and Table S1). Three of the ingenanes characterized by NMR, namely, ingenol-3-angelate (IM), ingenol-3-angelate-20-acetate, and 20-deoxyingenol-3-angelate, have been described previously (23, 24). These ingenanes were most abundant in latex and latex-containing tissues such as main stems and side stems and to a lesser extent in leaves and pods but were absent from latex-free roots (Fig. 1 and Table S1). The 20-deoxyingenol-3-angelate accumulates in dry seeds (Fig. 1 and Table S1). IM was only detectable in latex as a very minor constituent (0.11 μg/mg of fresh tissue), while the concentration of its 20-acetylated derivative (ingenol-3-angelate-20-acetate), was 22-fold higher (Fig. 1 and Table S1) and detectable in other latex-containing tissues and dry seeds. In agreement with previously published data (23, 24), we have also found a number of heavily modified jatrophanes (Jatrophanes 1 to 4), carrying between three to six acyl groups in addition to other functional groups, such as a benzoyl group at the 3-position, an isobutanoyl group at the 7-position, and a nicotinic acid ester at the 9-position (*SI Appendix*, Figs. S1 and S2*A*). We have identified three additional jatrophanes (Jatrophanes 5 to 7) with mass-to-charge ratio (*m/z*) spectra that match previously published jatrophanes from *E. peplus* latex (24) (*SI Appendix*, Fig. S2*B*). All seven jatrophanes were most abundant in latex and latex-containing main stem, side stem, and leaf tissues and, except for Jatrophane 7, were absent from latex-free roots (Fig. 1 and Table S1). Peplusol is a triterpene alcohol with strong antifungal activities (24) that was previously described as being responsible for the physical properties of *E. peplus* latex (25), but we have now identified it to be also present in pods and latex-free roots (Fig. 1 and Table S1). It is noteworthy that we only found trace amounts of casbene in seed tissue (Fig. 1) and lathyranes including jolkinol C were not detected in any of the tissues analyzed, suggesting that the proposed intermediates for IM biosynthesis are rapidly converted to pathway end-products.

To identify candidate genes associated with the biosynthesis of specific metabolites, we performed RNAseq analysis on messenger RNA extracted from both latex-containing tissues (main stems, leaves and pods) and latex-free tissues (roots), as well as isolated latex (*SI Appendix*, *SI Materials and Methods* and Table S4). RNAseq data were de novo assembled into 201,887 contigs with an average contig length of 2 kb (1.3-kb median). Further analysis revealed that 1,453 contigs were expressed at a significantly higher level in latex and/or stem tissue (out of a total of 106,578 expressed contigs). Functional annotations were assigned for the selected 1,453 contigs using the SwissProt database. The number of candidate genes was reduced to 46 by focusing on those predicted on the basis of homology to encode enzymes expected to be involved in ingenol synthesis and modification, including cytochrome P450 monooxygenases, dioxygenases, alkenal reductases, hydroxylases, dehydratases, dehydrogenases, and epoxidases (*SI Appendix*, Fig. S3).

### Discovery of Two Gene Clusters Involved in Diterpenoid Metabolism in *E. peplus*.

Our previous work indicated that the *E. peplus CAsbene Synthase* (*EpCAS*) and *casbene-5-oxidase* (*EpCYP726A19*) genes are physically linked in the *E. peplus* genome (26). Further to this, Luo et al. (18) also reported the characterization of jolkinol C biosynthetic genes from *E. peplus*, in which *CYP726A4* appeared to be a casbene-5-hydroxylase, and *CYP71D365* was a casbene-9-oxidase (18). We constructed a 2.36-Gbp bacterial artificial chromosome (BAC) library from *E. peplus* genomic DNA (equivalent to 7× genome coverage) (27) and screened this initially using primers for *CYP726A4* and *EpCAS*. Subsequent rounds of screening, which were performed using primers designed against terminal regions of the BAC ends, led to the identification of two gene clusters containing known genes for casbene synthesis and casbene modification, surrounded by several other genes that could potentially be involved in diterpenoid biosynthesis (*SI Appendix*, Fig. S4). Gene cluster 1 is 447 Kbp long and contains a casbene synthase sequence next to *CYP726A19*, confirming our previous results (26). These genes are in close proximity to the previously published *EpADH1* sequence (18) (*SI Appendix*, Fig. S4), which is adjacent to *EpADH2* with 81% identity at the nucleotide level. This gene cluster, composed of 53 genes and 1 retrotransposon, also contains 14 P450 oxidase homologs, mostly from the CYP726A clan, as well as gene homologs of other enzymes potentially involved in diterpenoid biosynthesis. These include dioxygenases, an oxido-reductase, a ketoreductase, an alkenal-reductase, and a crotonase (*SI Appendix*, Fig. S4). Three of the gene cluster 1 P450 oxidases (*CYP726A3*, *CYP726A5*, and *CYP726A6*) have been previously reported (18). Gene cluster 2 contains functionally characterized *CYP726A4* and *CYP71D365* (18) surrounded by nine genes potentially involved in diterpenoid oxidation and other modifications, including seven P450 oxidases from the CYP71D and CYP726A clans, a crotonase, and a carboxylesterase (*SI Appendix*, Fig. S4).

**Fig. 2.**
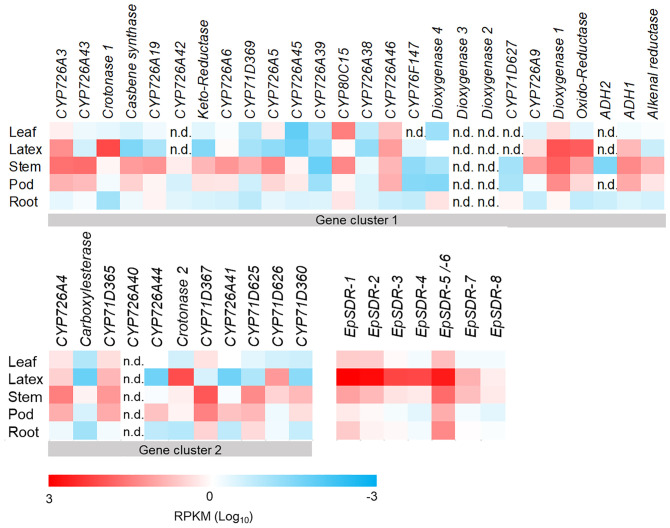
Expression levels of candidate genes from two casbene-derived diterpenoid gene clusters and a nonclustered gene family of SDRs expressed predominantly in *E. peplus* latex. Genes from the two *E. peplus* casbene-derived diterpenoid gene clusters are ordered according to relative genomic location. RPKM, reads per kilobase of transcript, per million mapped reads; n.d., no reads detected.

RNAseq-based gene expression profiling of the 5 functionally characterized genes from the 2 gene clusters together with 32 other candidate diterpenoid genes revealed that the *Casbene synthase* expression pattern broadly overlaps with that of the 3 known casbene oxidases as well as *EpADH1*, with high expression levels in roots and stems and intermediate levels in developing pods (Fig. 2) Interestingly, *Casbene synthase* and *CYP726A19* expression were extremely low compared to *CYP726A4*, *CYP71D365*, and *ADH1* in diterpenoid-rich latex tissue (Fig. 2). The coordinate expression of genes associated with these two clusters provides further evidence that they are functionally related, as is the case with other plant gene clusters (28). The majority of cluster 1 genes are expressed highly in stems and at lower levels in roots, but their expression pattern varies among the other tissues (Fig. 2). *Crotonase-1*, *Dioxygenase-1*, and *Oxido-Reductase* from gene cluster 1 are highly expressed in latex, whereas other genes, including *EpADH2*, *Dioxygenase 4*, and *CYP71D627*, are expressed preferentially in roots (Fig. 2). *CYP80C15* is the only gene highly expressed in leaves. The majority of cluster 2 genes including the functionally characterized *CYP726A4* and *CYP71D365* are expressed most highly in stem and pods (Fig. 2). *Crotonase 2* and *CYP71D626* are the only two genes expressed highly in latex (Fig. 2). A few genes from gene clusters 1 and 2, such as *Dioxygenase-2* and *Dioxygenase-3*, and *CYP726A40*, were not expressed in any of the tissues analyzed (Fig. 2).

### Identification of *E. peplus* Gene Products That Utilize Jolkinol C [1]/*epi-*Jolkinol C [2] as Reaction Substrates.

**Fig. 3.**
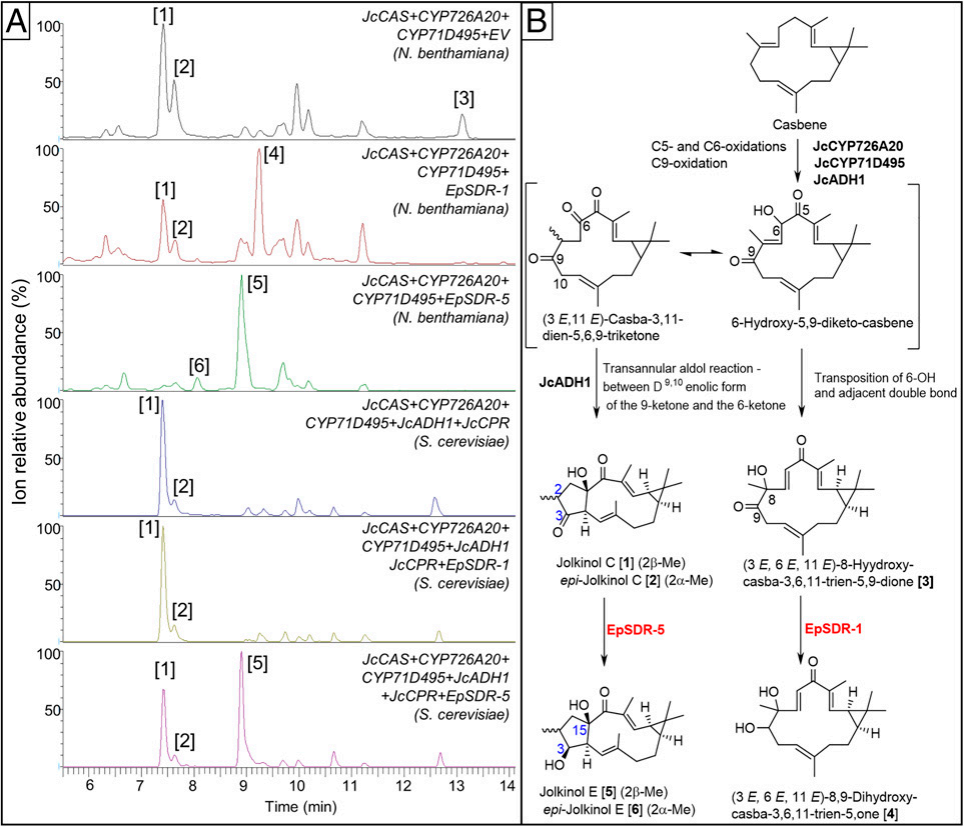
Discovery of diterpenoid-modifying activities among *E. peplus* short-chain dehydrogenases. (*A*) Four-week-old *N. benthamiana* plants infiltrated with MEP-pathway genes (*AtDXS*, *AtHDR*, and *AtGGPPs*), *JcCAS*, casbene 5,6-oxidase (*CYP726A20*), casbene 9-oxidase (*CYP71D495*), and either: empty vector (EV) or *E. peplus* SHORT-CHAIN DEHYDROGENASE 1 (*EpSDR-1*, GenBank accession MW594428) *or EpSDR-5* (GenBank accession MW594431). Infiltrated leaves were extracted and analyzed by LC-MS (*SI Appendix*, *SI Materials and Methods*). Raw chromatograms for *m/z* 317 to 320 are shown. Peaks are numbered according to the structures shown in *B*. (*B*) Enzymatic steps catalyzed by *J. curcas* casbene oxidases and *E. peplus* SDRs are shown. Structures [4], [5] and [6] were determined by NMR (*SI Appendix*, *SI Materials and Methods*). Compound numbering corresponds with the peak numbering in *A*. Lathyrane ring numbering is shown in blue.

A total of 28 *E. peplus* genes from the 2 gene clusters and 40 candidate genes identified by RNAseq were cloned into pEAQ-HT vectors and transiently expressed in *N. benthamiana* to test for activity using jolkinol C [1] and *epi-*jolkinol C [2] as substrates. In all cases, the coinfiltration mixture contained either a candidate gene in the pEAQ-HT vector or an empty vector control together with three isoprenoid precursor-supply genes from *Arabidopsis thaliana*, namely, *AtDXS*, *AtHDR*, and *AtGGPPS*, in combination with three genes from the previously published jolkinol C biosynthetic gene cluster from *J. curcas* (17), namely, *JcCAS*, *CYP726A20*, and *CYP71D495* (*SI Appendix*, *SI Materials and Methods*). This particular combination of six genes results in the production of [1] when expressed transiently in *N. benthamiana* (17, 29). Of the 68 genes tested, only 2 members of a 7-gene family of short-chain dehydrogenases/reductases (SDRs), identified by RNAseq to be expressed highly in latex and/or stems (Fig. 2) were found to have activity when transiently expressed in *N. benthamiana* with the gene combination that produces [1] (Fig. 3). *EpSDR-1* produced a peak eluting at 9.4 min (peak 4, Fig. 3*A*), with an [M+H]+ of *m/z* 319, corresponding to the molecular formula C_20_H_30_O_3_ (*SI Appendix*, Fig. S4). A peak with a similar retention time and an identical molecular ion had previously been identified as 6,9-dihydroxy-5-ketocasbene (29), which suggested that the product of *EpSDR-1* might be structurally related. Production of this peak was accompanied by a slight decrease in the accumulation of [1] and [2] (peaks 1 and 2, Fig. 3*A*) when compared to the control. Expression of *EpSDR-1* also led to a very strong decrease in the level of (3E, 6E, 11E)-8-hydroxy-casba-3,6,11-trien-5,9-dione (peak 3, Fig. 3*A*).

Infiltration of *EpSDR-5* produced two new peaks at 9 min (peak 5, Fig. 3*A*) and 8 min (peak 6, Fig. 3*A*) with levels of [1] and [2] reducing much more than for the *EpSDR-1* infiltrations (Fig. 3*B*). Mass spectrometry data once again showed an [M+H]+ molecular ion at *m/z* 319 for the molecular formula C_20_H_30_O_3_, consistent with the identification of peaks 5 and 6 as reduced forms of jolkinol C [1] and *epi-*jolkinol C [2] (*SI Appendix*, Fig. S5). The structures of the metabolites responsible for peaks 4, 5, and 6 were all elucidated by NMR spectroscopy, following scaled-up production and extraction from *N. benthamiana* leaves (*SI Appendix*, *SI Materials and Methods*). One-dimensional and two-dimensional NMR analyses revealed the compounds forming peaks 4, 5, and 6 in Fig. 3*A* to be (3E, 6E, 11E)-8,9-dihydroxy-casba-3,6,11-trien-5-one [4]; the 3β-hydroxy reduction product of jolkinol C, which we have named as jolkinol E [5]; and its 2-epimer, *epi-*jolkinol E [6], respectively.

*Nicotiana* species naturally produce oxygenated macrocyclic diterpenoids (30–32), and to ensure endogenous host enzyme activities were not confounding the functional characterization of candidate genes, we also tested the activity of the of *EpSDR-1* and *EpSDR-5* gene products in *S. cerevisiae*. We engineered jolkinol C production in a GGPP precursor-optimized *S. cerevisiae* strain following a previously published strategy (19). This involved chromosomal integration of codon-optimized versions of *Casbene Synthase* (N and C terminus tagged), *casbene-5,6-oxidase* (*CYP726A20*), *casbene-9-oxidase* (*CYP71D495*), *cytochrome P450 reductase* (*JcCPR*), and *alcohol dehydrogenase* (*JcADH1*) from *J. curcas* (*SI Appendix*, *SI Materials and Methods*). Expression of codon-optimized *EpSDR-1* in the jolkinol-C–producing strain did not yield any of the (3E, 6E, 11E)-8,9-dihydroxy-casba-3,6,11-trien-5-one [4] (Fig. 3*A*). This may be explained by the fact that the jolkinol-C–producing *S. cerevisiae* strain does not accumulate (3E, 6E, 11E)-8-hydroxy-casba-3,6,11-trien-5,9-dione [3], the proposed substrate of EpSDR-1. This compound was previously described as a dead-end metabolite from the combined activity of CYP726A20 and CYP71D495 on casbene in the absence of the concomitant transannular cyclization to the lathyrane skeleton (17) (Fig. 3*B*). These results from *S. cerevisiae* now suggest that the production of [3] by a 6-OH to 8-OH transposition (Fig. 3*B*) in the *N. benthamiana* heterologous host could be due to endogenous enzymatic activities that are absent in the *S. cerevisiae* host rather than the combined activity of CYP726A20 and CYP71D495 as previously proposed.

Expression of codon-optimized *EpSDR-5* in the jolkinol-C–producing *S. cerevisiae* strain did yield jolkinol E, further confirming the functional characterization of this gene. It is noteworthy that EpSDR-5 appears to accept both jolkinol C [1] and *epi*-jolkinol C [2] as substrates when reducing the 3-keto group to form the two stereoisomers, namely, jolkinol E [5] and *epi*-jolkinol E [6], in *N. benthamiana*. However, we were unable to detect *epi*-jolkinol E [6] while heterologously expressing *EpSDR-5* in *S. cerevisiae* (Fig. 3*A*). Although engineered *S. cerevisiae* strains are able to produce *epi*-jolkinol C, levels are considerably lower than in *N. benthamiana* (Fig. 3*A*), which may cause *epi*-jolkinol E production to fall below detection limits. Alternatively, *EpSDR-5* function may be different in *S. cerevisiae* compared to the plant host with *epi*-jolkinol C not acting as the substrate. In any case, it is likely that the *epi*-jolkinol C conversion represents a promiscuous activity not relevant in the native host, as the vast majority of presumably lathyrane (jolkinol C)–derived jatrophanes, ingenanes, and tiglanes have the C2β methyl group configuration with the C2-epimer methyl configuration not involved.

Protein sequence alignments of the seven members of the *E. peplus* latex-specific *SDRs* showed two distinct subgroups, namely, one homologous to *EpSDR-1* and the other to *EpSDR-5* (*SI Appendix*, Fig. S6). Phylogenetic analysis of complementary DNA (cDNA)–deduced amino acid sequences showed that *EpSDR-1*, *EpSDR-2*, *EpSDR-4*, and *EpSDR-8* belong to clade 1 of the SDR7C family of plant SDRs (*SI Appendix*, Fig. S7*A*) (33). This clade includes functionally characterized short-chain dehydrogenases from *A. thaliana* and *Pisum sativum* involved in the chloroplast protein import translocon that operates at the chloroplast inner envelope membrane (34). *EpSDR-5*, *EpSDR-6*, and *EpSDR-7* belong to clade 1 of the SDR114C family (33) (*SI Appendix*, Fig. S7*B*). This clade includes a number of functionally characterized genes involved in alkaloid and monoterpene metabolism, including salutaridine reductases from *Papaver bracteatum* and *Papaver somniferum* (35), (+)-neomenthol dehydrogenases from *A. thaliana* and *Capsicum annuum* (36), and (−)-isopiperitenone reductase from *Mentha* × *piperita* (33, 37).

EpSDR-1 and EpSDR-5 protein sequences also share very low homology (<20%) with two previously characterized Euphorbiaceae alcohol dehydrogenases, namely, EpADH1 from *E. peplus* and ElADH1 from *E. lathyris*, and JcADH1 from *J. curcas* (*SI Appendix*, Fig. S6). These three ADHs belong to the SDR110C family and have a role in jolkinol C biosynthesis in heterologous hosts (18, 19). That work demonstrated that the addition of either *JcADH1* or *ElADH1* to *N. benthamiana* or *S. cerevisiae* resulted in dehydrogenation of the hydroxyl groups introduced by cytochrome P450s from *E. peplus* and *E. lathyris* at position C5, C6, and C9 of the casbene ring, resulting in a rearrangement and cyclization via an intramolecular aldol reaction and overall increase in jolkinol C production (18, 19). To investigate if similar dehydrogenation had any effect on products downstream of jolkinol C, we included *JcADH1* from the *J. curcas* jolkinol C biosynthetic gene cluster (17) in *N. benthamiana* transient expression experiments but found no effect on the biosynthesis of (3E, 6E, 11E)-8,9-dihydroxy-casba-3,6,11-trien-5-one [4], jolkinol E [5], or *epi*-jolkinol E [6] (*SI Appendix*, Fig. S8).

Although several derivatives of jolkinol E have been detected in the Euphorbiaceae (38), jolkinol E itself has not previously been described as a natural product. Furthermore, the vast majority of the jatrophanes and ingenanes cited in the literature have undergone similar reduction to a 3β-hydroxy group (1, 10) and the introduction of a 3-hydroxy functional group renders the ingenol backbone amenable to Angeloylation in the biosynthesis of IM. In contrast, the majority of tiglanes contain a 3-keto functional group (1). If we assume tiglanes are also derived from jolkinol C, this may indicate they are derived from a distinct biosynthetic route to that involved in the production of jatrophanes and ingenanes.

### In Planta Confirmation of Diterpene Biosynthetic Activities of *E. peplus* Genes Using VIGS.

**Fig. 4.**
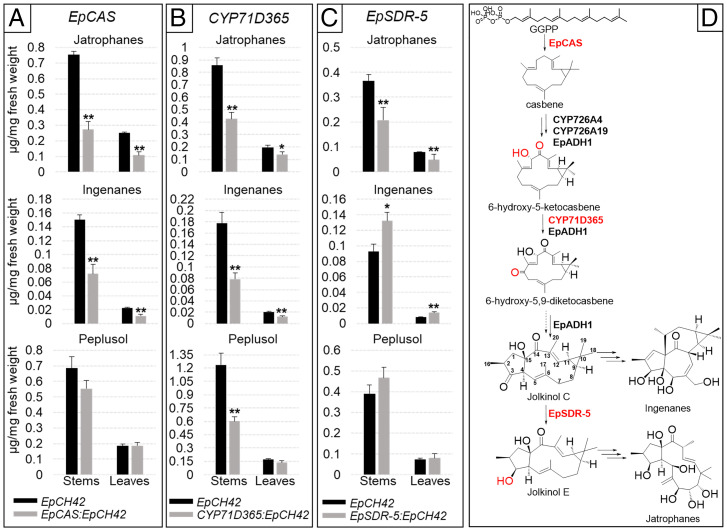
VIGS analysis of diterpenoid biosynthetic genes in *E. peplus.* Metabolite levels in VIGS material were measured for stem (black bars) and leaves (light-gray bars) in VIGS marker-only (*EpCH42*) and marker plus diterpenoid-pathway–silenced genes *EpCAS:EpCH42* (*A*), *CYP71D365:EpCH42* (*B*), and *EpSDR-5:EpCH42* (*C*). Jatrophanes represent the sum of seven jatrophanes annotated in Tables S2–S4, ingenanes represent the sum of three ingenanes annotated in Tables S2–S4, and peplusol represents total concentration of the two peaks identified for this compound (Tables S2–S4). Error bars, SEM (*n* = 5). Statistically significant (t test) changes between control (*EpCH42*) and diterpenoid-pathway–silenced genes are indicated by asterisks separately for each tissue (*P < 0.05; **P < 0.01). (D) Proposed model for jatrophane and ingenane biosynthetic pathways in *E. peplus*, based on heterologous expression data (17) and VIGS. Enzymes subjected to VIGS and corresponding reactions are highlighted in red. Solid arrows represent enzymatic reactions; dashed arrows represent proposed nonenzymatic reactions. Note that ingenane and jatrophane structures are presented as consensus molecules that will be subject to further modifications in planta.

We next established virus-induced gene silencing (VIGS) (39) in *E. peplus* seedlings to directly investigate the in planta role of selected diterpenoid-related genes. This was facilitated by the identification of an *E. peplus* gene encoding a subunit of Mg chelatase (*EpCH42*; *SI Appendix*, Fig. S9), the homolog of which was shown to act as a visual marker for VIGS in *J. curcas* (40, 41). The two genes share 82% nucleotide identity and *EpCH42* is expressed highly in leaves and stems (*SI Appendix*, Fig. S9 *A* and *B*). First, chlorosis in cotyledons was seen 5 to 6 d after infiltration of 9-d-old seedlings, progressing to leaves and eventually stems (*SI Appendix*, Fig. S10). *Agrobacterium tumefaciens*–based infiltration of pTRV2 VIGS constructs containing *EpCH42* either alone or combined with *EpCAS*, *Casbene-9-oxidase* (*CYP71D365*), or *jolkinol C 3-ketoreductase* (*EpSDR-5*) into 9-d-old *E. peplus* seedlings was performed, and chlorotic parts of stems and leaves were harvested around 40 d postinfiltration as described in *SI Appendi*x, *SI Materials and Methods*. Metabolite analysis revealed that VIGS of *EpCAS* significantly (over twofold) decreased the level of jatrophanes and ingenanes in stems and leaves (Fig. 4*A* and Table S2). Transcript levels of *EpCAS* were also significantly reduced in stems with a threefold to fourfold reduction compared with *EpCH42*-infiltrated controls (*SI Appendix*, Fig. S11*A*). These in planta results are consistent with the long-held view that jatrophanes and ingenanes are casbene derived in the Euphorbiaceae (10, 21). Levels of an abundant triterpene alcohol, peplusol, were not significantly affected in leaves or stems of *EpCAS*-silenced plants (Fig. 4*A* and Table S2), consistent with this natural compound being squalene and not casbene derived.

VIGS of *CYP71D365*, a key gene in the biosynthesis of jolkinol C resulted in a threefold to fourfold reduction in transcript levels (*SI Appendix*, Fig. S11 *C* and *D*) and over twofold reduction in both jatrophanes and ingenanes in stems with less pronounced effects in leaves (Fig. 4*B* and Table S3). These results are consistent with the lathyrane jolkinol C being an intermediate in the biosynthetic pathway to both ingenanes and jatrophanes. Biosynthesis of jatrophanes has been postulated to occur either directly from casbene, by the opening of the cyclopropane ring followed by formation of the five-membered ring between C-6 and C-10, or derived from lathyranes by opening of the cyclopropane ring (10). The results presented in Fig. 4 are consistent with the latter route to jatrophanes in *E. peplus.* Interestingly, the level of peplusol is also significantly (twofold) reduced by VIGS of *CYP71D365* in stems (Fig. 4*B* and Table S3). The basis for this reduction is not known, but it could be due to either off-target silencing effects on other P450 oxidases involved in the biosynthesis of peplusol or by direct involvement of *CYP71D365* in this process. We have observed a strong accumulation of both 5-ketocasbene and 6-hydroxy-5-ketocasbene in the *CYP71D365*-silenced stem and leaf tissues, and both of these compounds are on the margins of detectability in control (*EpCH42*-silenced) plants (Table S3). These in planta results are consistent with the results of a transient expression of *CYP71D365* in *N. benthamiana* that concluded that both 5-ketocasbene and 6-hydroxy-5-ketocasbene are substrates of the corresponding enzyme (18). The VIGS in planta results also indicate that C5- and C6-oxidation of casbene occur before C9-oxidation in the pathway to jolkinol C.

*CYP71D365* (*casbene-9-oxidase*) is present on *E. peplus* casbene-derived diterpenoids gene cluster 2, and a phylogenetic analysis revealed that it forms a distinct cluster in clade 71D with five other homologs encoded across both gene clusters (*SI Appendix*, Fig. S12). Very stringent criteria for qRT-PCR primer design (*SI Appendix*, *SI Materials and Methods*) revealed that transcript levels of all other *CYP71D* clade members in *CYP71D365*-silenced leaf and stem tissue were unchanged (*SI Appendix*, Fig. S11 *C* and *D*). This analysis does demonstrate the high specificity of the VIGS approach, with genes showing up to 70% identity being unaffected. The cause of the decrease in peplusol levels in *CYP71D365*-silenced leaf and stem tissue remains to be determined.

VIGS of *EpSDR-5* resulted in a statistically significant (1.6- to 1.8-fold) reduction of jatrophane levels in both leaves and stems (Fig. 4*C* and Table S4) and, consistent with this, an over 2-fold reduction in transcript levels (*SI Appendix*, Fig. S11 *E* and *F*). Most interestingly, reduction in jatrophanes was accompanied by a corresponding increase (1.4- to 1.7-fold) in the level of ingenanes in both tissues analyzed. Both the nonacetylated and acetylated forms of IM increase in the *EpSDR-5*–silenced tissues (Table S4). Levels of peplusol remained unchanged (Fig. 4*C* and Table S4). Jolkinol C, casbene, and casbene oxidation products remained undetectable in any of the tissues analyzed.

To check for off-target effects due to VIGS, we measured transcript levels of the other six members of the *E. peplus* latex-specific SDRs in *EpSDR-5*–silenced tissue. With very stringent criteria for qRT-PCR primer design (*SI Appendix*, *SI Materials and Methods*), we found that transcript levels of the six other SDRs remained unchanged in *EpSDR-5*–silenced leaf and stem tissue (*SI Appendix*, Fig. S11 *E* and *F*).

Based on these results, we propose a model whereby in *E. peplus* the biosynthetic pathway to jatrophanes starts with casbene and proceeds via jolkinol C and jolkinol E, involving the action of all three genes targeted by VIGS in the current study (Fig. 4*D*). According to this model, ingenanes are also derived from casbene and jolkinol C, but the biosynthetic pathway diverges at jolkinol C and does not proceed via jolkinol E (Fig. 4*D*). A block in the pathway to jatrophanes in *EpSDR-5*–silenced material results in a redirection of flux from jolkinol C into ingenanes including the medicinal compound IM. Biosynthesis of jatrophanes from jolkinol E requires opening a cyclopropyl ring retained in a latyrane backbone from the casbene precursor. This reaction is almost certainly enzymatic and could be catalyzed by alpha/beta hydrolases, which is an enzyme class that has been involved in the breaking of carbon–carbon bonds (42) and opening of heteroaromatic rings, including epoxides (43, 44). Further biosynthetic steps to jatrophanes would require multiple oxidations at position C5, 7, 8, and 9 yielding hydroxyl groups that could be modified further with a range of functional groups present on the backbone molecule (10). Biosynthesis of ingenanes is likely to proceed via a different route, where jolkinol C could be a direct precursor (Fig. 4*D*). Oxidations at C4, 5, and 20 could occur in conjunction with a Δ12,13-double bond reduction and introduction of a Δ1,2 double bond to form ingenol from jolkinol C. Previous work has suggested that the biosynthesis of ingenanes from a lathyrane precursor may involve a tigiliane intermediate (15, 21), which would retain the C3-keto group present in jolkinol C. In any case, the C3-keto reduction that enables further modifications present in *E. peplus* ingenanes is likely to be catalyzed by an enzyme other than EpSDR-5, as the substrate for such a reaction would be structurally very different to jolkinol C. Although the biosynthetic routes proposed above are speculative and need to be experimentally validated, we have recently reported that *JcAlkenalReductase 3* encodes an enzyme with a Δ12,13-double bond reductase activity that converts jolkinol C [1] and epi-jokinol C [2] to 12,13-dihydro-jolkinol C and 12,13-dihydro-epi-jolkinol C, respectively (29). It would be interesting to establish if the *E. peplus* genome encodes a functional equivalent of *JcAlkenalReductase 3* and if that gene is involved in the biosynthesis of *E. peplus* ingenanes.

It is noteworthy that *EpSDR-5,* which is not associated with either gene cluster 1 or 2, is predominantly expressed in latex, while the genes involved in jolkinol C biosynthesis encoded in gene clusters 1 and 2 (*Casbene synthase*, *CYP726A19*, *CYP726A4*, *CYP71D365*, and *EpADH1*), are most highly expressed in roots and/or stems with lower expression in latex. Coordinate gene expression is considered a main driver for clustering of genes associated with plant specialized metabolism (28), and one could speculate that the different expression patterns for the jolkinol C biosynthetic genes and *EpSDR-5* could be due to the jolkinol C precursor being synthesized mainly in stems and actively transported to laticifers to undergo conversion to jatrophanes via jolkinol E or to ingenanes via an alternative route. Such compartmentalization of specialized metabolism in internal (resin ducts and laticifers) and external secretory structures (glandular secretory trichomes) has been reported for potentially phytotoxic products or those involved in defense responses against herbivores and pathogens (45).

## Conclusions

The short life cycle (around 10 wk), relatively small genome size (27), and rich diversity of the di- and triterpenoids compared to other *Euphorbia* genus members (46) make *E. peplus* an attractive model plant for elucidation of the complex pathways to an array of bioactive terpenoids. The ‘omics approaches described herein have led to the identification of multiple candidate genes and the functional characterization of EpSDR-5, which encodes a short-chain dehydrogenase/reductase that produces jolkinol E from jolkinol C. We have developed VIGS in *E. peplus* and deployed this method to show that both ingenanes and jatrophanes are derived from casbene and jolkinol C but their biosynthetic pathways diverge at that point with the biosynthesis of jatrophanes, unlike ingenanes, requiring jolkinol E production. These findings shed light on the biosynthetic pathways to the two most abundant classes of diterpenoids in *E. peplus*, providing a valuable framework to facilitate the elucidation of the remaining steps in these two pathways.

## Materials and Methods

Full details of plant material and GenBank accession numbers for gene and BAC sequences are detailed in *SI Appendix*, *SI Materials and Methods*. RNAseq data are available as GenBank BioProject ID PRJNA81996. Liquid chromatography–mass spectrometry (MS) and gas chromatography-MS–based metabolomic analysis were conducted on extracts from 8-wk-old *E. peplus* plants to determine metabolite composition. NMR was conducted to determine the structure of major compounds in *E. peplus* latex/stems. RNAseq was conducted on five different tissues and used to identify candidate genes associated with diterpenoid biosynthesis. BAC library screening was conducted to identify diterpenoid gene clusters. *N. benthamiana* and *S. cerevisiae* were used as heterologous hosts to determine gene function. VIGS was used to confirm in vivo gene function.

## Supplementary Material

Supplementary File

## Data Availability

Gene and BAC DNA sequence data have been deposited in GenBank (gene accessions MW594404–MW594410, MW594413, MW594414, and MW594418–MW594434 and BAC accessions MW775845–MW775849). RNAseq data are available as GenBank BioProject ID PRJNA81996. All other study data are included in the article and/or supporting information.
