# Supplementary material for: Gene discovery and virus-induced gene silencing reveal branched pathways to major classes of bioactive diterpenoids in Euphorbia peplus
Source: Proc Natl Acad Sci U S A. 2022 May 18;119(21):e2203890119. doi: 10.1073/pnas.2203890119 (PMC9173813; doi:10.1073/pnas.2203890119)
Supplement: Supplementary File [file pnas.2203890119.sapp.pdf]

Supplementary Information for

Gene discovery and virus induced gene silencing reveal branched pathways to the major classes of bioactive diterpenoids in *Euphorbia peplus*

Tomasz Czechowski[1], Edith Forestier[1], Sandesh H. Swamidatta[1], Alison D. Gilday[1], Amy Cording[1], Tony R. Larson[1], David Harvey[1], Yi Li[1], Zhesi He[1], Andrew J. King[1,3], Geoffrey D. Brown[2] and Ian A. Graham[1*]

[1]Centre for Novel Agricultural Products, Department of Biology, University of York, Heslington, York, YO10 5DD (UK)

[2]Department of Chemistry, University of Reading, Whiteknights, Reading, RG6 6AD (UK)

[3]Current address: Jazz Pharmaceuticals, Sovereign House, Vision Park, Histon, Cambridge CB24 9BZ (UK)

[*] For correspondence

Email: ian.graham@york.ac.uk,

This PDF file includes:

Supplementary text
Figures S1 to S12
Tables S1 to S9
List of *S. cerevisiae* codon optimised plant gene sequences
SI References

SI Materials and Methods

Plant material

Euphorbia peplus seeds were obtained from All Rare Herbs (Australia). *Euphorbia peplus* plants were grown in P1 trays filled with F2 compost under 16 h / 8 h light and 25 ^{0}C / 22 ^{0}C day/night regime for 8 weeks. Latex samples were collected by cutting main stems with a scalpel and collecting white sap by pipette into an Eppendorf tube (5-50 µL per plant) followed by freezing in liquid N_2 (LN_2). Main stems, side stems, leaves, developing pods and roots were pooled from 8-10 plants and immediately frozen in LN_2. Roots were washed in distilled water and dried on tissue paper before freezing in LN_2. Mature seeds were collected from plants that were self-pollinated 4 weeks after germination.

Nicotiana benthamiana seeds were obtained from Dr David Zaitlan (University of Kentucky). Plants were grown in P15 trays as above for *E. peplus* for 4 weeks before being subjected to vacuum-infiltration with *A. tumefaciens.*

Euphorbia peplus metabolomics analysis

Roots, leaves, main and side stems, pods and mature seeds were ground to a fine powder with a steel bead using a Retsch II homogenizer (Qiagen, Hilden, Germany) for 30 sec at 30 Hz. 200 mg of powdered tissue or 200 µL of latex was extracted from five biological replicates for each of the seven tissues with 1 mL of 100% ethyl acetate (Rathburn Chemicals, UK) with 10 µg / ml of phorbol 12-myristate 13-acetate (PMA) standard (LC Laboratories, Worburn, MA, US) and 10 µg / ml of eicosane standard (Merck, Gillingham, UK) added in 2 mL round bottom Eppendorf tubes with vigorous shaking (1,500 rpm) overnight. Extracts were centrifuged at 13,000 rpm for 2 min in a table-top centrifuge and transferred to fresh Eppendorf vials and quantified. Aliquots of 200 µL were transferred to 1.5 ml tapered glass vials and analysed by GC-MS as previously described (1). Remaining sample volumes were dried in a Genevac™ EZ-2 Elite Concentrator (Genevac, Ipswich, UK), resuspended in 200 µL of 100% methanol (Rathburn Chemicals, UK) and transferred to 1.5 ml tapered glass vials. Samples were analysed by LC-MS as previously described (1).

Preparation and identification of main constituents of *E. peplus* aerial tissues.

1 kg of the aerial parts of *E. peplus* plants were harvested, frozen in LN_2 and freeze-dried for 7 days. Freeze-dried tissue was ground to a fine powder as detailed in the previous section and extracted with 15 volumes of 100% ethyl acetate (Rathburn Chemicals, UK). Ethyl acetate was removed by rotary evaporation to yield 1.15 g of a dark green oily residue which was taken up in 20 ml of an *n*-hexane:ethyl acetate mixture (80:20). The extract was then applied to a 40 g Grace

Resolve silica column and fractions collected using a 0-100 % ethyl acetate in hexane gradient, followed by isocratic 100% ethyl acetate and 100% methanol. This method yielded 13 mg of ingenol-3-angelate-20-acetate, 45 mg of a mixture of β-sitosterol/20-deoxyingenol-3-angelate mixture and 3 mg of peplusol. Fractions containing jatrophanes 1-4, and ingenol-3-angelate were purified further on a C18-3.5uM 250 X 10 mm preparative reversed-phase HPLC column (Interchim) and fractions collected using a 0-80% gradient of Solvent B in Solvent A, followed by isocratic 40% Solvent B and 60% Solvent C, followed by isocratic 80% Solvent A and 20% Solvent B (where Solvent A is 80% (v/v) methanol in water with 0.2% (v/v) formic acid; Solvent B is 100% methanol with 0.2% (v/v) formic acid; and Solvent C is 100% acetone). This method yielded 1 mg of jatrophane 1, 2 mg of jatrophane 2, 1 mg of jatrophane 3, 1 mg of jatrophane 4 and 1 mg of ingenol-3-angelate.

NMR analysis of the main constituents of *E. peplus* aerial tissues

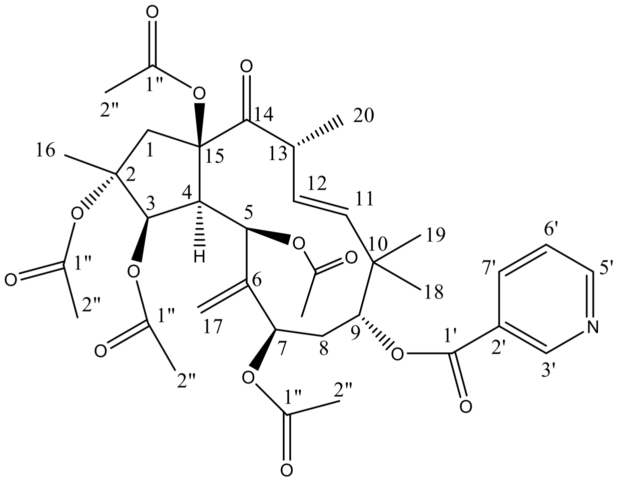

Jatrophane 1

NMR data for jatrophane 1 (2,3,5,7,15-pentaaacetoxy-9-nicotinoyloxy-jatropha-6(17),11*E*-diene-14-one): ^{1}H NMR (700 MHz, $CDCl_3$): δ 9.22 (dd, J = 2.1, 0.8 Hz, 1H (H-3' Nic)), 8.80 (dd, J = 4.8, 1.8 Hz, 1H (H-5' Nic)), 8.23 (ddd, J = 7.9, 2.1, 1.8 Hz, 1H (H-7' Nic)), 7.39 (ddd, J = 7.9, 4.8, 0.8 Hz, 1H (H-6' Nic)), 5.95 (d, J = 15.9 Hz, 1H (H-11)), 5.94 (br, 1H, (H-5)), 5.65 (dd, J = 15.9, 9.3 Hz, 1H (H-12)), 5.42 (br dd, J = 3.5, 1.1 Hz, 1H (H-3)), 5.18 (s, 1H (H-17)), 5.15 (d J = 1.5 Hz, 1H, (H-17)), 5.07 (dd, J = 8.0 ,3.3 Hz, 1H (H-9)), 4.99 (dd, J = 6.4, 4.8 Hz, 1H (H-7)), 3.82 (dd, J = 16.1, 1.1 Hz, 1H (H-1)), 3.52 (dq, J = 9.3, 6.7 Hz, 1H (H-13)), 2.96 (d, J = 3.5 Hz, 1H (H-4)), 2.16 (s, 3H (7-OAc C-2'')), 2.15 (s, 3H (3-OAc, C-2'')), 2.14 (s, 3H (15-OAc C-2'')), 2.13 (s, 3H (2-OAc C-2'')), 1.97 (d, J = 16.1 Hz, 1H (H-1)), 1.63 (s, 3H (5-OAc C-2'')), 1.49 (s, 3H (H-16)), 1.17 (d, J = 6.7 Hz, 3H, (H-20)), 1.16 (s, 3H (H-18/19)), 1.11 (s, 3H (H-18/19)); ^{13}C NMR (175 MHz, $CDCl_3$): δ 211.2 (C-14), 170.5 (2-OAc C-1''), 170.3 (15-OAc C-1''), 169.5 (5-OAc C-1''), 169.4 (7-OAc C-1''), 168.9 (3-OAc C-1''), 164.1 (Nic C-1'), 153.7 (Nic C-5'), 151.1 (Nic C-3'), 146.1 (C-6), 138.7 (C-

11), 136.9 (Nic C-7’), 130.5 (C-12), 125.8 (Nic C-2’), 123.3 (Nic C-6’), 111.5 (C-17), 92.6 (C-15), 86.6 (C-2), 78.2 (C-3), 75.8 (C-9), 68.8 (C-5), 68.2 (C-7), 49.4 (C-4), 46.9 (C-1), 43.7 (C-13), 40.7 (C-10), 34.8 (C-8), 27.0 (C-18/19), 22.3 (C-18/19), 22.2 (15-OAc C-2’’), 21.3 (3-OAc C-2’’), 21.2 (7-OAc C-2’’), 21.1 (2-OAc C-2’’), 20.6 (5-OAc C-2’’), 19.6 (C-20), 18.0 (C-16).

HRMS (m/z) [M+H]+ calcd. for $C_{36}H_{45}NO_{13}$, 700.2969 ; found, 700.2970

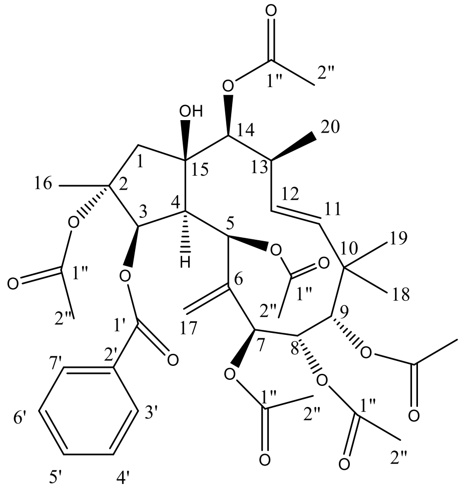

Jatrophane 2

NMR data for jatrophane 2 (2,5,7,8,9,14-hexaacetoxy-3-benzoyloxy-15-hydroxy-jatropha-6(17),11*E*-diene): ^{1}H NMR (700 MHz, $CDCl_3$): δ 8.08 (dd, J = 8.1, 1.4 Hz, 2H (H-3’ and H-7’ Bz)), 7.58 (tt, J = 7.4, 1.4 Hz, 1H (H-5’ Bz)), 7.44 (dd, J = 8.1 ,7.4 Hz, 2H (H-4’ and H-6’ Bz)), 5.88 (d, J = 5.2 Hz, 1H (H-3)), 5.87 (d, J = 15.9 Hz, 1H, (H-11)), 5.81 (d, J = 3.8 Hz, 1H (H-5)), 5.66 (dd, J = 15.9, 9.1 Hz, 1H (H-12)), 5.50 (s, 1H (H-7)), 5.26 (s, 1H, (H-8)), 5.11 (s, 1H (H-14)), 4.97 (s, 1H (H-9)), 4.91 (br, 1H (H-17), 4.51 (d, J = 1.1 Hz, 1H (H-17), 3.59 (s, 1H, 15-OH), 3.29 (dd, J = 5.2, 3.8 Hz, 1H (H-4)), 2.68 (d, J = 15.0 Hz, 1H (H-1)), 2.65 (dq, J = 9.1, 7.1 Hz, 1H (H-13)), 2.23 (d, J = 15.0 Hz, 1H (H-1)), 2.16 (s, 3H (2-OAc C-2’’)), 2.15 (s, 3H (7-OAc C-2’’)), 2.11 (s, 3H (14-OAc C-2’’)), 2.07 (s, 3H (9-OAc C-2’’), 1.99 (s, 3H (8-OAc C-2’’), 1.92 (s, 3H (5-OAc C-2’’), 1.55 (s, 3H (H-16)), 1.35 (s, 3H (H-18/19)), 1.15 (d, J = 7.1 Hz, 3H, (H-20)), 0.94 (s, 3H (H-18/19)); ^{13}C NMR (175 MHz, $CDCl_3$): δ 170.6 (14-OAc C-1’’), 170.5 (2-OAc C-1’’), 169.8 (8-OAc C-1’’), 169.7 (9-OAc C-1’’), 168.9 (7-OAc C-1’’), 168.1 (5-OAc C-1’’), 164.8 (Bz C-1’), 143.4 (C-6), 134.2 (C-11), 133.3 (Bz C-5’), 131.3 (C-12), 130.0 (Bz C-2’), 129.7 (Bz C-3’ and C-7’), 128.5 (Bz C-4’ and C-6’), 110.1 (C-17), 88.3 (C-2), 83.8 (C-15), 80.5 (C-9), 80.2 (C-3), 79.4 (C-14), 71.5 (C-5), 70.6 (C-8), 67.9 (C-7), 50.4 (C-1), 44.6 (C-4), 40.8 (C-10), 37.3 (C-13), 26.4 (C-18/19), 23.7 (C-18/19), 23.6 (C-20), 22.3 (2-OAc C-2’’), 21.9 (C-16), 21.0 (7-OAc C-2’’), 20.8 (5-OAc C-2’’), 20.62 (8-OAc C-2’’), 20.59 (14-OAc C-2’’), 20.5 (9-OAc C-2’’).

HRMS (m/z) [M+H]+ calcd. for $C_{39}H_{50}O_{15}$, 759.3228; found, 759.3224

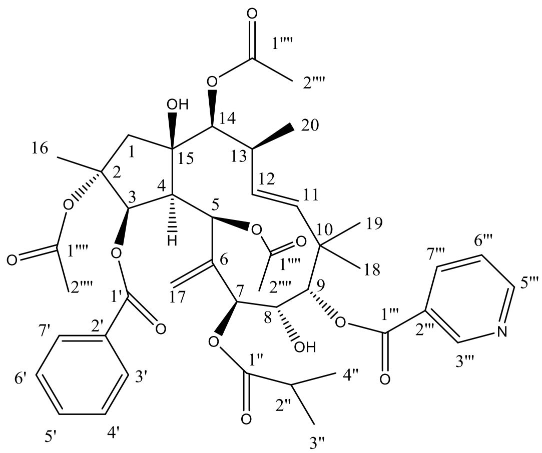

Jatrophane 3

NMR data for jatrophane 3 (2,5,14-triacetoxy-3-benzoyloxy-8,15-dihydroxy-7-isobutyryloxy-9-nicotinoyloxyjatropha-6(17),11*E*-diene): ^{1}H NMR (700 MHz, $CDCl_3$): δ 9.31 (dd, J = 2.0, 0.5 Hz, 1H (H-3''' Nic)), 8.82 (dd, J = 4.8, 1.8 Hz, 1H (H-5''' Nic)), 8.37 (ddd, J = 8.0, 2.0, 1.8 Hz, 1H (H-7''' Nic)), 8.06 (dd, J = 8.3, 1.2 Hz, 2H (Bz H-3' and H-7')), 7.57 (tt, J = 7.4, 1.2 Hz, 1H (Bz H-5')), 7.43 (dd, J = 8.3, 7.4 Hz, 2H (Bz H-4' and H-6')), 7.43 (m, 1H, Nic H-6'''), 6.17 (d, J = 15.9 Hz, 1H (H-11)), 5.93 (d, J = 5.7 Hz, 1H, (H-3)), 5.80 (br d, J = 3.8 Hz (H-5)), 5.68 (dd, J = 15.9, 9.6 Hz, 1H (H-12)), 5.40 (s, 1H (H-7)), 5.16 (s, 1H (H-14)), 5.08 (s, 1H, (H-9)), 4.83 (br, 1H (H-17)), 4.45 (d, J = 1.1 Hz, 1H (H-17)), 4.15 (d, J = 11.1 Hz, 1H (H-8)), 3.75 (dd, J = 5.7, 3.8 Hz, 1H (H-4)), 3.66 (s, 1H, (15-OH)), 2.94 (d, J = 11.1 Hz, 1H (8-OH)), 2.89 (dq, J = 9.6, 7.1 Hz, 1H (H-13)), 2.86 (d, J = 14.3 Hz, 1H (H-1)), 2.22 (s, 3H (2-OAc C-2''''), 2.11 (s, 3H (14-OAc C-2'''')), 2.09 (m, 1H (H-1)), 2.04 (s, 3H (5-OAc C-2''''), 2.00 (m, 1H (^{i}Bu H-2''), 1.48 (s, 3H (H-16)), 1.37 (s, 3H, H-18/19), 1.17 (d, J = 7.1 Hz, 3H (H-20)), 1.07 (s, 3H (H-18/19)), 0.94 (d, J = 7.1 Hz, 3H, (^{i}Bu H-3''/4'')), 0.48 (d, J = 6.9 Hz, 3H, (^{i}Bu H-3''/4'')); ^{13}C NMR (175 MHz, $CDCl_3$): δ 174.1 (^{i}Bu C-1''), 171.1 (2-OAc C-1''''), 170.4 (14-OAc C-1''''), 168.5 (5-OAc C-1''''), 165.8 (Nic C-1'''), 164.8 (Bz C-1'), 154.0 (Nic C-5'''), 151.6 (Nic C-3'''), 144.1 (C-6), 137.6 (Nic C-7'''), 133.8 (C-11), 133.1 (Bz C-5'), 131.5 (C-12), 130.1 (Bz C-2'), 129.6 (Bz C-3' and C-7'), 128.4 (Bz C-4' and C-6'), 125.0 (Nic C-2'''), 123.3 (Nic C-6'''), 109.1 (C-17), 88.5 (C-2), 86.8 (C-9), 84.4 (C-15), 80.5 (C-3), 79.4 (C-14), 71.9 (C-5), 70.3 (C-8), 68.1 (C-7), 49.7 (C-1), 44.8 (C-4), 40.4 (C-10), 37.6 (C-13), 33.5 (^{i}Bu C-2''), 27.5 (C-18/19), 23.6 (C-16), 23.5 (C-20), 22.5 (2-OAc C-2''''), 21.1 (5-OAc C-2''''), 20.5 (14-OAc C-2''''), 19.1 (^{i}Bu C-3''/4''), 17.4 (^{i}Bu C-3''/4'').

HRMS (m/z) [M+H]+ calcd. for $C_{43}H_{53}NO_{14}$, 808.3544; found, 808.3545

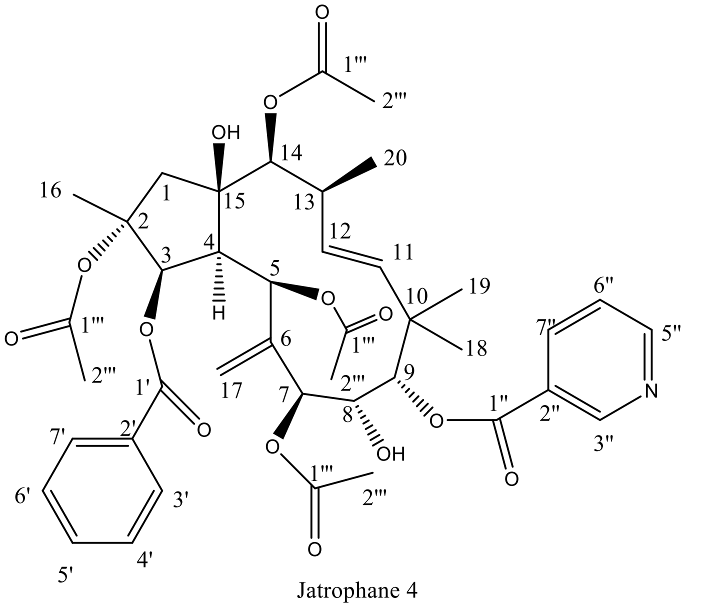

Jatrophane 4

NMR data for jatrophane 4 (2,5,7,14-tetraacetoxy-3-benzoyloxy-8,15-dihydroxy-9-nicotinoyloxyjatropha-6(17),11*E*-diene): ^{1}H NMR (700 MHz, $CDCl_3$): δ 9.32 (dd, J = 1.7, 0.5 Hz, 1H (H-3'' Nic)), 8.83 (dd, J = 4.8, 1.3 Hz, 1H (H-5'' Nic)), 8.38 (ddd, J = 8.1, 2.0, 1.7 Hz, 1H (H-7'' Nic)), 8.06 (dd, J = 8.2, 1.3 Hz, 2H (Bz H-3' and H-7')), 7.57 (tt, J = 7.6, 1.3 Hz, 1H (Bz H-5')), 7.43 (dd, J = 8.2, 7.6 Hz, 2H (Bz H-4' and H-6')), 7.42 (ddd, J = 8.1, 4.8, 0.5 Hz, 1H (H-6'' Nic)), 6.14 (d, J = 15.9 Hz, 1H (H-11)), 5.98 (br d, J = 5.7 Hz, 1H (H-3)), 5.81 (br d, J = 4.0 Hz, 1H, (H-5)), 5.67 (dd, J = 15.9, 9.6 Hz, 1H (H-12)), 5.30 (s, 1H (H-7)), 5.11 (s, 1H (H-9)), 4.78 (s, 1H (H-17)), 4.48 (s, 1H, (H-17)), 4.14 (d, J = 10.6 Hz, 1H (H-8)), 3.76 (dd, J = 5.7, 4.0 Hz, 1H (H-4)), 3.66 (s, (15-OH)), 2.84 (d, J = 14.4, 1H (H-1)), 2.84 (m, 1H (H-13)), 2.82 (d, J = 10.6, 1H (8-OH)), 2.22 (s, 3H (14-OAc C-2''')), 2.11 (s, 3H (2-OAc C-2'''), 2.08 (d, J = 14.4 Hz, 1H (H-1)), 2.05 (s, 3H (7-OAc C-2''')), 2.04 (s, 3H (7-OAc C-2''')), 1.48 (s, 3H (H-16)), 1.36 (s, 3H (H-18/19)), 1.17 (d, J = 6.9 Hz, 3H, (H-20)), 1.08 (s, 3H (H-18/19)); ^{13}C NMR (175 MHz, $CDCl_3$): δ 171.1 (14-OAc C-1'''), 170.4 (2-OAc C-1'''), 168.5 (5-OAc C-1'''), 168.0 (7-OAc C-1'''), 165.6 (Nic C-1''), 164.9 (Bz C-1'), 154.1 (Nic C-5''), 151.5 (Nic C-3''), 143.3 (C-6), 137.3 (Nic C-7''), 133.8 (C-11), 133.2 (Bz C-5'), 131.7 (C-12), 129.7 (Bz C-3' and C-5'), 128.4 (Bz C-4' and C-6'), 125.0 (Nic C-2''), 123.3 (Nic C-6''), 109.0 (C-17), 88.6 (C-2), 86.3 (C-9), 84.4 (C-15), 80.7 (C-3), 79.4 (C-14), 72.0 (C-5), 70.1 (C-8), 69.3 (C-7), 49.5 (C-1), 44.8 (C-4), 40.4 (C-10), 37.6 (C-13), 27.2 (C-18/19), 23.6 (C-20), 23.5 (C-16), 23.1 (C-18/19), 22.5 (14-OAc C-2'''), 21.1 (7-OAc C-2'''), 20.5 (5-OAc C-2'''), 20.3 (2-OAc C-2''').

HRMS (m/z) [M+H]+ calcd. for $C_{41}H_{49}NO_{14}$, 780.3231; found, 780.3232

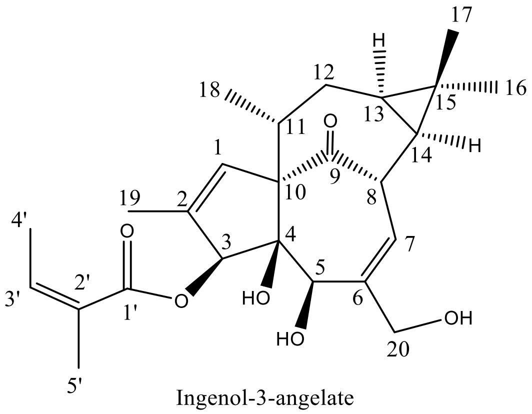

Ingenol-3-angelate
(Ingenol mebutate)

NMR data for ingenol-3-angelate (ingenol mebutate; IM): ^{1}H NMR (700 MHz, $CDCl_3$): δ 6.18 (q, J = 7.1 Hz, 1H (H-3' Ang)), 6.07 (br d, J = 4.5 Hz, 1H (H-7)), 6.04 (s, 1H (H-1)), 5.54 (s, 1H (H-3)), 4.24 (d, J = 4.0 Hz, 1H (5-OH)), 4.17 (d, J = 13.6 Hz, 1H (H-20)), 4.15 (d, J =13.6 Hz, 1H (H-20)), 4.14 (dd, J = 11.7, 4.5 Hz, 1H (H-8)), 4.07 (d, J = 4.0 Hz, 1H (H-5)), 3.48 (s, 1H (4-OH)), 2.54 (m, 1H (H-11)), 2.25 (ddd, J = 15.6, 8.8, 2.9 Hz, 1H (H-12)), 2.22 (br, 1H (20-OH)), 2.03 (d, J = 7.1 Hz, 3H (H-4' Ang), 1.93 (s, 3H (H-5' Ang)), 1.81 (s, 3H (H-19)), 1.76 (ddd, J = 15.6, 5.9, 5.7 Hz, 1H (H-12)), 1.09 (s, 3H (H-17)), 1.05 (s, 3H (H-16)), 0.97 (d, J = 7.2 Hz, 3H (H-18)), 0.95 (dd, J = 11.7, 8.5 Hz, 1H (H-14)), 0.70 (ddd, J = 8.8, 8.5, 5.9 Hz, 1H (H-13)); ^{13}C NMR (175 MHz, $CDCl_3$): δ 206.6 (C-9), 168.6 (Ang C-1'), 140.0 (Ang C-3'), 139.1 (C-6), 135.7 (C-2), 132.1 (C-1), 128.6 (C-7), 127.2 (Ang C-2'), 84.7 (C-4), 82.6 (C-3), 77.2 (C-5), 72.0 (C-10), 67.5 (C-20), 43.5 (C-8), 38.3 br (C-11), 31.1 (C-12), 28.6 (C-16), 24.0 (C-15), 23.4 (C-13), 23.0 (C-14), 20.8 (Ang C-5'), 17.3 br (C-18), 16.0 (Ang C-4), 15.6 (C-17), 15.5 (C-19)

HRMS (m/z) [M+H]+ calcd. for $C_{25}H_{34}O_6$, 431.2434; found, 431.2422

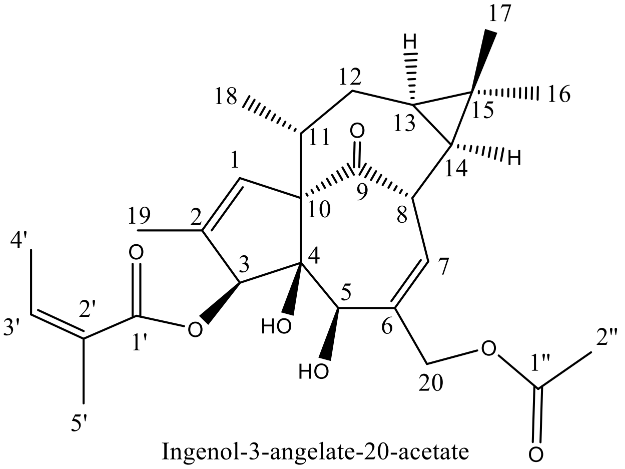

Ingenol-3-angelate-20-acetate

NMR data for ingenol-3-angelate-20-acetate: ^{1}H NMR (700 MHz, $CDCl_3$): δ 6.16 (qq, J = 7.2, 1.2 Hz, 1H (H-3’ Ang)), 6.08 (br, 1H (H-7)), 5.99 (br, 1H (H-1)), 5.54 (s, 1H (H-3)), 4.71 (d, J = 12.6 Hz, 1H (H-20)), 4.44 (d, J = 12.6 Hz, 1H (H-20)), 4.07 (m, 1H (H-8)), 3.86 (d, J = 6.4 Hz, 1H (H-5)), 3.54 (d, J = 6.4 Hz, 1H (5-OH)), 3.45 (s, 1H (4-OH)), 2.48 (m, 1H (H-11)), 2.23 (ddd, J = 15.5, 8.7, 2.5 Hz, 1H (H-12)), 2.03 (s, 3H (H-2’’ OAc)), 2.01 (dq, J = 7.2, 1.5 Hz, 3H (H-4’ Ang), 1.92 (dq, J = 1.2, 1.5 Hz, 3H (H-5’ Ang)), 1.80 (d, J = 1 Hz, 3H (H-19)), 1.75 (ddd, J = 15.5, 5.8, 5.8 Hz, 1H (H-12)), 1.07 (s, 3H (H-17)), 1.05 (s, 3H (H-16)), 0.95 (d, J = 7.1 Hz, 3H (H-18)), 0.89 (m, 1H (H-14)), 0.68 (ddd, J = 8.8, 8.5, 5.9 Hz, 1H (H-13)); ^{13}C NMR (175 MHz, $CDCl_3$): δ 206.3 (C-9), 171.1 (OAc C-1’’), 168.3 (Ang C-1’), 140.0 (Ang C-3’), 135.9 (C-2), 135.8 (C-6), 132.1 (C-1), 129.5 (C-7), 127.1 (Ang C-2’), 84.8 (C-4), 82.7 (C-3), 74.8 (C-5), 72.0 (C-10), 66.8 (C-20), 43.6 (C-8), 38.5 (C-11), 31.1 (C-12), 28.5 (C-16), 24.0 (C-15), 23.3 (C-13), 23.0 (C-14), 21.1 (OAc C-2’’), 20.8 (Ang C-5’), 17.2 (C-18), 15.9 (Ang C-4’), 15.6 (C-19), 15.5 (C-17).

HRMS (m/z) [M+H]+ calcd. for $C_{27}H_{36}O_7$, 473.2539; found, 473.2527

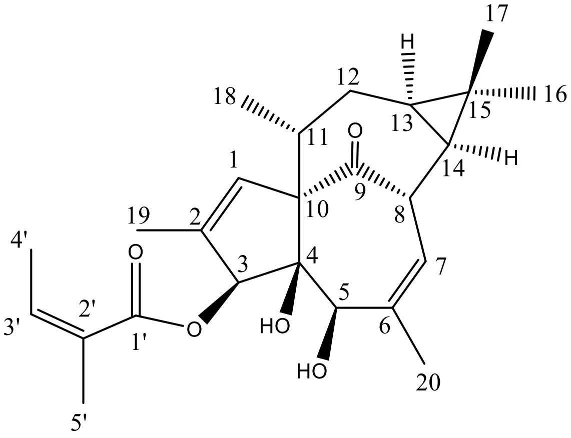

20-Deoxyingenol-3-angelate

NMR data for 20-deoxyingenol-3-angelate (as a mixture with β-sitosterol): δ 6.16 (qq, J = 7.3, 1.3 Hz, 1H (H-3’ Ang)), 6.07 (q, J = 1.4 Hz, 1H (H-1)), 5.75 (dq, J = 4.7, 1.2 Hz, 1H (H-7)), 5.49 (s, 1H (H-3)), 4.04 (ddq, J = 11.7, 4.7, 1.2 Hz, 1H (H-8), 3.70 (br, 1H (H-5)), 2.26 (m, 1H (H-12)), 2.01 (dq J = 7.3, 1.3 Hz, 3H (H-4’ Ang)), 1.93 (dq, J = 1.3, 1.3 Hz, 3H (H-5’ Ang)), 1.79 (d, J = 1.4 Hz, 3H (H-19)), 1.78 (br, 3H (H-20)), 1.74 (m, 1H (H-12)), 1.08 (s, 3H (H-17)), 1.04 (s, 3H (H-16)), 0.98 (m, 1H (H-11)), 0.91 (d, J = 6.6 Hz, 3H (H-18)), 0.91 (m, 1H (H-14)), 0.66 (m, 1H (H-13)); ^{13}C NMR (175 MHz, $CDCl_3$): δ 207.0 (C-9), 168.7 (Ang C-1’), 140.0 (Ang C-3’), 137.4 (C-6), 135.6 (C-2), 132.7 (C-1), 127.4 (Ang C-2’), 124.3 (C-7), 85.2 (C-4), 83.2 (C-3), 77.6 (C-5), 72.1 (C-10), 43.5 (C-8), 31.2 (C-12), 28.7 (C-16), 24.1 (C-15), 23.4 (C-14), 23.2 (C-13), 22.1 (C-20), 21.2 (C-11), 20.9 (Ang C-5’), 17.3 (C-18), 16.0 (Ang C-4’), 15.8 (C-19), 15.7 (C-17).

HRMS (m/z) [M+H]+ calcd. for $C_{25}H_{34}O_5$, 415.5344; found, 415.2475

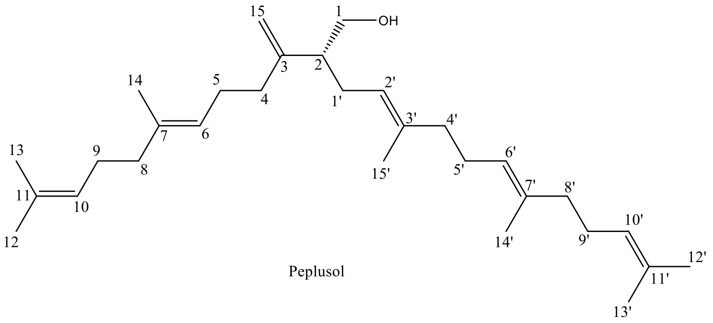

Peplusol

NMR data for peplusol as previously described (2)

HRMS (m/z) [M+H]+ calcd. for $C_{30}H_{50}O$, 427.3940; found, 427.3933

Euphorbia peplus transcriptomic analysis using RNAseq

Metabolite-profiling data was used to inform selection of *E. peplus* tissues for transcript profiling and differential expression analysis. RNA was extracted and RNAseq was performed on both latex-containing tissues (main stems, leaves and pods) and latex-free tissues (roots), as well as the latex itself as detailed below.

RNA samples were extracted from the roots, leaves, main stems, pods and latex of 8-week old *Euphorbia peplus*, with four biological replicates sampled for each tissue. Total RNA was extracted from root, leaf, main stems and pod tissue using the RNAeasy kit (Qiagen, Hilden, Germany) with "on column" DNAse digestion according to manufacturer's instructions. RNA was precipitated overnight using 0.1 volume of 3M sodium acetate and 2.5 volumes of 100% ethanol, washed twice the following day with 70% ethanol, air dried and re-suspended in water. Total RNA was extracted from latex using a modified protocol for the RNeasy Lipid Tissue kit (Qiagen, Hilden, Germany) with "on column" DNAse digestion.

cDNA libraries were prepared from 100 ng of total RNA from latex and 150 ng from the other four tissues using the NEBNext Ultra II Directional Library prep kit from Illumina in conjunction with the NEBNext® Poly(A) mRNA Magnetic Isolation Module, according to the manufacturer's instructions. A 12-minute fragmentation time was used when eluting mRNA from polyA magnetic beads. Amplification of the final libraries involved 12 cycles of PCR and the NEBNext multiplex Oligos from Illumina. The 20 samples were pooled at equimolar ratios, then run for paired-end 150 base sequencing on a HiSeq 3000 sequencer, at the University of Leeds Next Generation Sequencing facility.

HiSeq3000 generated 150bp paired-end reads were assembled into 201,887 contigs using Trinity RNAseq de novo assembly software package version 2.5.1 (3). Average contig length was 2 kb (median 1.3 kb). This assembly was used as reference to map reads from the individual libraries using BWA version 0.7 (4). After mapping, the raw read counts were carried out with SAMtools version 1.9 (5).

Counts per million (CPM) were calculated as: CPM = reads mapped to gene * 1/total number of reads * 10^6. Reads per kilobase per million mapped reads (RPKM) values were calculated as RPKM = number of reads / (contig length/1000 * total number of reads/1,000,000) for 106,578 contigs for which CPM values were higher than 1 in at least four out of 20 samples. RPKM values were used for the differential gene expression analysis. Differential gene expression was analysed in all tissues using four biological replicates. The methods in Bioconductor package EdgeR (6) were used to identify the differentially expressed genes. A multiple comparisons false discovery rate (FDR) of less than 0.05 was used to flag a gene being differentially expressed. Flags of "1" and "-1" were used to note positive and negatively expressed genes in the resulting excel file and can be filtered among comparisons. 1453 contigs expressed specifically in latex and/or stem tissue were selected this way as shown on Figure S3.

Functional annotations were assigned to the selected 1453 contigs using the SwissProt database. The number of candidate genes was reduced to 46 by focussing on those predicted on the basis of homology to encode enzymes expected to be involved in complex diterpenoid synthesis including cytochrome P450 monooxygenases, dioxygenases, alkenal reductases, hydroxylases, dehydratases, dehydrogenases and epoxidases as shown on Figure S3.

Construction of a *Euphorbia peplus* Bacterial Artificial Chromosome (BAC) library and identification and sequencing of clones corresponding to gene clusters.

A custom PCR-screenable BAC library was prepared by Amplicon Express (Pullman, WA) using either *HindIII* (9,216 clones with average insert size of 115 kb) or *BamHI* (9,216 with an average insert size of 133 kb) digested DNA inserted into a pCC1BAC vector (Epicentre, Madison, WI). To identify BACs containing gene clusters similar to those previously reported from *R. communis* and *J. curcas* (1, 7), PCR primers were designed against *CAsbene Synthase* (*EpCAS*), *EpCYP726A19* and *EpCYP726A4* (Table S5). BACs corresponding to positive clones were isolated using the NucleoBond BAC 100 kit (Macherey-Nagel, Düren, Germany). End sequences for these BAC clones were then obtained using dye-terminator sequencing, and used to design new screening primers to detect flanking clones (Table S5). The BACs were then initially sequenced with Oxford Nanopore's MinIon sequencer (Oxford, UK) using R6 version flowcells and reagents. The BAC vector sequence was then removed using cross_match4, assembled using canu5 and then polished using nanopolish (8). Functional annotations were assigned for the predicted genes using the SwissProt database. Sequences of the predicted Cytochrome P450 oxidases were named

according to the P450 Nomenclature Committee (Dr. David Nelson, University of Tennessee Health Science Center).

***Euphorbia peplus* candidate gene cloning and transient gene expression in *Nicotiana benthamiana*.**

cDNA was synthesised using total RNA from 100 ng of *E. peplus* latex or stems total RNA using Superscript II reverse transcriptase (Invitrogen, Carlsbad, CA) and random hexamer primers (Invitrogen, Carlsbad, CA). The open reading frame for each gene was then amplified and inserted into the pEAQ-HT expression vector (9) via In-Fusion cloning tools (TaKaRa bio Inc. Kusatsu, Japan), according to the manufacturer's protocol using the primers detailed in Table S6. In each instance, a 5'-AAAA-3' Kozak sequence was included immediately upstream of the start codon to enhance translation from the start codon. If the full open reading frame was not available we first perfomed 5‘ and 3‘ Rapid Amplification of cDNA Ends (RACE) using total RNA from *E. peplus* latex according to manufacturers instructions (Fisher Scientific UK Ltd, Loughborough, UK) using primers listed in Table S7. After confirming the presence of the correct inserts by Sanger sequencing, the expression vectors were transformed into *Agrobacterium tumefaciens* LBA4404 using the freeze-thaw method (10). For initial experiments to detect the production of novel diterpenoids, four-week old *Nicotiana benthamiana* plants were infiltrated by vacuum infiltration, using a vacuum pump to apply negative pressure at -0.9 Bar for 1 min with equal mixtures of *A. tumefaciens* cultures at a final OD 600_{nm} of 1.0 in infiltration buffer (10 mM $MgCl_2$, 200 μM acetosyringone and 0.015% Silwet L-77). Three isoprenoid precursor supply genes from *Arabidopsis thaliana* : *1-Deoxy-D-Xylulose 5-phosphate Synthase* (*AtDXS*), *4-Hydroxy-3-methylbut-2-enyl Diphosphate Reductase* (*AtHDR*) and *GeranylGeranyl PyroPhosphate Synthase* (*AtGGPPS*), which were shown to boost casbene production in *N. benthamiana* (11) were expressed with *J. curcas CAsbene Synthase* (*JcCAS)* and relevant *Euphorbiaceae* genes involved in the biosynthesis of jolkinol C / *epi*-jolkinol C (1) and jolkinol E / *epi*-jolkinol E. *Green Fluorescent Protein* from jellyfish *Aequorea victoria* (*AvGFP*) was used as a visual marker for gene expression in mesophyll cells. Five days after infiltration, all leaf material showing expression of *AvGFP* was harvested and flash frozen in liquid nitrogen. Freeze dried leaf material was ground for 30 sec with steel beads at 30 Hz minutes in a Retsch II homogenizer (Qiagen, Hilden, Germany) and extracted overnight with 1 ml of ethyl acetate containing 15 ug/ml of phorbol 12-myristate 13-acetate (PMA) with vigorous shaking. After centrifugation, ethyl acetate was removed by evaporation in a GeneVac personal evaporator (Genevac, Ipswich, UK), and the extract was re-dissolved in methanol. Samples were analysed by LC-MS as described previously (1, 11)

For the preparation of compounds for NMR analysis, multiple *Nicotiana benthamiana* plants were infiltrated by immersing in *A. tumefaciens* cultures resuspended in infiltration buffer and then applying a partial vacuum to a pressure of -0.9 Bar for 1 minute. Five days after infiltration, all leaf

material showing expression of *AvGFP* was harvested, flash frozen in liquid nitrogen and freeze dried for 5 days.

Preparation and identification of (3*E*,6*E*,11*E*)-8,9-dihydroxy-casba-3,6,11-trien-5-one [4]

14 g of freeze-dried *N. benthamiana* leaf material that had been infiltrated with: *AvGFP* (to visualize infiltrated material), *AtDXS, AtGGPP, AtHDR, JcCAS, JcCYP726A20, JcCYP71D495* and *EpSDR-1* was extracted once with 15 volumes of 100% ethyl acetate (Rathburn Chemicals, UK). The ethyl acetate was removed by rotary evaporation to yield 1.32 g of a green oily residue which was taken up in 20 ml of *n*-hexane:ethyl acetate mixture (80:20). The extract was then applied to a 40 g Grace Resolve silica column and fractions collected using a 0-100 % ethyl acetate in hexane gradient, followed by isocratic 100% ethyl acetate and 100% methanol. This method yielded 2 mg of (3*E*,6*E*,11*E*)-8,9-dihydroxy-casba-3,6,11-trien-5-one [**4**]**.**

Purified compounds were analysed using 1D- and 2D-NMR spectroscopy as described previously (1)

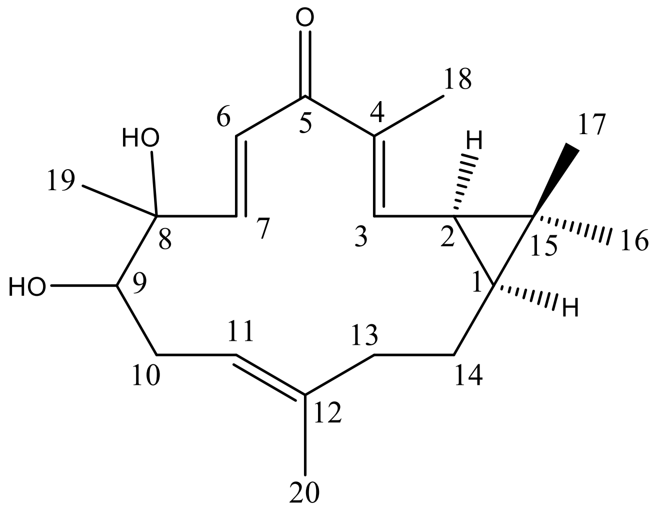

(3*E*,6*E*,11*E*)-8,9-dihydroxy-casba-3,6,11-triene-5-one [**4**]

NMR data for (3*E*,6*E*,11*E*)-8,9-dihydroxy-casba-3,6,11-triene-5-one [**4**]:^{1}H NMR (700 MHz, $CDCl_3$): δ 6.61 (d, J = 16.5 Hz, 1H (H-7)), 6.42 (d, J = 16.5 Hz, 1H (H-6)), 6.27 (d, J = 9.6 Hz, 1H (H-3)), 5.22 (dd, J = 7.0, 7.0 Hz, 1H (H-11), 3.84 (d, J = 4.6 Hz, 1H (9-OH)), 3.68 (dd, J = 6.0, 4.0 Hz, 1H (H-9)), 3.49 (br s, 1H, (8-OH)), 2.41 (m, 2H H-10)), 2.29 (m, 1H (H-13)), 2.16 (m, 1H (H-14)), 1.91 (d, J = 1 Hz, 3H (H-18)), 1.86 (m, 1H (H-13)), 1.62 (s, 3H (H-20)), 1.49 (m, 1H (H-2)), 1.34 (s, 3H (H-19)), 1.18 (s, 3H (H-16)), 1.17 (m, 1H (H-1)), 1.01 (s, 3H (H-17)), 0.86 (m, 1H (H-14)); ^{13}C NMR (175 MHz, $CDCl_3$): δ 195.3 (C-5), 146.6 (C-7), 143.6 (C-3), 138.5 (C-12), 138.0 (C-4), 128.6 (C-6),

119.8 (C-11), 78.7 (C-9), 75.4 (C-8), 39.3 (C-13), 33.5 (C-1), 31.7 (C-10), 29.0 (C-16), 27.6 (C-2), 26.0 (C-15), 25.5 (C-14), 24.9 (C-19), 16.1 (C-17), 15.7 (C-20), 12.3 (C-18).

HRMS (m/z) [M+H]+ calcd. for $C_{20}H_{30}O_3$, 319.2273 ; found, 319.2284

Preparation and identification of jolkinol E [5] and *epi*-jolkinol E [6]

15 g of freeze-dried *N. benthamiana* leaf material that had been infiltrated with *A. tumefaciens* cultures carrying: *AvGFP* (to visualize infiltrated material), *AtDXS, AtGGPP, AtHDR, JcCAS, JcCYP726A20, JcCYP71D495* and *EpSDR-1* was extracted once with 15 volumes of 100% ethyl acetate (Rathburn Chemicals, UK). The ethyl acetate was removed by rotary evaporation to yield 1.22 g green of an oily residue which was taken up in 20 ml of *n*-hexane:ethyl acetate mixture (80:20). The extract was then applied to a 40 g Grace Resolve silica column and fractions collected using a 0-100 % ethyl acetate in hexane gradient, followed by isocratic 100% ethyl acetate and 100% methanol. Fractions containing jolkinol E [**5**] and *epi*-jolkinol E [**6**] were further purified using C18-3.5 uM 250 X 10 mm preparative reversed-phase HPLC column using a 20-60% gradient of 100% methanol with 0.2% formic acid in 80% methanol:water with 0.2% formic acid, followed by isocratic 60% acetone in 40% methanol with 0.2% formic acid. This method yielded *ca.* 2 mg of jolkinol E [**5**] and *ca.* 1.5 mg of *epi*-jolkinol E [**6**]. Purified compounds were analysed using the 1D- and 2D-NMR approach as described before (1)

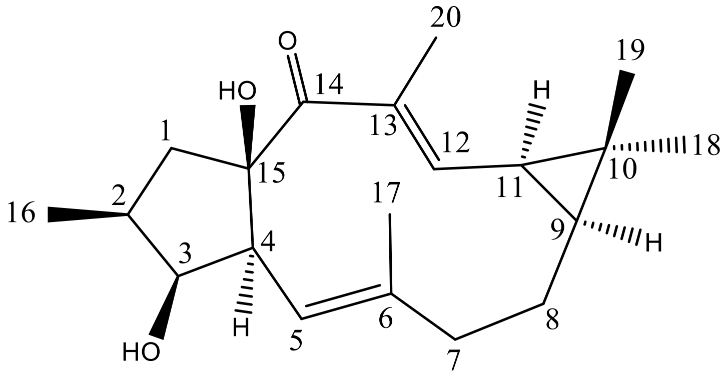

Jolkinol E [**5**]

NMR data for jolkinol E [**5**]: ^{1}H NMR (700 MHz, $CDCl_3$): δ 7.43 (d, J = 11.8 Hz, 1H (H-12)), 5.66 (d, J = 10.6 Hz, 1H (H-5)), 3.97 (br dd, J = 2.8, 2.8 Hz, 1H (H-3)), 3.45 (dd, J = 14.1, 9.5 Hz, 1H (H-1)), 2.87 (br, 1H, (15-OH)), 2.55 (br d, J = 13.1 Hz, 1H, (H-7)), 2.28 (dd, J = 10.6, 2.8 Hz, 1H (H-4)), 2.18 (m, 1H (H-8)), 2.02 (m, 1H, (H-2)), 1.83 (s, 3H (H-20)), 1.72 (m, 1H (H-7)), 1.57 (m, 1H (H-8)), 1.45 (dd, J = 11.8, 8.1 Hz, 1H (H-11)), 1.44 (d, J = 14.1 Hz, 1H (H-1)), 1.41 (s, 3H (H-17)), 1.18 (s, 3H, (H-18)), 1.11 (d, J = 6.8 Hz, 3H (H-16)), 1.09 (s, 3H (H-19)), 1.07 (m, 1H (H-9)); ^{13}C

NMR (175 MHz, $CDCl_3$): δ 197.9 (C-14), 151.6 (C-12), 141.8 (C-6), 132.3 (C-13), 120.1 (C-5), 92.7 (C-15), 81.2 (C-3), 53.2 (C-4), 46.0 (C-1), 38.7 (C-2), 36.6 (C-7), 35.0 (C-9), 29.8 (C-11), 29.2 (C-18), 28.2 (C-8), 24.8 (C-10), 20.9 (C-17), 16.3 (C-19), 14.3 (C-16), 12.3 (C-20).
HRMS (m/z) [M+H]+ calcd. for $C_{20}H_{30}O_3$, 319.2273; found, 319.2282

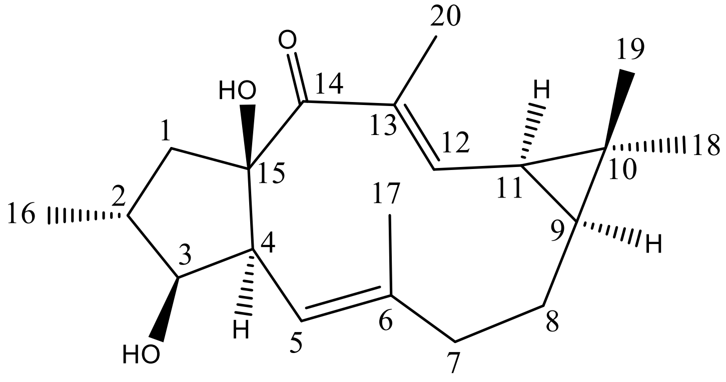

epi-Jolkinol E [**6**]

NMR data for *epi*-jolkinol E [**6**]: ^{1}H NMR (700 MHz, $CDCl_3$): δ 7.38 (d, J = 11.9 Hz, 1H (H-12)), 5.64 (d, J = 10.8 Hz, 1H (H-5)), 3.89 (dd, J = 4.3, 4.3 Hz, 1H (H-3)), 2.73 (dd, J = 14.4, 5.7 Hz, 1H (H-1)), 2.71 (br, 1H, (15-OH)), 2.56 (br d, J = 13.4 Hz, 1H, (H-7)), 2.52 (dd, J = 10.8, 4.3 Hz, 1H (H-4)), 2.31 (m, 1H (H-2)), 2.17 (m, 1H (H-8)), 2.04 (m, 1H, (H-1)), 1.84 (s, 3H (H-20)), 1.71 (d, J = 13.4 Hz, 1H (H-7)), 1.61 (m, 1H (H-8)), 1.46 (dd, J = 11.9, 8.5 Hz, 1H (H-11)), 1.42 (s, 3H (H-17)), 1.19 (s, 3H, (H-18)), 1.10 (s, 3H (H-19)), 1.09 (m, 1H, (H-9)), 1.07 (d, J = 7.2 Hz, 3H (H-16));^{13}C NMR (175 MHz, $CDCl_3$): δ 198.3 (C-14), 151.4 (C-12), 142.2 (C-6), 132.4 (C-13), 119.4 (C-5), 93.0 (C-15), 83.8 (C-3), 49.5 (C-4), 45.1 (C-1), 40.7 (C-2), 36.3 (C-7), 35.2 (C-9), 29.6 (C-11), 29.2 (C-18), 27.6 (C-8), 24.4 (C-10), 21.0 (C-17), 19.2 (C-16), 16.2 (C-19), 12.3 (C-20).
HRMS (m/z) [M+H]+ calcd. for $C_{20}H_{30}O_3$, 319.2273; found, 319.2282

Heterologous Expression in *Saccharomyces cerevisiae*

All pathway gene expression was done in CEN.PK2-1D strain background (MATα ura3-52; trp1-289; leu2-3,112; his3Δ 1; MAL2-8c; SUC2) and was purchased from EUROSCARF (accession no-30000B). We initially generated a strain Y214 (*DOS2::* P_{GAP1}-tHMG1/P_{ADH1}-MtGGPPS YER134C::P_{GAP1}-ALD6/P_{ADH1}- SEacs^{L641P}*, Δvba5)* by a combination of gene deletions and overexpressions to increase the flux through the mevalonate (MVA) pathway using a previously described strategies (12, 13). The deletion of *DOS2*, *YER134C* and *VBA5* loci were previously shown to increase MVA pathway flux (12). Truncated 3-hydroxy-3-methylglutaryl coenzyme A reductase (tHMGR) and *Methanothermobacter* GGPP synthase (codon optimised) gene cassettes were integrated at the *DOS2* locus. To increase the cytosolic acetyl Co-A pool, *S. cerevisiae* acetaldehyde

dehydrogenase (ALD6) and a mutated version of *Salmonella enterica* acetyl-CoA synthetase (SEacs^{L641P}) (13) gene cassettes were introduced at *YER134C* locus. The gene sequences for tHMG1 And ALD6 were amplified from CEN.PK2-1D genomic DNA.

Following a previously successful approach in producing casbene and oxidised casbenes in *S. cerevisiae* (14), we expressed a codon optimised *Jatropha curcas* casbene synthase gene as a tagged protein with maltose binding protein (MBP) at the N-terminus and an engineered FPP synthase that produces GGPP (erg20(F96C)) at the C-terminus to synthesise casbene (*nMBP-JcCAS-erg20F96C*) in *S.cerevisiae*. The individual fragments were synthesised as gBlock DNA fragments from IDT (Integrated DNA Technologies Inc.) and assembled into a *PmeI* digested modified pBEVY-L vector (Addgene# 51225) under the Gal10 promoter. All synthetic gene fragments were synthesized with overhangs allowing direct insertion into a *PmeI* digested modified pBEVY-L vector, without PCR amplification, via In-Fusion cloning tools (TaKaRa bio Inc. Kusatsu, Japan), according to the manufacturer's protocol.

Codon optimised versions of *Jatropha curcas* alcohol dehydrogenase 1 (*JcADH1*) and NADPH - cytochrome P450 reductase (*JcCPR*) were cloned under the Gal10 and Gal1 promoters respectively in pBEVY-L as a bidirectional gene cassette. Similarly codon optimised *Jatropha curcas* CYP726A20 and CYP71D495 were cloned under the Gal1 and Gal10 promoters respectively in pBEVY-L. All plasmids were propagated in Top10 *E. coli* cells (Invitrogen, USA) and sequence confirmed by Sanger sequencing (Eurofins, Europe). The gene cassettes were integrated at the chosen site in the *S.cerevisiae* genome using the CRISPR/Cas9 approach. The respective guide RNAs (14) were cloned into pRPR1_gRNA_hande_RPR1t vector (addgene#49014) as previously described (15). For genomic integration, the gene cassettes were PCR amplified with 50 bp homology arms to the respective target site. The purified PCR fragments were co-transformed with guide RNA plasmid and a plasmid containing Cas9 (addgene#43802, p414-TEF1p-Cas9-CYC1t) using a LiAc transformation protocol (16). The final jolkinol C producing strain was named Y264 (DOS2::P_{GAP1}-tHMG1, P_{ADH1}-MtGGPPS YER134C::P_{GAP1}-Ald6, P_{ADH1}-SEacs^{L641P} Δvba5, ARS308a::P_{GAL10}-*nMBP-JcCAS-erg20F96c*, ARS1021b::P_{GAL1}-*JcCPR1*, P_{GAL10}-JcADH1 ARS911b:: P_{GAL1}- JcCYP726A20, P_{GAL10}- JcCYP71D495) and used for screening *EpSDR-1* and *EpSDR-5*. The codon optimised *EpSDR-1* and *EpSDR-5* sequences were cloned under the Gal10 promoter in pBEVY-L. The constructs were transformed to Y264 by LiAC (17) method for screening. PCR primers used for cloning yeast constructs are listed in table S8.

S. cerevisiae strains were grown at 30 °C and 210 rpm in Synthetic Complete (SC) selective medium (SC-LEU) in shake flasks for 48h. 5 mL of culture was used for extraction with equal volume of ethyl acetate extracted overnight with vigorous shaking at room temperature. The ethyl acetate fraction was transferred to glass tubes and evaporated using a laboratory evaporator (EZ-2 series, Genevac Ltd.). The final pellet was dissolved in methanol and used for LC-MS analysis.

Virus-induced gene silencing (VIGS) of: *EpCH42, EpCAS, CYP71D365* and *EpSDR-5*

The tobacco rattle virus (TRV) bipartite system has frequently been used for VIGS. This involves two *Agrobacterium tumefaciens* strains, one containing pTRV1 encoding the replication and movement viral functions and the other containing pTRV2 which encodes the coat protein and the genes to be used for VIGS (18). This VIGS system was previously deployed in *J. curcas* along with a marker gene, Chlorota 42, to report silencing (19) The gene sequence of the *Euphorbia peplus* homologue of *J. curcas* Chlorota 42 marker gene was obtained by homology searching of the RNAseq data obtained from the five *E. peplus* tissues described above (*EpCH42*, gene_bank accession OL744077). A 163 bp fragment from the region of high protein homology between *EpCH42* and *JcCH42* (C-terminus) was PCR-amplified from the *E. peplus* leaf cDNA using primers containing the *EcoRI and BamHI* tails for InFusion cloning at the 5' and 3' ends respectively (Table S9). The amplified fragment was inserted into the pTRV2 vector digested with *EcoRI* and *BamHI* via In-Fusion cloning tools (TaKaRa bio Inc. Kusatsu, Japan), according to the manufacturer's protocol to form the pTRV2-*EpCH42*-Vigs construct

A 150bp fragment from the C-terminus of the E.peplus *Casbene Synthase* (*EpCAS*) was PCR-amplified from the *E. peplus* stem cDNA using primers with the *XhoII and SmaI* tails for InFusion cloning at the 5' and 3' ends respectively (Table S9). The amplified fragment was inserted into the pTRV2-*EpCH42*-Vigs construct digested with *XhoII* and *SmaI* via In-Fusion cloning tools (TaKaRa bio Inc. Kusatsu, Japan), according to the manufacturer's protocol, to form the pTRV2-*EpCAS:EpCH42*-Vigs construct. 139 bp and 148bp fragments from the non-conserved regions (C-terminus) of the *casbene-9-oxidase* (*CYP71D365*) and *EpSDR-5* respectively, were PCR-amplified from pEAQ-HT clones using primers with the *XhoII and SmaI* tails for InFusion cloning at the 5' and 3' ends respectively (Table S9). The amplified fragments were inserted into the pTRV2-*EpCH42*-Vigs construct digested with *XhoII* and *SmaI* via In-Fusion cloning tools (TaKaRa bio Inc. Kusatsu, Japan), according to the manufacturer's protocol to form the pTRV2-*CYP71D365:EpCH42*-Vigs and pTRV2-*EpSDR-5:EpCH42*-Vigs constructs.

After confirming the presence of the correct inserts by Sanger sequencing, the pTRV2 vectors were transformed into *A. tumefaciens* GV3101 using the freeze-thaw method (10). The *A. tumefaciens* GV3101 strains containing pTRV1, pTRV2-*EpCH42*-Vigs and one of the three target-gene constructs (pTRV2-*EpCAS:EpCH42*-Vigs, pTRV2-*CYP71D365:EpCH42*-Vigs or pTRV2-*EpSDR-5:EpCH42*-Vigs) were grown separately overnight at 28^{0}C, 220rpm, in 10ml LB medium containing kanamycin and gentamycin (50 mg/L) antibiotics. A 1 ml aliquot of overnight-grown cultures was inoculated in 50 ml of LB medium containing 10mM MES and 20 μM acetosyringone with kanamycin and gentamycin (50 mg/L) antibiotics and grown overnight at 28^{0}C, 220rpm. *A. tumefaciens* cells were harvested and re-suspended in the infiltration buffer (10mM MgCl2, 10mM MES, pH 5.6, 200 μM acetosyringone) to a final OD600 of 2.5 (for both pTRV1 and pTRV2 and its derivatives) and shaken for 2h at 28^{0}C, 100rpm. Equal volumes of the *A. tumefaciens*cultures carrying one of the pTRV2-derived constructs were mixed with pTRV1-carrying cultures. Mixed *A.*

tumefaciens cultures were infiltrated into both cotyledons of *E. peplus* seedlings 9-days after sowing, using a 1 ml syringe. Infiltrated plants were grown under 16 h / 8 h light and 25 ^{0}C / 22 ^{0}C day/night regime. Around 60-150 plants were infiltrated for pTRV2-*EpCH42*-Vigs control group and for each of the three pTRV2-*EpCH42*-Vigs derived target gene constructs. Three separate infiltrations were performed for each of the three target genes.

Chlorotic parts of leaf and stem samples were collected separately from plants 6 weeks post-infiltration. Fresh plant material was pooled from four to five independent plants to form one biological replicate, flash frozen in LN2 and stored in -80^{0}C. Five biological replicates were used for each of the two groups: pTRV2-*EpCH42*-Vigs controls and one of the three pTRV2-*EpCH42*-Vigs derived target gene constructs.

Metabolite and mRNA transcript analysis of VIGS treated *E. peplus* plants.

Plant material was ground in liquid nitrogen using a mortar and pestle. 150-250 mg of the ground fresh tissue was extracted using ethyl acetate with PMA internal standard and run on LC-MS as described above.

50-100 mg of the same ground fresh tissue was used to extract total RNA from stem tissue using the RNAeasy kit (Qiagen, Hilden, Germany) with "on column" DNAse digestion according to manufacturer's instructions. RNA was precipitated overnight using 0.1 volume of 3M sodium acetate and 2.5 volumes of 100% ethanol, washed twice the following day with 70% ethanol, air dried and re-suspended in water. Total RNA was extracted from leaf tissue using the CTAB-lithium chloride method (20) RNA samples were DNase treated and further purified using the on-column digestion protocol for the Qiagen RNeasy miniprep kit. cDNA was synthesized from 1µg of total RNA using random hexamers using Superscript II reverse transcriptase (Life Technologies). qPCR primers (Table S9) were designed using Geneious Prime® 2021.2.2 software. Real-time PCR was performed on CFX384 Real-Rime System (Bio-Rad Laboratories) using SsoAdvanced Universal SYBR® Green Supermix (Bio-Rad Laboratories). Each 10-µL reaction contained 2 µL of a 3-fold dilution of the cDNA synthesis reaction, 5µL of 2X Supermix, and primers at a final concentration of 250 nM. The cycling conditions included an initial activation step for 30 s at 98°C followed by 40 cycles of denaturation at 98°C for 10 s and annealing/extension at 60°C for 30s. Fluorescence data were acquired during the annealing/extension phase. A melt curve was obtained at the end of the amplification to allow confirmation of product specificity. C_T values were obtained using CFX Manager Software (Bio-Rad laboratories) and amplification efficiencies (E) obtained using LinReg PCR (21). Transcript abundance for the gene of interest (GOI) relative to housekeeping gene (HKG) was determined using the formula: GOI expression level = $(E_{GOI})^{\Delta C_T}$ / $(E_{HKG})^{\Delta C_T}$. Housekeeping genes were selected based on homology to HKGs used previously in *J. curcas* studies (22) and their stable expression across *E. peplus* stem, leaf, root and pod tissues. Two genes showing lowest coefficient of variation of RPKM values across RNAseq – a homologue of *Elongation Factor*

1α (*EpEF1α;* gene_bank accession OL744076) and a homologue of *SUMO-conjugating enzyme SCE1* (*EpSumo,* gene_bank accession OL744078), were selected for transcript normalization. Very strict designing criteria for qRT-PCR were used to ensure their specificity for the targeted transcripts for the SDR and CYP71D gene family members (Table S9). Each primer pair was tested in the qPCR containing 2 μL of targeted open reading frames cloned into pEAQ-HT expression vector (200pg), 5μL of 2X Supermix, and primers at a final concentration of 250 nM. The cycling conditions and analysis of qPCR results were performed as above. Each SDR primer was tested against all members of the SDR family (*EpSDR-1* to *EpSDR-8*) and each one of the CYP71D clade was tested against other members of CYP71D clade encoded in gene cluster 1 and 2 (*CYP71D360, CYP71D365, CYP71D367, CYP71D369, CYP71D625* and *CYP71D627*). C_T values achieved for each gene of interest were in the range of 12 to 16 whereas C_T achieved with homologues was 28 to 38 (or not amplified at all), which means that amplification from a target sequence is a minimum 65000-fold higher than from any of the homologous sequences tested.

Phylogenetic analysis of *E. peplus* latex-specific SDRs and P450 oxidases encoded in gene cluster 1 and 2.

For initial classification, the *E. peplus* SDR cDNA – predicted amino acid sequences were compared against the comprehensive inventory of plant SDR (23) which indicated that they fell into SDR7C and SDR114C families. EpSDR-1, to EpSDR-8 sequences were searched against the non-redundant protein sequences (NR) database at the NCBI using BLASTP with the expected value lower than e-50 and search restricted to Euphorbiaceae family only. The same sequences were also searched against the SwissProt database, which contain functionally characterized proteins with the expected value lower than e-50. Finally, representative SDR114C and SDR7C family members from *A. thaliana* were also included (23). Protein sequence alignments within each of the two sets of sequences (SDR114C and SDR7C families), were made firstly with the MUSCLE algorithm (24). Conserved blocks in each alignment were evaluated and selected with Gblocks analysis (25). The best-scoring maximum-likelihood tree was inferred in conjunction with bootstrap analyses of 100 replicates using RAxML v8.2.12 (26). The trees from this first round of analyses were then evaluated. A subset of sequences was selected for a further round of analyses if (1) it contained the query sequence and (2) it formed a group with above 50% bootstrap value support. A further round of Gblocks analyses was performed to allow more residues to be included in the conserved blocks in order to achieve higher bootstrap support value in the subsequent phylogenetic analyses with RAxML. Final trees were produced with the drawing tool FigTree (Tree Figure Drawing Tool Version 1.4.2 2006-2014, Andrew Rambaut, http://tree.bio.ed.ac.uk/software/figtree/).

cDNA – predicted amino acid sequences for CYP71D365 was searched against the non-redundant protein sequences (NR) database at the NCBI using BLASTP with the expected value lower than

e-130 and search restricted to the Euphorbiaceae family and against SwissProt database, with the expected value lower than e-100. Finally, a manually curated set of sequences containing known casbene- and neocembrene oxidases from the Euphorbiaceae family as well as CYP726A and CYP71D clade members encoded in gene cluster 1 and 2 was added. All subsequent phylogenetic analysis were performed as described above.

Supplemental Figures

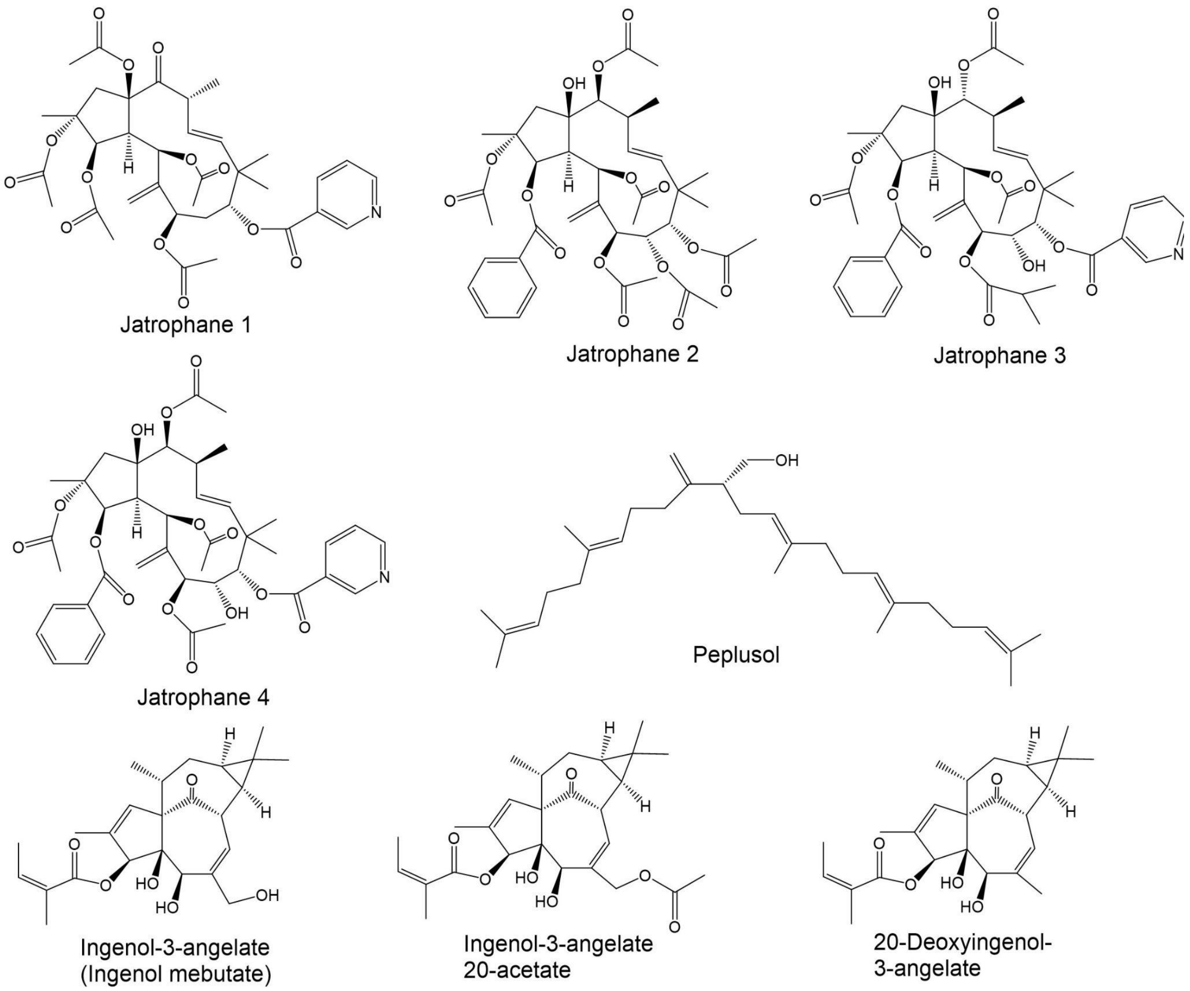

Fig. S1. Structures of the major constituents from the aerial parts of *E. peplus* as determined by NMR spectroscopy.

Compounds extracted and purified from 8-weeks old *E. peplus* plants were analysed by NMR as described in SI Materials and Methods.

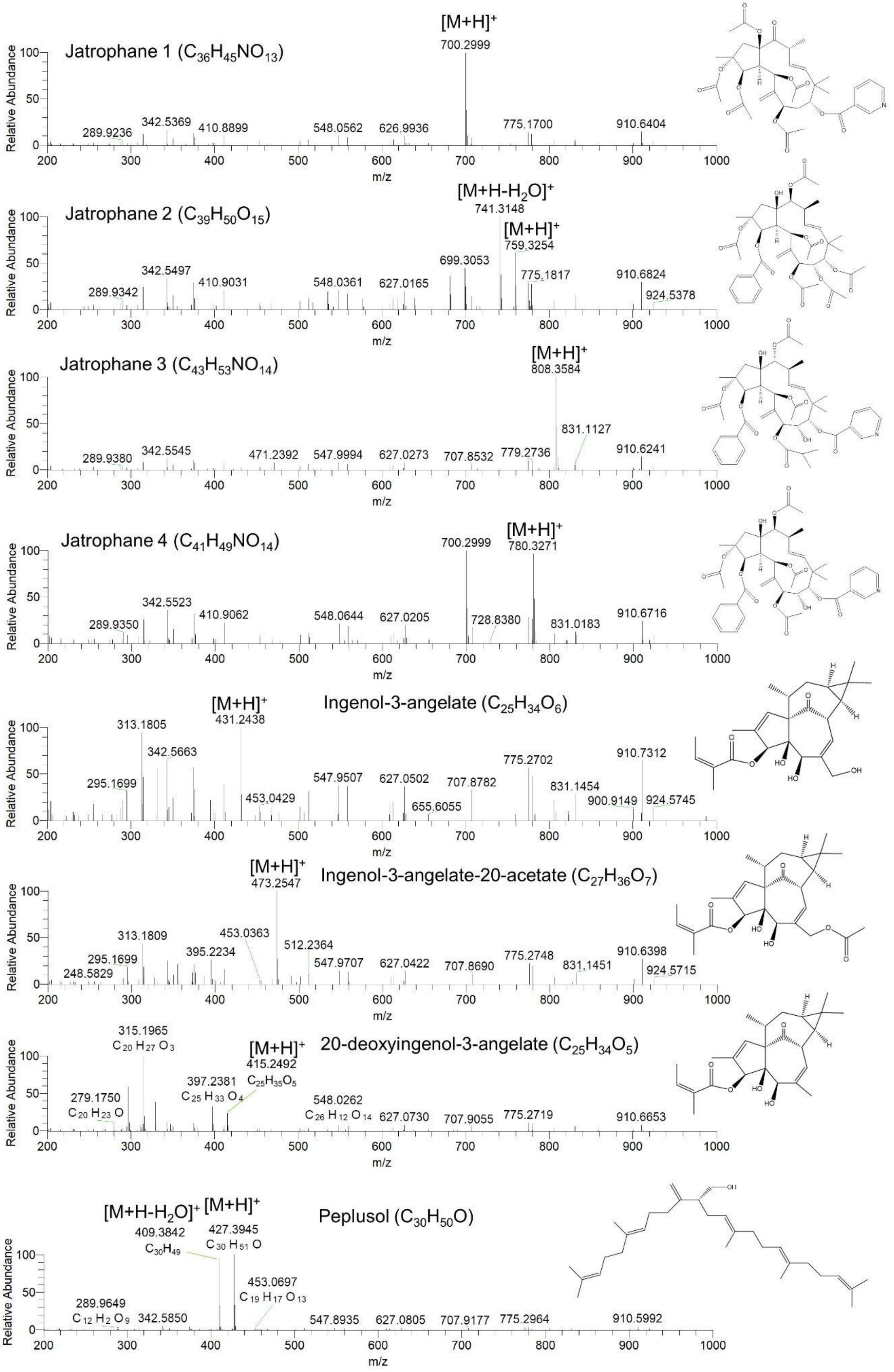

Jatrophane 1 (C36H45NO13)
[M+H]+
700.2999
289.9236
342.5369
410.8899
548.0562
626.9936
775.1700
910.6404
Jatrophane 2 (C39H50O15)
[M+H-H2O]+
741.3148
[M+H]+
759.3254
699.3053
289.9342
342.5497
410.9031
548.0361
627.0165
775.1817
910.6824
924.5378
Jatrophane 3 (C43H53NO14)
[M+H]+
808.3584
831.1127
289.9380
342.5545
471.2392
547.9994
627.0273
707.8532
779.2736
910.6241
Jatrophane 4 (C41H49NO14)
[M+H]+
700.2999
780.3271
289.9350
342.5523
410.9062
548.0644
627.0205
728.8380
831.0183
910.6716
Ingenol-3-angelate (C25H34O6)
[M+H]+
431.2438
313.1805
342.5663
295.1699
453.0429
547.9507
627.0502
655.6055
707.8782
775.2702
831.1454
900.9149
910.7312
924.5745
Ingenol-3-angelate-20-acetate (C27H36O7)
[M+H]+
473.2547
313.1809
295.1699
248.5829
395.2234
453.0363
512.2364
547.9707
627.0422
707.8690
775.2748
831.1451
910.6398
924.5715
20-deoxyingenol-3-angelate (C25H34O5)
315.1965
C20 H27 O3
[M+H]+
415.2492
C25H35O5
279.1750
C20 H23 O
397.2381
C25 H33 O4
548.0262
C26 H12 O14
627.0730
707.9055
775.2719
910.6653
Peplusol (C30H50O)
[M+H-H2O]+
409.3842
C30H49
[M+H]+
427.3945
C30 H51 O
453.0697
C19 H17 O13
289.9649
C12 H2 O9
342.5850
547.8935
627.0805
707.9177
775.2964
910.5992
Relative Abundance
m/z

Fig. S2A. Determination of molecular formulae of major constituents of *E. peplus* latex by high resolution mass spectrometry (NMR-validated structures).

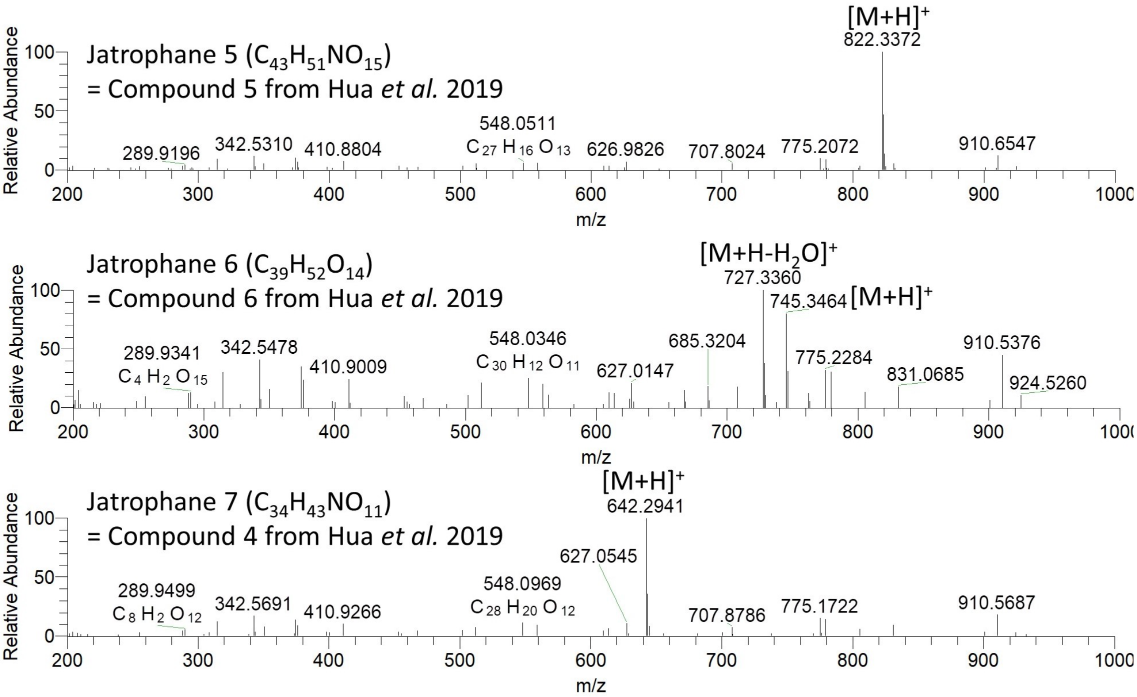

Fig. S2B. Determination of molecular formulae of major constituents of *E. peplus* latex by high resolution mass spectrometry (literature-matched *m/z* spectra).

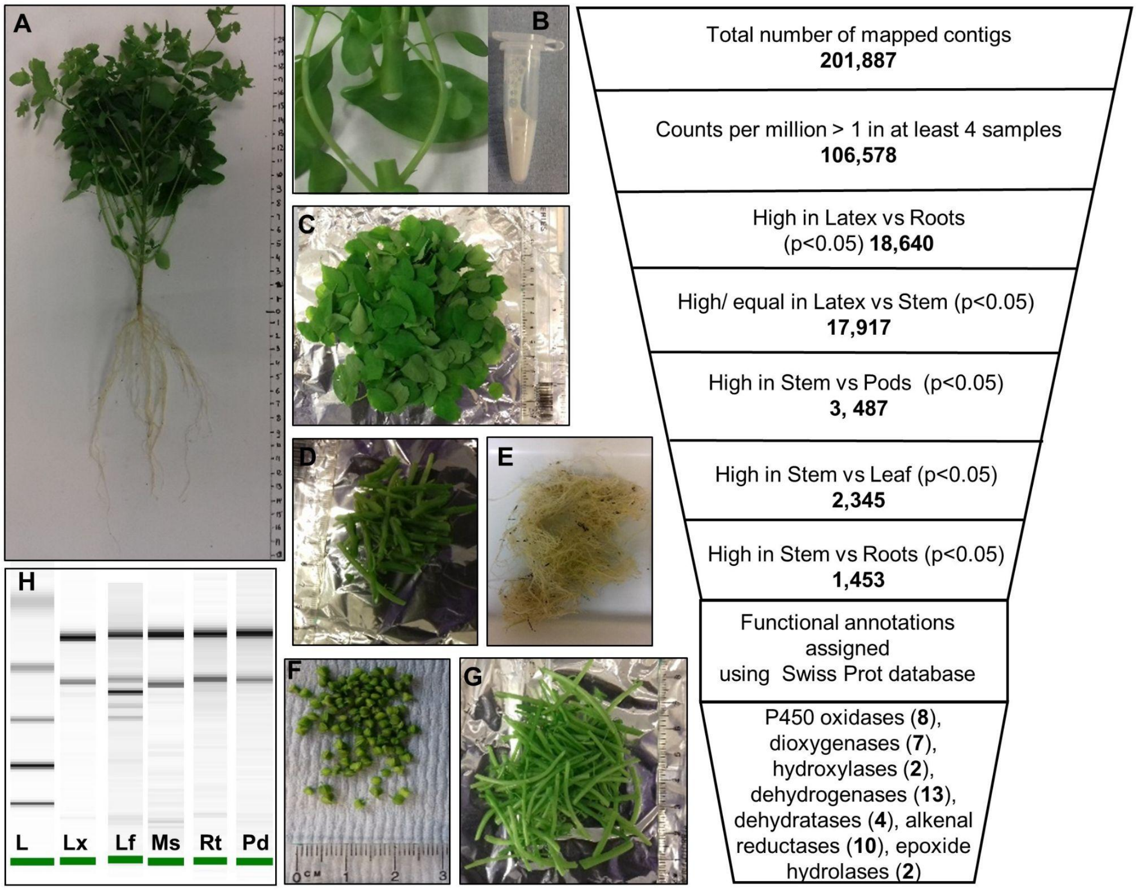

Fig. S3. RNAseq workflow for gene discovery.

RNA was extracted from five different tissues of 8-weeks old *E. peplus* plant (panel A): latex (Lx, panel B), leaves (Lf, panel C), main stems (Ms, panel D), roots (Rt, panel E), pods (Pd, panel F) and run on 2100 Bioanalyser (H). RNA ladder (L) with: 4kb, 2kb, 1kb, 0.5kb and 0.2kb bands plus a green bar for lower size (25bp) marker shown on panel H. Additional tissues used for metabolomic analysis include: side stems (panel G) and mature seeds. *De novo* assembly of raw data and subsequent bioinformatics analysis was performed as described in SI Materials and Methods. Selection process, starting from 201,887 mapped contigs and finished with 46 candidate genes is presented on the right.

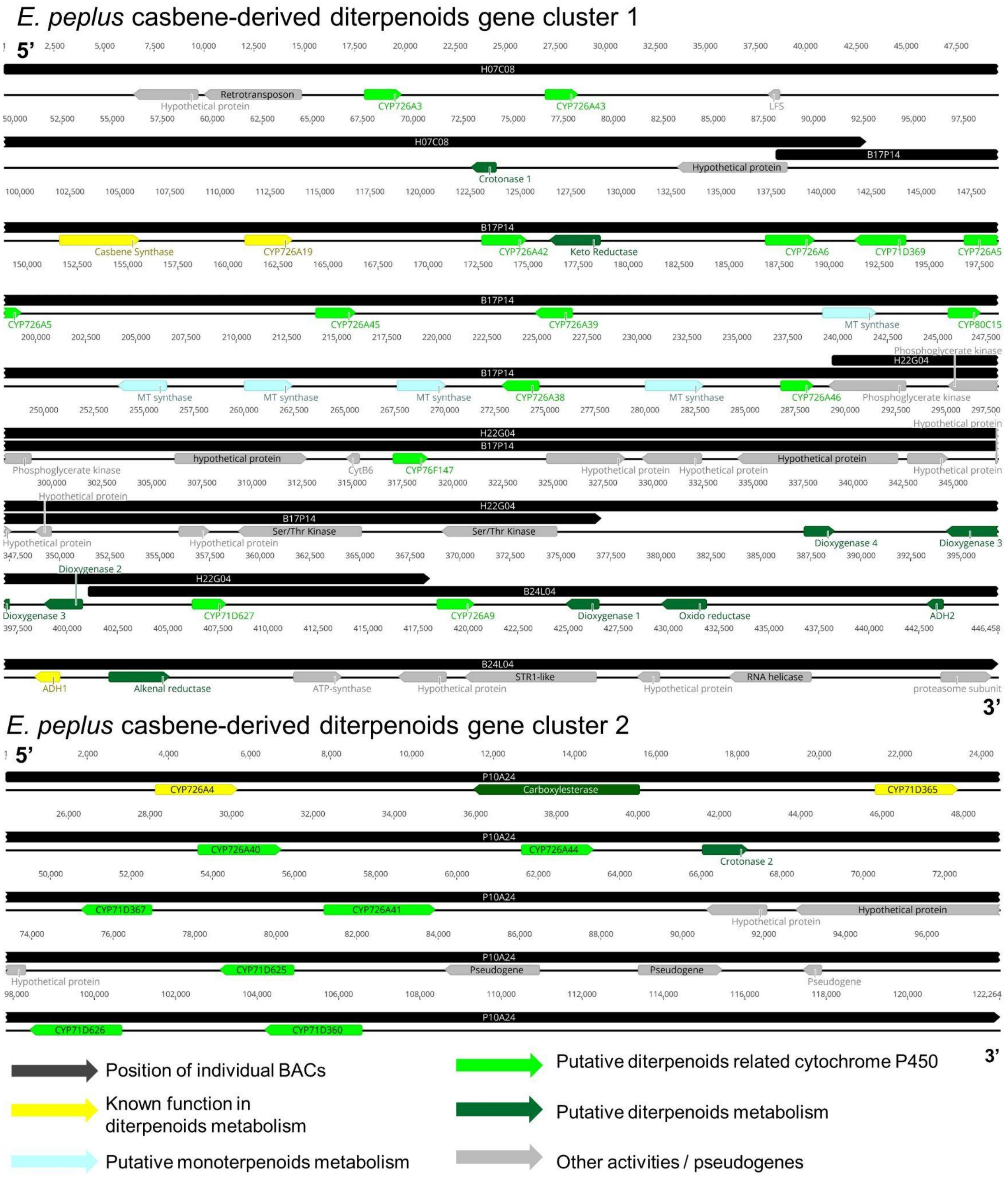

Fig. S4. Two *E. peplus* gene clusters containing functionally characterised (17, 26) diterpenoid related genes. BAC-library construction and screening using known *E. peplus* genes involved in biosynthesis of casbene derived diterpenoids was performed as described in the SI Materials and Methods. *E. peplus* casbene-derived diterpenoids gene cluster 1 encoding casbene synthase (*CAS*), casbene 5-oxidase (*CYP726A19*) and alcohol dehydrogenase (*ADH1*) and gene cluster 2 encoding casbene 5-oxidase (*CYP726A4*) and casbene-9-oxidase (*CYP71D365*) are depicted with surrounding genes color-coded according to predicted function. Abbreviations: LFS – lachrymatory factor synthase, MT synthase – putative monoterpene synthase, Ser/Thr kinase – serine/threonine kinase, STR1 – Thiosulfate/3-mercaptopyruvate sulfurtransferase 1. Numbering of base pairs shown for each gene cluster. GenBank accession numbers for the individual BACs: MW775849 (H07C08), MW775847 (B17P14), MW775846 (H22G04), MW775848 (B24L04) and MW775845 (P10A24).

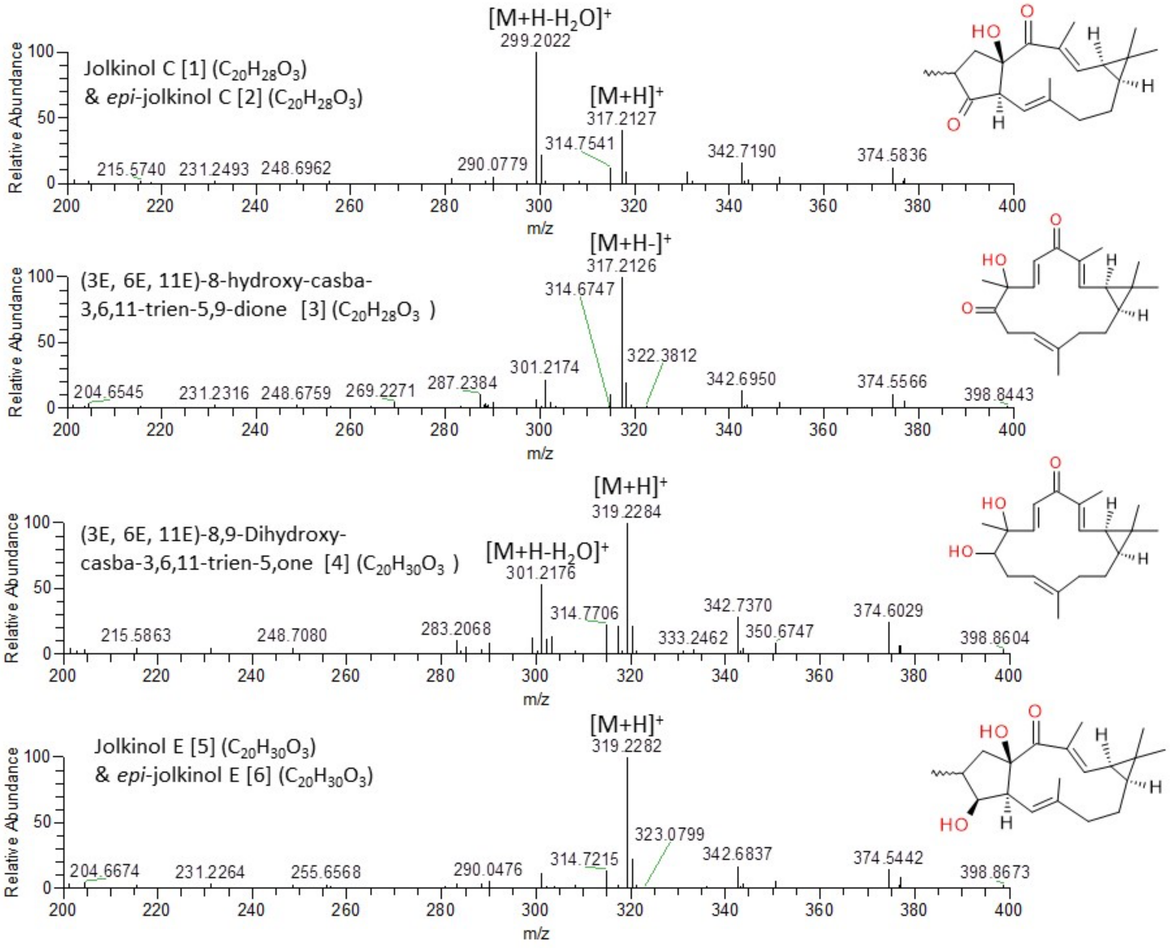

Fig. S5. Determination of molecular formulae of casbene derived diterpenoids *from E. peplus* by high resolution mass spectrometry.

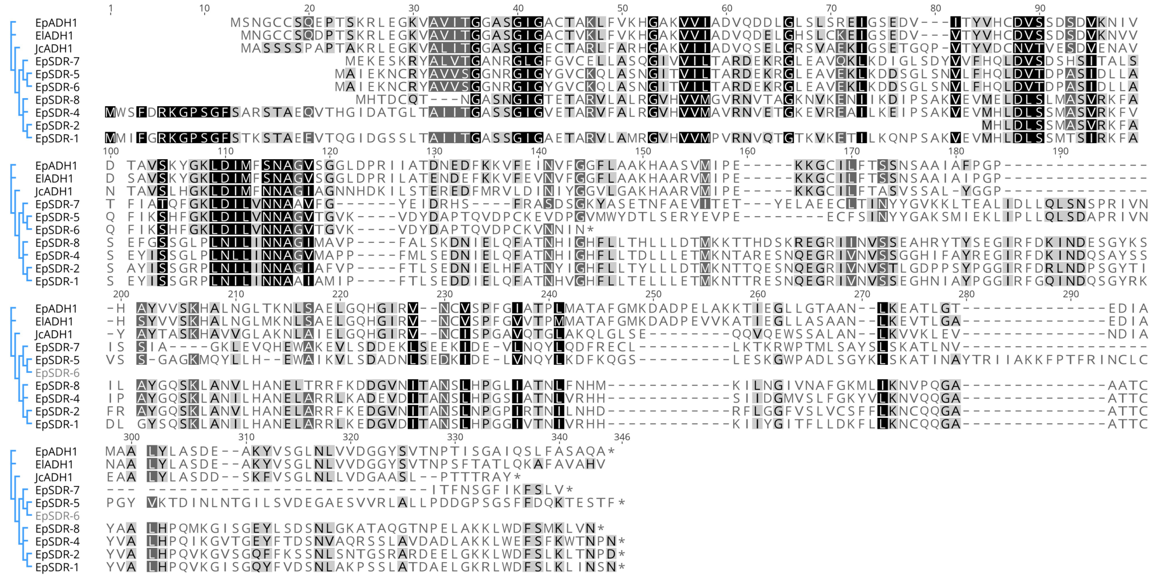

Fig. S6. Protein alignment of latex-specific *E. peplus* SDR family members and alcohol dehydrogenases involved in jolkinol C biosynthesis.

cDNA – predicted amino acid sequences for alcohol dehydrogenases involved in jolkinol C biosynthesis: *JcADH1* (XM_012211448), *ElADH1* (KR350665) and *EpADH1* (KR350671) and six *SDR* gene family members: *EpSDR-1* (MW594428), *EpSDR-2* (MW594429), *EpSDR4* (MW594430), *Ep-SDR-5* (MW594431), *EpSDR-6* (MW594432), *EpSDR-7* (MW594433) and *EpSDR-8* (MW594434) were aligned using MUSCLE alignment tool (2). Consensus tree was constructed by Neighbor-Joining method and with bootstrap analyses of 100 replicates using Geneious Tree Builder (Geneious Prime® 2021.2.2). Shading indicates the level of protein sequence similarity.

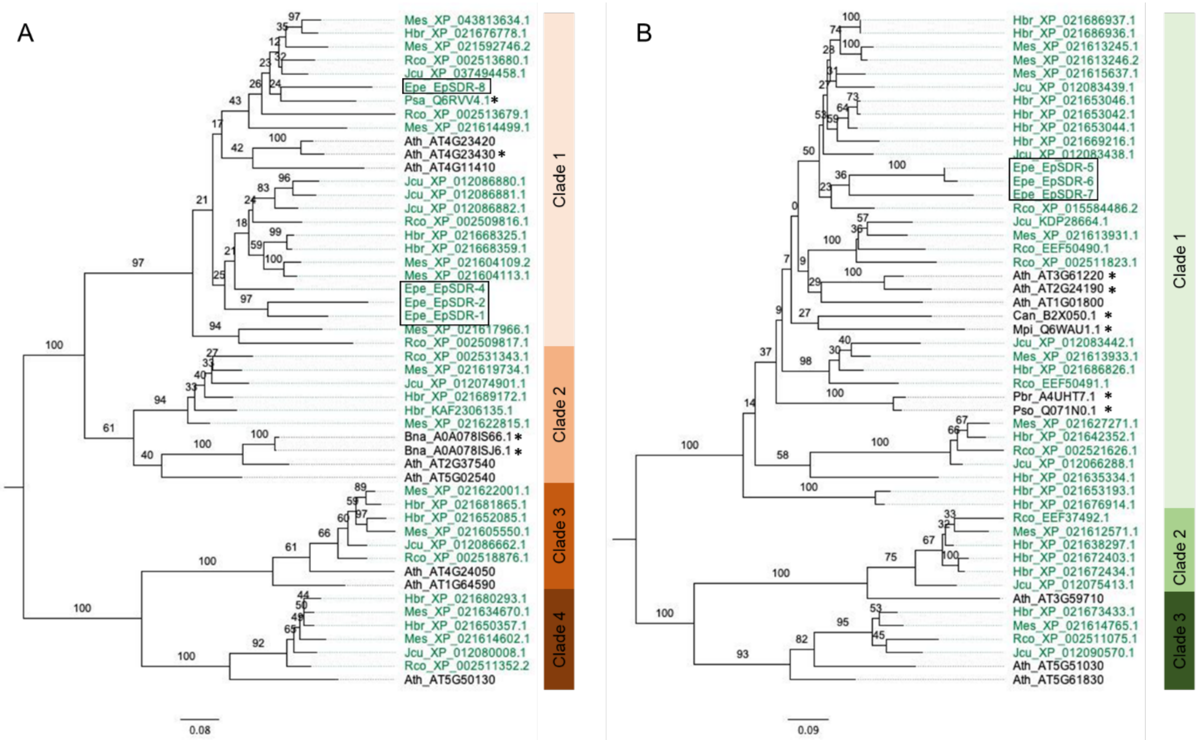

Fig. S7. Phylogenetic analysis of representative short chain reductases/dehydrogenases of the SDR7C (A) and SDR114C (B) families.

The set of cDNA – predicted amino acid sequences for members of SDR7C (A) and SDR114C (B) families from: Euphorbiaceae (green), Arabidopsis and other functionally characterized members (asterisk) was selected as described in the materials and methods. Amino acid sequences were aligned using MUSCLE alignment tool (18), Conserved blocks in each alignment were evaluated and selected with Gblocks analysis (19).The best-scoring maximum-likelihood tree was inferred in conjunction with bootstrap analyses of 100 replicates using RAxML v8.2.12 (20). The tree was visualized with the drawing tool FigTree (Tree Figure Drawing Tool Version 1.4.2 2006-2014, Andrew Rambaut, http://tree.bio.ed.ac.uk/software/figtree/). Species abbreviations: Epe (*Euphorbia peplus*), Mes (*Manihot esculenta*), Hbr (*Hevea brasiliensis*), Rco (*Ricinus communis*), Jcu (*Jatropha curcas*), Ath (*Arabidopsis thaliana*), Bna (*Brassica napus*), Psa (Pisum sativum), Can (*Capsicum annuum*), Mpi (*Mentha x piperita*), Pbr (*Papaver bracteatum*), Pso (*Papaver somniferum*). Functionally characterized proteins from SDR7C family include: Short-chain dehydrogenases TIC 32 (parts of chloroplast protein import translocon complex) from: *Pisum sativum* (Psa_Q6RVV4.1), *Arabidopsis thaliana* (Ath_ AT4G23430) and *Brassica napus* (Bna_A0A078ISJ6.1 and Bna_A0A078IS66.1). Functionally characterized proteins from SDR114C family include: salutaridine reductases from *Papaver bracteatum* (Pbr_A4UHT7.1) and *Papaver somniferum* (Pso_Q071N0.1); (+)-neomenthol dehydrogenases from *Arabidopsis thaliana* (Ath_AT3G61220, Ath_AT2G24190), *Capsicum annuum* (Can_B2X050.1) and (-)-isopiperitenone reductase from *Mentha x piperita* (Mpi_Q6WAU1.1). Members of latex-specific *E. peplus* SDRs are highlighted in the boxes. The scale bar indicates the number of substitutions per variant site and numbers at the nodes indicate bootstrap support (%).

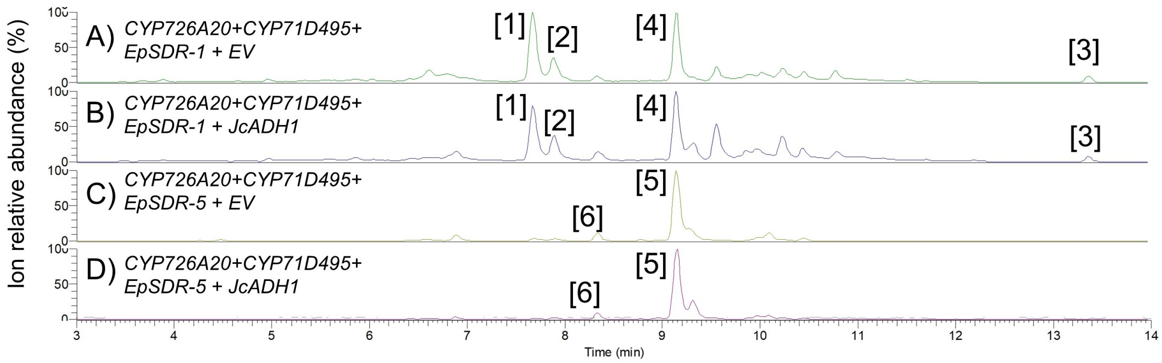

Fig. S8. Evaluation of the effect of *JcADH1* on the production of casbene-derived diterpenoids in *N. benthamiana*. Four-week-old *N. benthamiana* plants infiltrated with MEP-pathway genes (*AtDXS, AtHDR, AtGGPPs)*, casbene synthase (*JcCAS*), and casbene oxidases involved in production of jolkinol C [1] and *epi*-jolkinol C [2] (*CYP726A20* and *CYP71D495*) combined with: **(A,B)** *EpSDR-1* producing: (3E, 6E, 11E)-8,9-dihydroxy-casba-3,6,11-trien-5-one [4;] **(C,D)** *EpSDR-5* producing jolkinol E [5] and *epi*-jolkinol C [6]; Infiltrated leaves were extracted and analysed by LC-MS (SI Materials and Methods) and raw chromatograms for m/z 317-320 are shown. Labelling of the peaks is consistent with Figure 3 and Figure 4.

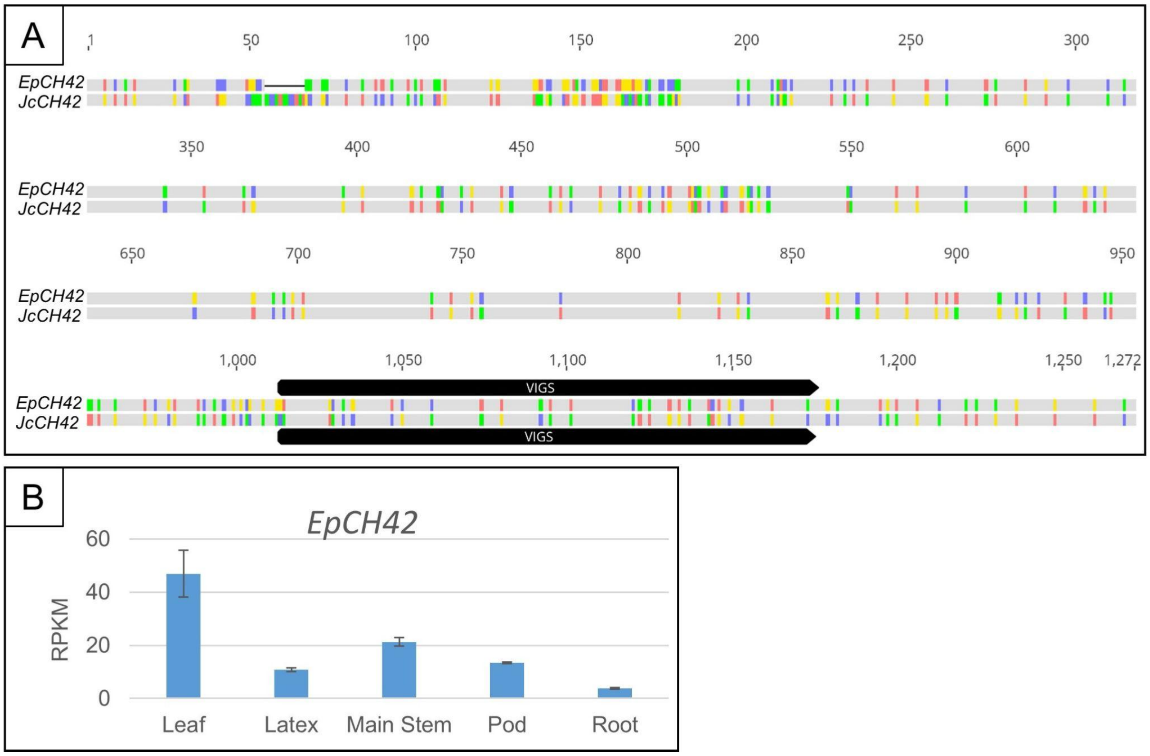

Fig. S9. (A) Nucleotide sequence alignment between *J. curcas Chlorata 42* (*JcCH42,* KDP21059) and the *E. peplus* homologue (*EpCH42,* OL744077*)* Sequence non-alignments highlighted in colors. Sequence fragments targeted by VIGS shown as black bar. **(B) Expression profile of the *EpCH42* VIGS-marker gene across five tissues analysed by RNAseq.** RPKM values calculated for each of the tissue as described in SI Materials and Methods. Error bars – SEM (n=4).

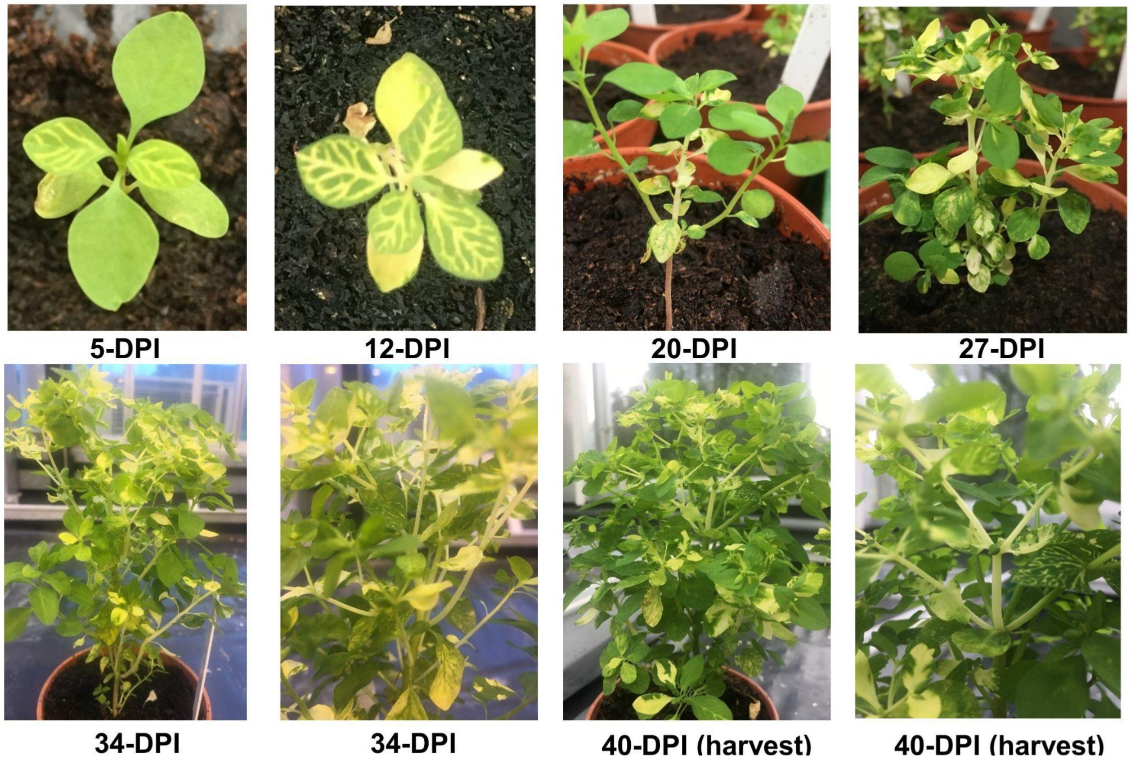

Fig. S10. Progression of chlorosis during development of leaves and stems of *E. peplus* infiltrated at the 9 day old seedling stage with pTRV2-*EpCH42*-VIGS construct. DPI - Days Post Infiltration.

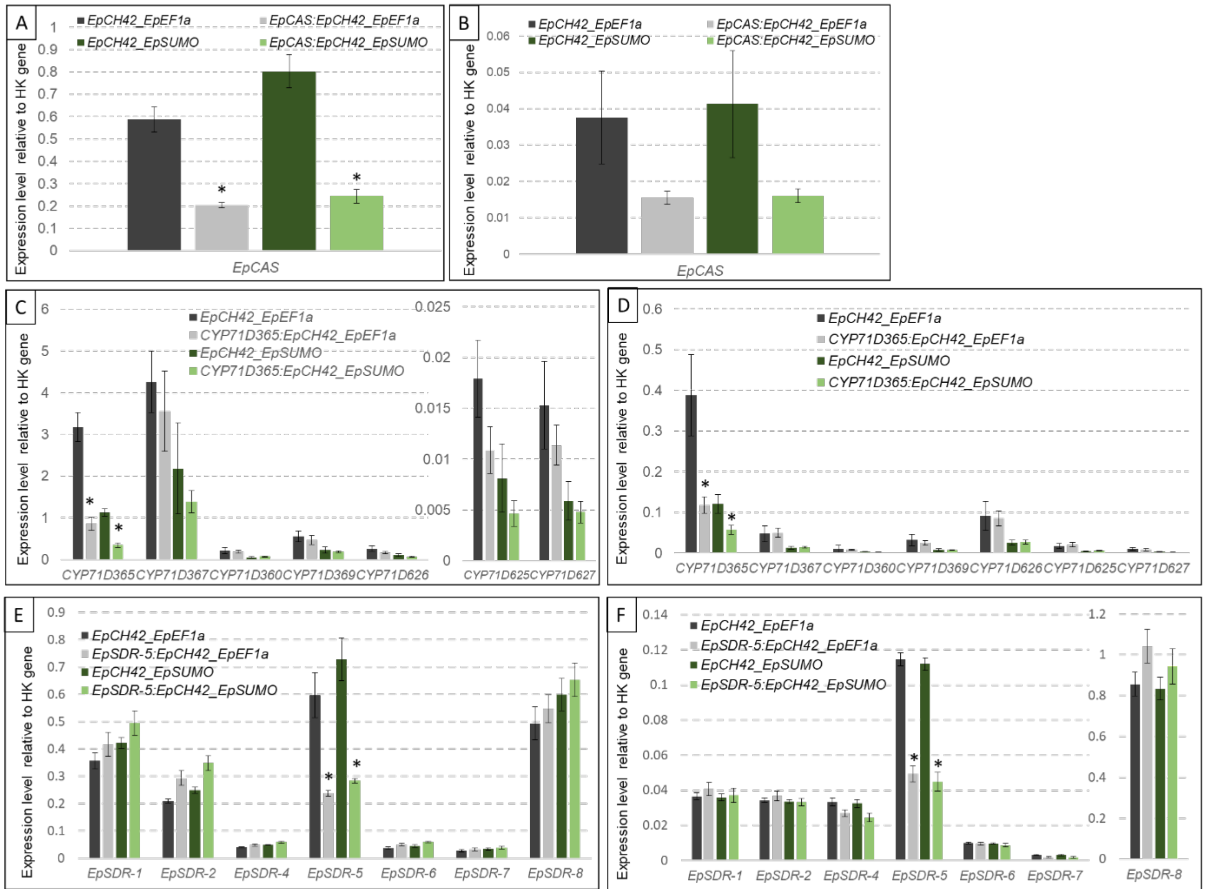

Fig. S11. Transcript abundance of diterpenoid-related genes subjected to VIGS and their closest homologues in *E. peplus* leaves and stems.
Expression levels of the three VIGS-targeted genes and their closest homologues, relative to two selected housekeeping genes: EpEF1a and EpSUMO were measured for stem (A, C and E) and leaf (B, D and F) tissues for VIGS marker-only (EpCH42, dark-grey and dark-green bars) and marker plus diterpenoid pathway genes (light-grey and light green bars). Error bars – SEM (n=5). Statistically significant (T-test) changes between control (EpCH42) and diterpenoid pathway silenced genes are indicated by asterisks (*-p-value <0.01). VIGS, RNA extractions, cDNA synthesis and qPCR analysis were performed as described in SI Materials and Methods.

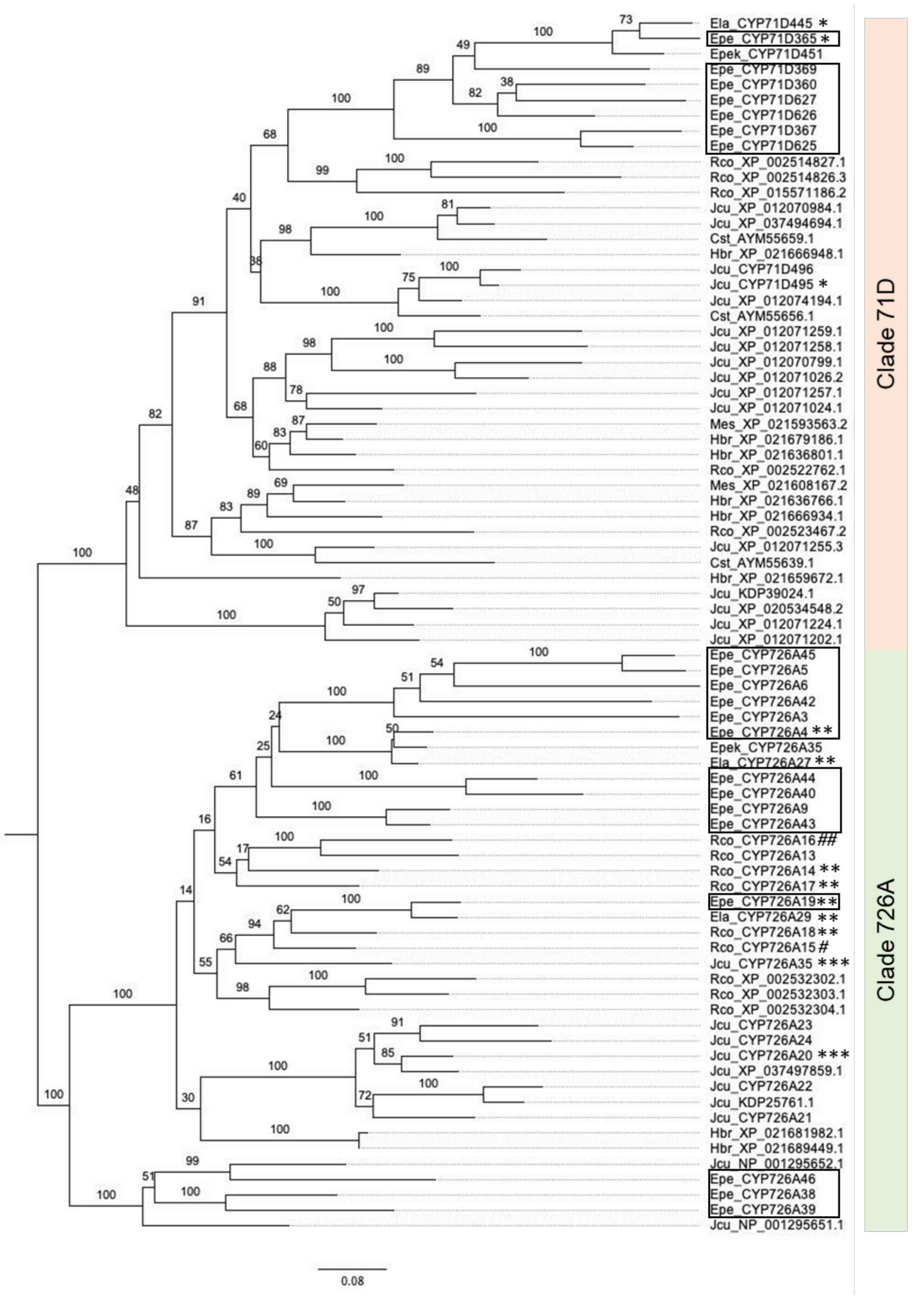

Fig. S12. Phylogenetic analysis of representative Cytochrome P450 oxidases from Euphorbiaceae CYP71D and CYP726 clades. The set of cDNA – predicted amino acid sequences for CYP726A and CYP71D clades of P450 oxidases encoded in gene clusters 1 and 2 (boxed)and their homologues from Euphorbiaceae were selected as described in the materials and methods. Amino acid sequences were aligned using MUSCLE alignment tool (18), Conserved blocks in each alignment were evaluated and selected with Gblocks analysis (19).The best-scoring maximum-likelihood tree was inferred in conjunction with bootstrap analyses of 100 replicates using RAxML v8.2.12 (20). The tree was visualized with the drawing tool FigTree (Tree Figure Drawing Tool Version 1.4.2 2006-2014, Andrew Rambaut, http://tree.bio.ed.ac.uk/software/figtree/). Species abbreviations: Ele (*Euphorbia lathyris*), Epe (*Euphorbia peplus*), Epek (*Euphorbia pekiniensis*), Cst (*Croton stellatopilosus*), Mes (*Manihot esculenta*), Hbr (*Hevea brasiliensis*), Rco (*Ricinus communis*), Jcu (*Jatropha curcas*), functionally characterized oxdiases lebelled on the reee as: * *casbene-9-oxidase, ** casbene-5-oxidase, *** casbene-5,6-oxidase, ## 5-ketocasbene 7,8-epoxidase and # neocembrane-5-oxidase*

Supplemental Tables

Table S1. Levels of selected di- and triterpene metabolites in various tissues of *Euphorbia peplus.*

Table S2. Levels of selected di- and triterpene metabolites in marker-only (*EpCH42*) and marker plus *E. peplus CAsbene Synthase* silenced (*EpCH42:EpCAS)* stems and leaves.

Table S3. Levels of selected di- and triterpene metabolites in marker-only (*EpCH42*) and marker plus *E. peplus casbene-9-oxidase* silenced (*EpCH42:CYP71D365)* stems and leaves.

Table S4. Levels of selected di- and triterpene metabolites in marker-only (*EpCH42*) and marker plus *E. peplus Jolkinol C 3-keto-rductase* silenced (*EpCH42:EpSDR-5)* stems and leaves.

Table S5. Primers used for screening the *E. peplus* BAC library and for BAC sequencing

**Target seq ID**	**Forward (5'--3')**	**Reverse (5'--3')**	**Purpose**
EpCAS_BAC	GCCAAGTTGGATTTCAACCGAGTGC	TTGCGGAGTACGCCGGAGAT	Screening with *EpCAS*
EpA19_BAC	ACTCGAAAGCAGGAAAGCCCGT	CGCAAACTCTGGAGGCCCGAC	Screening with *EpCYP726A19*
H07C08_BAC	TCTTCAGATATGTCGAGGTTGG	CATCCGCAACTTGATTTTCA	Screening with *BACH07C08*
EpA4_BAC	GGGAATCTTCAAGTTCCATTGACTAATG	CGGACTTACGAGTGAGCCAAAAC	Screening with *EpCYP726A4*
BEUP_17P04_L	CCGTGAATGGGTCGAAGATAA	AAGCGTTCAAACTCAAAGGAAC	flanking primers for screening
BEUP_17P04_R	GGGTTCCATCATCCAACACTAA	GCTCTCCATCTGCCGATAAAT	flanking primers for screening
BEUP_21L04_L	TTTATGGAGCCTTACACACAAAAGGT	CCCCACAATAATTGGAGGCCCA	flanking primers for screening
BEUP_21L04_R	GCTGGAGCAGAGCGAATAA	CTCCCTGCACGAATTCTACTT	flanking primers for screening
HEUP_07H07_L	AGTTATGGGACGGAGGGAGTATCA	TCGGAAGTACGTGCTCTCTTCA	flanking primers for screening
HEUP_07H07_R	TCACCAATGTATGAGTGAAGAGTCCA	GCCGAAAGGAAATGGAAGGAACG	flanking primers for screening
HEUP_17G09_L	AGTCGGGTGGACGTAGCTCT	TTGACGTTTGGGAGGTCAAAACA	flanking primers for screening
HEUP_17G09_R	TGCGATTGTTTCTTCACCGCT	AGGTGTCGGGTCAAGAGGCT	flanking primers for screening
HEUP_19A07_L	AGCTTGTCTAAAATGCTATTGGGCT	TCCGAGTTGAACCAGCATGGA	flanking primers for screening
HEUP_19A07_R	ACTTGTTGCTTAAACCTCATCCCACA	ACCGAAGCGTCATTCGTGTCA	flanking primers for screening
B17P04_L	GTGGTTCGTTCTCGACAGTT	ATGTAGGGCCCATTATCAATCC	flanking primers for screening
B17P04_R	GATCCATCCTCCACGAGAAAC	GCTCTCCATCTGCCGATAAAT	flanking primers for screening
B21L04_L	CTCGCAAGAGTTCAAGCTTTATT	ATAATTGGAGGCCCAAGAGG	flanking primers for screening
B21L04_R	CCGTGTGAGAAGCTGTTACT	GCCGATTCCGTCCCTTATT	flanking primers for screening
H07H07_L	TTATGGGACGGAGGGAGTAT	GAGGAGTGGATAGCTGAGAAAG	flanking primers for screening
H07H07_R	GACAGTAAGTTTGTTCGGTGATTG	GAAGGAACGATGTGACTGACTAC	flanking primers for screening
H17G09_L	TACTATAGTCGGGTGGACGTAG	GCTTATTGAGTCTGATCCTCTCTT	flanking primers for screening
H17G09_R	GCTAGTCCATCGTACTCACAAA	GGGTCAAGAGGCTAAGGATATG	flanking primers for screening
BACSEQ	GTTGGGTAACGCCAGGGTTT	TGTGTGGAATTGTGAGCGGA	BAC sequencing

Table S6. Primers used for cloning and sub cloning of *E. peplus* candidate gene cDNAs and corresponding GenBank accession numbers

**Gene ID**	**GenBank Accession**	**SwissProt annotation**	**Forward (5'--3')**	**Reverse (5'--3')**
EpSDR-1	MW594428	Short-chain dehydrogenase TIC 32, chloroplastic	CTGTATATTCTGCCCAAATTCGC GAAAAAATGATGATTTTTGGGAG GAAAGGAC	AATTTAATGAAACCAGAGTTAAA GGCTAGTTGGAATTAATCAATTT CAAGCTGAAATC

EpSDR-2	MW594429	Short-chain dehydrogenase TIC 32, chloroplastic	CTGTATATTCTGCCCAAATTCGCGAAAAAATGCACTTAGATCTCAGCTC	ATTTAATGAAACCAGAGTTAAAGGCTAGTCGGGATTAGTCAATTTGAG
EpSDR-4	MW594430	Short-chain dehydrogenase TIC 32, chloroplastic	CTGTATATTCTGCCCAAATTCGCGAAAAAATGTGGAGTTTTGATAGGAAAGGGC	AATTTAATGAAACCAGAGTTAAAGGCTAGTTCGGATTGGTCCATTTG
EpSDR-5	MW594431	(+)-neomenthol dehydrogenase	CTGTATATTCTGCCCAAATTCGCGAAAAAATGGCGATAGAGAAGAATTGC	AATTTAATGAAACCAGAGTTAAAGGTCAAAATGTTGATTCAGTCTTTTGATC
EpSDR-6	MW594432	(+)-neomenthol dehydrogenase	CTGTATATTCTGCCCAAATTCGCGAAAAAATGGCGATAGAGAAGAATTGC	AATTTAATGAAACCAGAGTTAAAGGTTAATTAATGTTGTTTACCTTACAAGGATC
EpSDR-7	MW594433	(+)-neomenthol dehydrogenase	CTGTATATTCTGCCCAAATTCGCGAAAAAATGGAGAAAGAGAGCAAGAGGTA	AATTTAATGAAACCAGAGTTAAAGGTGATAACACTATCAAAAGCAACTCTCAATG
EpSDR-8	MW594434	Short-chain dehydrogenase TIC 32, chloroplastic	CTGTATATTCTGCCCAAATTCGCGAAAAAATGCATACTGATTGTCAAACAAAC	AATTTAATGAAACCAGAGTTAAAGGTCAGTTAACCAGTTTCATGCT
CYP71D360	MW594404	CytP450 oxidase	CTGTATATTCTGCCCAAATTCGCGAAAAAATGGAATCCCATTTTGCCTCCTCAGA	AATTTAATGAAACCAGAGTTAAAGGTTAACAAGGAGGATTGTATAGAGTCGGAATG
CYP71D365	MW594405	CytP450 oxidase	CTGTATATTCTGCCCAAATTCGCGAAAAAATGGAGTTAGAACTTCACCTCCCTTGTTC	AATTTAATGAAACCAGAGTTAAAGGCTAGGAAGGAACATATGGAGTAGGAATAAT
CYP71D367	MW594406	CytP450 oxidase	CTGTATATTCTGCCCAAATTCGCGAAAAAATGGAGTTCTTCTTTTCCAAATTAATTCCTTC	AATTTAATGAAACCAGAGTTAAAGGCTAAGGAGTAGGAGGAACATATGGAACA
CYP71D369	MW594407	CytP450 oxidase	CTGTATATTCTGCCCAAATTCGCGAAAAAATGTCGATAGATTTTCAATTTCCATCTTTAG	AATTTAATGAAACCAGAGTTAAAGGTTAGTAATTGGATGAAGTTGTGGGAAG
CYP71D625	MW594408	CytP450 oxidase	CTGTATATTCTGCCCAAATTCGCGAAAAAATGGATCCCCAGATTATTCCTTCAAACTGG	AATTTAATGAAACCAGAGTTAAAGGCTAAATTTTTCCTTGGATAGAAGGACGATATG
CYP71D626	MW594409	CytP450 oxidase	CTGTATATTCTGCCCAAATTCGCGAAAAAATGATGGAATCCCTTTTTTCCTCCACAGAATG	AATTTAATGAAACCAGAGTTAAAGGTTAACCGATGGGAGGATAATATGGTGTG
CYP726A19 [a]	MW594410	CytP450 oxidase	AAAACCGGTAAAAATGGCAACACTTCAACATTCAATGC	AAAACTCGAGTCAGTTTGCAGGTGAAGTATGGAATGG
CYP726A3	MW594418	CytP450 oxidase	CTGTATATTCTGCCCAAATTCGCGAAAAAATGGATCTTGAAATGCCCTCTTTTC	AATTTAATGAAACCAGAGTTAAAGGTCAAGCAATTTGATGGGGGGAG
CYP726A38	MW594413	CytP450 oxidase	CTGTATATTCTGCCCAAATTCGCGAAAAAATGGAACTAAATATTATTTCATTCTCATTTCTTCC	AATTTAATGAAACCAGAGTTAAAGGTTAATTTAAAAGTCTAAGGATGGGATCGTATG
CYP726A39	MW594419	CytP450 oxidase	CTGTATATTCTGCCCAAATTCGCGAAAAAATGGAAATCCAAATGATTCATTTTTCGTTTCTC	AATTTAATGAAACCAGAGTTAAAGGTCAATTGGGACGAAATGAAGTAGGAATC
CYP726A4	MW594420	CytP450 oxidase	CTGTATATTCTGCCCAAATTCGCGAAAAAATGGAGCTTCAATTTCAAATCCC	AATTTAATGAAACCAGAGTTAAAGGTTAACTTAATGTGTATGGAATAGGAATCAAC
CYP726A41	MW594414	CytP450 oxidase	CTGTATATTCTGCCCAAATTCGCGAAAAAATGCGTCATACTATAAACATATATGTAAAGTAC	AATTTAATGAAACCAGAGTTAAAGGTCACGACTTCCTTGAGTCTAAGCTAGC

CYP726A43	MW594421	CytP450 oxidase	CTGTATATTCTGCCCAAATTCGCGAAAAAATGGAACTTGTCCAAATCCCCATCTTC	AATTTAATGAAACCAGAGTTAAAGGTCAAAGAAAATGTGGGATGGGAATAAGTTC
CYP726A44	MW594422	CytP450 oxidase	CTGTATATTCTGCCCAAATTCGCGAAAAAATGGAGCAGCAAAATCTCCCTTTCCC	AATTTAATGAAACCAGAGTTAAAGGTCACTAGATACATACTTGATATGGAATAGGAATC
CYP726A45	MW594423	CytP450 oxidase	CTGTATATTCTGCCCAAATTCGCGAAAAAATGGAGCTTCAAATCCCCTCTTTCC	AATTTAATGAAACCAGAGTTAAAGGTTAACTTTTAGAAAATGGAATAGGAATCAAGC
CYP726A5	MW594424	CytP450 oxidase	CTGTATATTCTGCCCAAATTCGCGAAAAAATGGAGTTCACTTTATCACTTAAAAAAATG	AATTTAATGAAACCAGAGTTAAAGG TCAAATTTTAGAAAACGGAATTGGAATC
CYP726A6	MW594425	CytP450 oxidase	CTGTATATTCTGCCCAAATTCGCGAAAAAATGAAAATGCTTGAGCAAATTCC	AATTTAATGAAACCAGAGTTAAAGGTTAATTCTTTGGCAATGAAGAAGATG
CYP726A9	MW594426	CytP450 oxidase	CTGTATATTCTGCCCAAATTCGCGAAAAAATGGAGTTCATCAATCGGCAATTAATTAGTG	AATTTAATGAAACCAGAGTTAAAGGTTAATTTGCAGACAAAGGGAAATGGGG
CYP80C15	MW594427	CytP450 oxidase	CTGTATATTCTGCCCAAATTCGCGAAAAAATGGATCAAACTCTAACCCCA	AATTTAATGAAACCAGAGTTAAAGGTTAGAGCTTTCTTTTAGGAATAATATA

[a] Primers for gene *CYP726A19* contain *AgeI*-F and *XhoI*-R restriction enzyme sites and these were used to produce a fragment from cDNA which was cloned by a T4-ligase method rather than the Infusion method as described in section 1.7: *Euphorbia peplus* candidate gene cloning and transient gene expression in *Nicotiana benthamiana*.

Table S7. Primers used 5‘and 3‘RACE for *E. peplus* candidate genes.

Target seq ID	Primer (5'--3')	Purpose
EpSDR-2_GSP1	GATTAGTCAATTTGAG	5‘ RACE primer 1 (cDNA synthesis)
EpSDR-2_GSP2	CTTGAAAAATTGGCCACTTACTC	5‘ RACE primer 2 (cDNA amplification)
EpSDR-2_GSP3	CCTTAACTTGTGGATGCAATGC	5‘ RACE primer 2 (cDNA amplification, inner primer)
EpSDR-8_GSP1	CGAGCGGTGTCCATGTAGTTATG	3‘ RACE primer 1 (cDNA amplification)
EpSDR-8_GSP2	CGACCAAAGTTGAAGTCATGGAATTG	3‘ RACE primer 2 (cDNA amplification, inner primer)
AP	GGCCACGCGTCGACTAGTACTTTTTTTTTTTTTTTTT	3‘ RACE Adapter primer (cDNA syntehsis)
AAP	GGCCACGCGTCGACTAGTACGGGIIGGGIIGGGIIG	5'RACE Abridged Anchor Primer (cDNA amplification)
AUAP	GGCCACGCGTCGACTAGTAC	Abridged Universal Amplification Primer (cDNA amplification)

Table S8. Primers used for creating jolkinol C producing yeast strains and for heterologous expression of EpSDR-1 and EpSDR-5.

Primer name	Primer sequence (5' to 3')	Purpose
F_BamHI-tHMG1	GAGAGGATCCATGGACCAATTGGTGAAAACTGAA	Forward primer to amplify tHMG1 and clone into pBEVY-L
R_PstI-tHMG1	GAGACTGCAGTTAGGATTTAATGCAGGTGACGG	Reverse primer to amplify tHMG1 and clone into pBEVY-L
F_ALD6-BamHI	GAGAGGATCCATGACTAAGCTACACTTTGAC	Forward primer to amplify ALD6 and clone into pBEVY-L
R_ALD6-SalI	GAGAGTCGACTTACAACTTAATTCTGACAGC	Reverse primer to amplify ALD6 and clone into pBEVY-L
F_ARS 308a repair	ATAAGTGAAATTTCAACATTAACTTCGAATTTTTTTCTTTTTATCTAACAAGATTACTCTAACGCCTCAGCCATC	Forward primer with 50 bp homoolgy to ARS308a locus to amplify genecassette from pBEVY-L for integration

R_ARS 308a repair	CTATTGAAACTATTGTGTAATAGAAGTGGTAGCAATATG TAGCAAAGAAGCAATTCGAGCTATTACCGATGATGG	Reverse primer with 50 bp homoolgy to ARS308a locus to amplify genecassette from pBEVY-L for integration
F_ARS 1021b repair	TCCATGACTCAAATTTTCCAGTGTCTCTTAGCAGTTAAA CCATTCCTGCCAGATTACTCTAACGCCTCAGCCATC	Forward primer with 50 bp homoolgy to ARS1021b locus to amplify genecassette from pBEVY-L for integration
R_ARS 1021b repair	CACGTGATATTTCACAGAATGAATTTCATCACGTGCGTA TTATCTCTTAACAATTCGAGCTATTACCGATGATGG	Reverse primer with 50 bp homoolgy to ARS1021b locus to amplify genecassette from pBEVY-L for integration
F_ARS 911b repairC	AGAAAAAAATCGGATGTTGAATGGGCATAAATATAAATG TATATATAAGTGAAACAGCTATGACCATGATTAC	Forward primer with 50 bp homoolgy to ARS911b locus to amplify genecassette from pBEVY-L for integration
R_ARS 911b repairC	CATTTCTGTTTTAAATTTTATGTCTGTTTTGTATGCTATTT CATTTTTCAGCTCGAGGGCATGCGAAG	Reverse primer with 50 bp homoolgy to ARS911b locus to amplify genecassette from pBEVY-L for integration

Table S9. Primers used for cloning and creating VIGS constructs and for qPCR

**Target seq ID**	**GenBank Accession**	**Primer (5'--3')**	**Purpose**
EpCH42_VIGS_F	OL744077	CTTAGATTCTGTGAGTAAGGTTACCGGG ATCCGAGTGCAGATAGATCGCG	Forward primer with EcoRI InFusion tail for cloning into pTRV2 vector
EpCH42_VIGS_R	OL744077	CTCGAGACGCGTGAGCTCGGTACCGGG ATCCGATAACAGTAGCAATATCCTCTG	Reverse primer with BamHI InFusion tail for cloning into pTRV2 vector
EpCAS_VIGS_F	KC702397	CGGTACCGAGCTCACGCGTCATCCGAC TTGTCAATGACC	Forward primer with XhoI tail for cloning into pTRV2 vector
EpCAS_VIGS_R	KC702397	TTTAATGTCTTCGGGACATGCCCTTTCCA ACAGTCTCTTGCC	Reverse primer with SmaI tail for cloning into pTRV2 vector
CYP71D365_VIGS_F	MW594405	CGGTACCGAGCTCACGCGTCTAGAGAA AGCTGTGTGATTGG	Forward primer with XhoI tail for cloning into pTRV2 vector
CYP71D365_VIGS_R	MW594405	TTTAATGTCTTCGGGACATGCCCACTAT CAAGAAATCTCTCTGGTC	Reverse primer with SmaI tail for cloning into pTRV2 vector
EpSDR-5_VIGS_F	MW594431	CGGTACCGAGCTCACGCGTCGCCGATC TTTCTGGTTATAAACTC	Forward primer with XhoI tail for cloning into pTRV2 vector
EpSDR-5_VIGS_R	MW594431	TTTAATGTCTTCGGGACATGCCCCCGAC AATATTCCCGTGTTG	Reverse primer with SmaI tail for cloning into pTRV2 vector
qRT-EpCH42_F	OL744077	AGACACAGGCTAAGGAAAGATC	Primers for qPCR amplification of the genes targeted by VIGS
qRT-EpCH42_R	OL744077	TCAACTAAAAACCTCATAGAATTTCTC	
qRT-EpCAS_F	KC702397	GAGAATAGTAAATCTTGTTCGTCTTAC	
qRT-EpCAS_R	KC702397	TTAGAGGGGAATTGGATCAACA	
qRT-CYP71D365_F	MW594405	ACGAAGCAGATTTACATGATCTC	
qRT-CYP71D365_R	MW594405	CTCTTGGAACTAACAATGGAAC	
qRT-EpSDR-5_F	MW594431	TCAACGTTTCTTCTGGTGC	
qRT-EpSDR-5_R	MW594431	CAATTTTGTCCTCGGAAAGATTG	
qRT-EpEF1a_F	OL744076	CTGGCATAATTAAGATGATTCCG	Primers for qPCR amplification of housekeeping genes
qRT-EpEF1a_R	OL744076	CTTCTTCTTGGCAGCAGATT	
qRT-EpSUMO_F	OL744078	CCAGGACCTGCTCGATC	
qRT-EpSUMO_R	OL744078	GATATAATCGTTTTCACATCAGACC	
qRT-EpSDR-2_F	MW594429	GTATCAACGCTAGGTGACC	Primers for qPCR amplification of the genes closely related to *EpSDR-5* and *CYP71D365* sequences targeted by VIGS.
qRT-EpSDR-2_R	MW594429	GTCCATAAGCACGAAATATGG	
qRT-EpSDR-4_F	MW594430	GGACACATTTTTGCATATCGC	
qRT-EpSDR-4_R	MW594430	GGAATACTGCTATATGCTGATTG	
qRT-EpSDR-6_F	MW594432	AATAATGCAGGTGTTACAGGAG	
qRT_EpSDR-6_R	MW594432	TTAATTAATGTTGTTTACCTTACAAGGA	
qRT-EpSDR-7_F	MW594433	CAATTGCAGGCAAATTGGAG	
qRT-EpSDR-7_R	MW594433	AGATACTGATTCAGAACTTCATCG	
qRT-EpSDR-8_F	MW594434	GGCTCACCGTTATACATATAGTG	

qRT-EpSDR-8_R	MW594434	AAGAATGCTTTTATACCCTGACTC	
qRT-CYP71D360_R	MW594404	CTATCGACAAATCTCTCTGGC	
qRT-CYP71D360_F	MW594404	TCCTCCTGTTCCGTTATTGA	
qRT-CYP71D367_R	MW594406	CTATCGACAAATCTCTCTGGC	
qRT-CYP71D367_F	MW594406	TCCTCCTGTTCCGTTATTGA	
qRT-CYP71D369_F	MW594407	TGTAATCAAGTGAACTACCTTGG	
qRT-CYP71D369_R	MW594407	GGGTTATGACATACCGGC	
qRT-CYP71D625_F	MW594408	CTCCTGGCCCTTTTTTGG	
qRT-CYP71D625_R	MW594408	CCCATCCATTGATAATGACTTTC	
qRT-CYP71D626_R	MW594409	GCACGAATTAAGCTATTTTTCTTGT	
qRT-CYP71D626_F	MW594409	CGACATTCTCTTGGAATTAACAG	
qRT-CYP71D627_F	MW594410	CTTTTCCCCTTGCATTATATTTCA	
qRT-CYP71D627_R	MW594410	TGTTGATACTTCAGCTACAACAG	

List of *S. cerevisiae* (wt) and codon optimized (opt) sequences

>tHMG1_wt
ATGGACCAATTGGTGAAAACTGAAGTCACCAAGAAGTCTTTTACTGCTCCTGTACAAAAGGC
TTCTACACCAGTTTTAACCAATAAAACAGTCATTTCTGGATCGAAAGTCAAAAGTTTATCATCT
GCGCAATCGAGCTCATCAGGACCTTCATCATCTAGTGAGGAAGATGATTCCCGCGATATTGA
AAGCTTGGATAAGAAAATACGTCCTTTAGAAGAATTAGAAGCATTATTAAGTAGTGGAAATAC
AAAACAATTGAAGAACAAAGAGGTCGCTGCCTTGGTTATTCACGGTAAGTTACCTTTGTACG
CTTTGGAGAAAAAATTAGGTGATACTACGAGAGCGGTTGCGGTACGTAGGAAGGCTCTTTCA
ATTTTGGCAGAAGCTCCTGTATTAGCATCTGATCGTTTACCATATAAAAATTATGACTACGAC
CGCGTATTTGGCGCTTGTTGTGAAAATGTTATAGGTTACATGCCTTTGCCCGTTGGTGTTATA
GGCCCCTTGGTTATCGATGGTACATCTTATCATATACCAATGGCAACTACAGAGGGTTGTTT
GGTAGCTTCTGCCATGCGTGGCTGTAAGGCAATCAATGCTGGCGGTGGTGCAACAACTGTT
TTAACTAAGGATGGTATGACAAGAGGCCCAGTAGTCCGTTTCCCAACTTTGAAAAGATCTGG
TGCCTGTAAGATATGGTTAGACTCAGAAGAGGGACAAAACGCAATTAAAAAAGCTTTTAACTC
TACATCAAGATTTGCACGTCTGCAACATATTCAAACTTGTCTAGCAGGAGATTTACTCTTCAT
GAGATTTAGAACAACTACTGGTGACGCAATGGGTATGAATATGATTTCTAAAGGTGTCGAATA
CTCATTAAAGCAAATGGTAGAAGAGTATGGCTGGGAAGATATGGAGGTTGTCTCCGTTTCTG
GTAACTACTGTACCGACAAAAAACCAGCTGCCATCAACTGGATCGAAGGTCGTGGTAAGAGT
GTCGTCGCAGAAGCTACTATTCCTGGTGATGTTGTCAGAAAAGTGTTAAAAAGTGATGTTTCC
GCATTGGTTGAGTTGAACATTGCTAAGAATTTGGTTGGATCTGCAATGGCTGGGTCTGTTGG
TGGATTTAACGCACATGCAGCTAATTTAGTGACAGCTGTTTTCTTGGCATTAGGACAAGATCC
TGCACAAAATGTTGAAAGTTCCAACTGTATAACATTGATGAAAGAAGTGGACGGTGATTTGAG
AATTTCCGTATCCATGCCATCCATCGAAGTAGGTACCATCGGTGGTGGTACTGTTCTAGAAC
CACAAGGTGCCATGTTGGACTTATTAGGTGTAAGAGGCCCGCATGCTACCGCTCCTGGTAC
CAACGCACGTCAATTAGCAAGAATAGTTGCCTGTGCCGTCTTGGCAGGTGAATTATCCTTAT
GTGCTGCCCTAGCAGCCGGCCATTTGGTTCAAAGTCATATGACCCACAACAGGAAACCTGCT
GAACCAACAAAACCTAACAATTTGGACGCCACTGATATAAATCGTTTGAAAGATGGGTCCGT
CACCTGCATTAAATCCTAA

>ALD6_wt
ATGACTAAGCTACACTTTGACACTGCTGAACCAGTCAAGATCACACTTCCAAATGGTTTGACA
TACGAGCAACCAACCGGTCTATTCATTAACAACAAGTTTATGAAAGCTCAAGACGGTAAGAC
CTATCCCGTCGAAGATCCTTCCACTGAAAACACCGTTTGTGAGGTCTCTTCTGCCACCACTG
AAGATGTTGAATATGCTATCGAATGTGCCGACCGTGCTTTCCACGACACTGAATGGGCTACC
CAAGACCCAAGAGAAAGAGGCCGTCTACTAAGTAAGTTGGCTGACGAATTGGAAAGCCAAAT
TGACTTGGTTTCTTCCATTGAAGCTTTGGACAATGGTAAAACTTTGGCCTTAGCCCGTGGGG
ATGTTACCATTGCAATCAACTGTCTAAGAGATGCTGCTGCCTATGCCGACAAAGTCAACGGT
AGAACAATCAACACCGGTGACGGCTACATGAACTTCACCACCTTAGAGCCAATCGGTGTCTG
TGGTCAAATTATTCCATGGAACTTTCCAATAATGATGTTGGCTTGGAAGATCGCCCCAGCATT
GGCCATGGGTAACGTCTGTATCTTGAAACCCGCTGCTGTCACACCTTTAAATGCCCTATACT

TTGCTTCTTTATGTAAGAAGGTTGGTATTCCAGCTGGTGTCGTCAACATCGTTCCAGGTCCTG
GTAGAACTGTTGGTGCTGCTTTGACCAACGACCCAAGAATCAGAAAGCTGGCTTTTACCGGT
TCTACAGAAGTCGGTAAGAGTGTTGCTGTCGACTCTTCTGAATCTAACTTGAAGAAAATCACT
TTGGAACTAGGTGGTAAGTCCGCCCATTTGGTCTTTGACGATGCTAACATTAAGAAGACTTTA
CCAAATCTAGTAAACGGTATTTTCAAGAACGCTGGTCAAATTTGTTCCTCTGGTTCTAGAATT
TACGTTCAAGAAGGTATTTACGACGAACTATTGGCTGCTTTCAAGGCTTACTTGGAAACCGAA
ATCAAAGTTGGTAATCCATTTGACAAGGCTAACTTCCAAGGTGCTATCACTAACCGTCAACAA
TTCGACACAATTATGAACTACATCGATATCGGTAAGAAAGAAGGCGCCAAGATCTTAACTGG
TGGCGAAAAAGTTGGTGACAAGGGTTACTTCATCAGACCAACCGTTTTCTACGATGTTAATG
AAGACATGAGAATTGTTAAGGAAGAAATTTTTGGACCAGTTGTCACTGTCGCAAAGTTCAAGA
CTTTAGAAGAAGGTGTCGAAATGGCTAACAGCTCTGAATTCGGTCTAGGTTCTGGTATCGAA
ACAGAATCTTTGAGCACAGGTTTGAAGGTGGCCAAGATGTTGAAGGCCGGTACCGTCTGGA
TCAACACATACAACGATTTTGACTCCAGAGTTCCATTCGGTGGTGTTAAGCAATCTGGTTACG
GTAGAGAAATGGGTGAAGAAGTCTACCATGCATACACTGAAGTAAAAGCTGTCAGAATTAAG
TTGTAA

> SEacs^{L641P}_opt
ATGAGCCAAACACATAAACACGCCATTCCCGCCAACATTGCGGATCGTTGCCTGATAAATCC
AGAGCAGTATGAGACTAAATATAAACAGTCTATTAACGACCCCGATACGTTTTGGGGCGAAC
AGGGAAAAATTCTCGATTGGATCACGCCGTACCAAAAAGTGAAAAACACCTCCTTTGCGCCA
GGCAATGTGTCGATTAAATGGTACGAGGACGGCACGCTGAATCTGGCGGCGAACTGTCTTG
ACCGCCATTTGCAGGAAAATGGCGATCGCACCGCCATTATCTGGGAAGGCGATGACGCGTC
GCAGAGTAAACATATCTCTTATCGCGAACTGCATCGCGATGTCTGCCGTTTCGCGAATACGC
TGTTGGATCTGGGCATTAAAAAAGGCGATGTGGTAGCGATTTATATGCCGATGGTACCGGAA
GCGGCGGTGGCAATGCTGGCCTGCGCCCGCATCGGCGCGGTGCATTCGGTGATCTTCGGG
GGCTTCTCGCCGGAAGCCGTCGCCGGACGCATTATCGACTCCAGCTCGCGGCTGGTGATC
ACCGCTGACGAAGGTGTACGTGCCGGACGCAGTATCCCGCTGAAAAAGAATGTCGATGACG
CGCTGAAAAACCCGAATGTCACTAGCGTTGAGCATGTGATCGTCCTGAAGCGCACCGGCAG
CGACATTGACTGGCAAGAAGGCCGCGACCTGTGGTGGCGCGATTTGATTGAAAAAGCCAGC
CCTGAGCACCAGCCTGAAGCGATGAATGCCGAAGATCCGCTGTTTATCCTTTATACCTCCGG
CTCCACCGGCAAGCCGAAAGGCGTGCTGCACACCACCGGCGGCTATCTGGTCTACGCCGC
GACCACCTTTAAGTATGTCTTTGATTATCACCCTGGCGATATTTACTGGTGTACCGCCGATGT
GGGTTGGGTGACGGGGCACAGCTATCTGTTGTATGGCCCGCTGGCCTGCGGCGCGACCAC
CTTAATGTTTGAAGGCGTGCCGAATTGGCCAACGCCCGCTCGCATGTGCCAGGTGGTCGAC
AAACACCAGGTCAACATTCTCTATACCGCCCCGACGGCCATCCGCGCGCTGATGGCGGAAG
GCGATAAAGCCATTGAAGGCACCGACCGTTCTTCACTGCGCATTCTGGGTTCCGTCGGCGA
GCCGATCAATCCCGAAGCGTGGGAATGGTACTGGAAGAAGATCGGCAATGAAAAATGTCCG
GTCGTCGACACCTGGTGGCAGACTGAAACAGGCGGTTTTATGATCACGCCGCTACCGGGCG
CTATCGAACTGAAAGCCGGTTCCGCCACCCGTCCTTTCTTTGGCGTACAGCCTGCGCTGGT
GGATAACGAAGGCCATCCGCAAGAAGGCGCGACGGAAGGCAATCTGGTCATCACCGATTCC
TGGCCGGGCCAGGCGCGTACTCTGTTCGGCGATCATGAACGTTTTGAGCAGACCTATTTCT
CTACCTTTAAGAATATGTATTTCAGCGGCGACGGCGCGCGTCGCGATGAAGACGGCTATTAC
TGGATCACCGGTCGCGTGGATGACGTGTTAAACGTCTCCGGCCACCGTCTGGGTACGGCG
GAAATCGAGTCAGCGCTGGTGGCGCATCCGAAGATCGCCGAAGCGGCGGTGGTGGGTATT
CCACACGCTATCAAAGGCCAGGCGATTTACGCTTATGTGACGCTCAACCACGGCGAGGAGC
CGTCGCCAGAACTGTACGCGGAGGTGCGCAACTGGGTACGTAAAGAGATTGGCCCGCTGG
CGACGCCGGACGTGCTGCACTGGACCGACTCACTACCAAAAACCCGTTCCGGCAAAATTAT
GCGCCGCATTTTGCGCAAAATCGCGGCGGGCGATACCAGCAATCTGGGCGATACCTCGACT
CTCGCCGATCCTGGCGTGGTGGAGAAACCGCTCGAAGAGAAGCAGGCCATCGCGATGCCG
TCATAA

>*MtGGPPS*_opt
ATGACTGAAGTTTTGGACATCTTGAGAAAGTACTCTGAAATGGCTGACGAAAGAATCAGAGA
ATCTATCTCTGACATCACTCCAGAAACTTTGTTGAGAGCTTCTGAACACTTGATCACTGCTGG
TGGTAAGAAGATCAGACCATCTTTGGCTTTGTTGTCTTCTGAAGCTGTTGGTGGTGACCCAG
GTGACGCTGCTGGTGTTGCTGCTGCTATCGAATTGATCCACACTTTCTCTTTGATCCACGAC
GACATCATGGACGACGACGAAATCAGAAGAGGTGAACCAGCTGTTCACGTTTTGTGGGGTG

AACCAATGGCTATCTTGGCTGGTGACGTTTTGTTCTCTAAGGCTTTCGAAGCTGTTATCAGAA
ACGGTGACTCTGAAATGGTTAAGGAAGCTTTGGCTGTTGTTGTTGACTCTTGTGTTAAGATCT
GTGAAGGTCAAGCTTTGGACATGGGTTTCGAAGAAAGATTGGACGTTACTGAAGAAGAATAC
ATGGAAATGATCTACAAGAAGACTGCTGCTTTGATCGCTGCTGCTACTAAGGCTGGTGCTAT
CATGGGTGGTGGTTCTCCACAAGAAATCGCTGCTTTGGAAGACTACGGTAGATGTATCGGTT
TGGCTTTCCAAATCCACGACGACTACTTGGACGTTGTTTCTGACGAAGAATCTTTGGGTAAG
CCAGTTGGTTCTGACATCGCTGAAGGTAAGATGACTTTGATGGTTGTTAAGGCTTTGGAAAG
AGCTTCTGAAAAGGACAGAGAAAGATTGATCTCTATCTTGGGTTCTGGTGACGAAAAGTTGG
TTGCTGAAGCTATCGAAATCTTCGAAAGATACGGTGCTACTGAATACGCTCACGCTGTTGCT
TTGGACCACGTTAGAATGGCTAAGGAAAGATTGGAAGTTTTGGAAGAATCTGACGCTAGAGA
AGCTTTGGCTATGATCGCTGACTTCGTTTTGGAAAGAGAACACTAA

> *nMBP-JcCAS-erg20F96c*_opt
ATGAAGATCGAAGAAGGCAAGTTGGTTATTTGGATCAATGGTGATAAGGGTTACAACGGTTT
GGCTGAAGTTGGTAAGAAGTTCGAAAAAGACACCGGTATTAAGGTTACCGTTGAACATCCAG
ATAAGCTGGAAGAAAAGTTTCCACAAGTTGCTGCTACTGGTGATGGTCCAGATATTATCTTTT
GGGCTCATGATAGATTTGGTGGTTATGCTCAATCTGGTTTGTTGGCTGAAATTACACCAGATA
AGGCTTTCCAAGATAAGTTGTATCCATTCACTTGGGATGCCGTTAGATACAACGGTAAATTGA
TTGCTTACCCAATTGCTGTTGAAGCCTTGTCTTTGATCTACAACAAAGACTTGTTGCCCAATC
CACCAAAAACCTGGGAAGAAATTCCAGCTTTGGACAAAGAATTGAAGGCCAAAGGTAAATCC
GCCTTGATGTTTAACTTGCAAGAACCATATTTCACGTGGCCATTGATTGCAGCTGATGGTGG
TTACGCTTTTAAGTACGAAAATGGCAAGTACGATATCAAGGATGTTGGTGTTGATAATGCTGG
TGCTAAAGCTGGTTTGACTTTCTTGGTTGACCTGATTAAGAACAAGCACATGAACGCTGATAC
CGATTACTCTATTGCTGAAGCTGCTTTTAACAAGGGTGAAACTGCTATGACTATTAACGGTCC
ATGGGCTTGGTCTAACATTGATACTTCTAAGGTTAACTACGGTGTTACCGTTTTGCCAACTTT
TAAAGGTCAACCTTCTAAGCCATTCGTTGGTGTTTTGTCTGCTGGTATTAACGCTGCTTCTCC
AAACAAAGAACTGGCTAAAGAATTCCTGGAAAACTACTTGTTGACCGACGAAGGTTTGGAAG
CTGTTAACAAAGATAAGCCATTGGGTGCTGTTGCTTTGAAGTCTTATGAAGAAGAATTGGCCA
AGGATCCAAGAATTGCTGCAACTATGGAAAATGCTCAAAAGGGTGAGATTATGCCAAACATC
CCACAAATGTCTGCTTTTTGGTATGCTGTTAGAACCGCTGTTATCAATGCTGCTTCTGGTAGA
CAAACTGTTGATGAAGCTTTGAAGGATGCCCAAACCAGAATTACTAAGGGTTTAGAAGTTTTG
TTCCAAGGTCCAGCTATGGCTTCTACAAAATCTGAAACTGAAGCTAGACCATTGGCTTACTTT
CCACCAACTGTTTGGGGTGATAGATTGGCTTCTTTGACATTCAATCAACCAGCCTTTGAGTTG
TTGTCTAAGCAAGTCGAATTGCTGAACGAGAAGATCAAGAAAGAGATGTTGAACGTTTCCAC
CTCTGATTTGGCCGAAAAGATTATCTTGATCGACTCTTTGTGCAGATTGGGTGTTTCTTACCA
TTTCGAAGAAGAGATCCAAGAGAACTTGACCAGAATTTTCAATACCCAGCCAAACTTCTTGAA
CGAAAAGGATTACGATTTGTTCACCGTTGCCGTTATCTTCAGAGTTTTCAGACAACATGGTTT
CAAGATCAGCTCCGATGTTTTCAACAAGTTCAAGGATTCCGATGGCAAGTTCAAAGAGAGTT
TGTTGAACGACATTAAGGGCATCTTGTCCTTGTTTGAAGCTACCCATGTTTCTATGCCAAATG
AACCTATTTTGGACGAAGCTTTGGCTTTTACCAAGGCTTTTTTGGAATCTTCCGCTGTTAAGT
CTTTTCCAAATTTCGCCAAGCACATTTCCTCTGCTTTGGAACAACCAGTTCATAAGGGTATTC
CTAGATTGGAAGCCAGAAAGTACATTGACTTGTACGAAGTTGACGAGTCTAGAAACGAAACC
GTTTTGGAATTGGCAAAGTTGGATTTTAACAGGGTTCAGTTGTTGCACCAAGAAGAGTTGTCT
CAATTTTCTAAGTGGTGGAAGTCCTTGAATATCTCTGCTGAAGTTCCATACGCTAGAAACAGA
ATGGCCGAAATCTTTTTTTGGGCCGTTTCTATGTACTTCGAACCACAATACGCTAAGGCTAGA
ATGATTGTCTCTAAGGTTGTCCTGTTGATCTCCTTGATTGATGATACCATTGATGCTTACGCT
ACCATCGACGAAATTCATAGAGTTGCTGATGCTATCGAAAGATGGGATATGAGATTGGTTGA
TCAGTTGCCAAATTACATGAAGGTCATCTACAGGTTGATCATCAACACCTTCGATGAATTCGA
AAAGGACTTAGAAGCTGAGGGTAAATCCTACTCTGTTAAGTATGGTAGAGAAGCCTATCAAG
AATTGGTCAGAGGTTATTACTTGGAGGCTATTTGGAAAGCAGATGGTAAGGTTCCATCTTTTG
ATGAGTACATCTACAACGGTGGTGTTACTACTGGTTTGCCATTGGTTGCTACTGTTTCTTTTA
TGGGTGTCAAAGAGATCAAGGGTACTAAGGCTTTTCAATGGTTGAAAACGTACCCAAAGTTG
AATCAAGCTGGTGGTGAATTCATCAGATTGGTTAACGATGTTATGTCCCACGAAACCGAACA
AGATAGAGGTCATGTTGCTTCATGTATCGACTGCTATATGAAGCAATACGGTGTCTCTAAAGA
AGAAGCCGTTGAAGAAATCCAAAAAATGGCTACCAATGAGTGGAAGAAGTTGAACGAACAAT
TGATCGTCAGATCCACTGAAGTTGTTCCAGTCAATTTGTTGATGAGGATCGTTAACTTGGTTA
GGTTGACTGATGTCTCTTACAAATACGGTGATGGTTACACCGATTCCTCTCAGTTGAAAGAAT

ATGTCAAGGGTTTGTTCATTGAACCTATCGCTACTGGTTCTGGTTCAGGTTCTGGTAGTGGTT
CAATGGCTTCTGAAAAAGAAATCAGAAGGGAACGTTTCTTGAATGTGTTCCCTAAGTTGGTTG
AAGAGTTGAATGCTTCTTTGTTGGCATACGGAATGCCAAAAGAAGCTTGCGATTGGTACGCT
CATTCTTTGAATTACAATACTCCAGGTGGTAAGTTGAACAGAGGTTTGTCTGTTGTTGATACC
TACGCCATTTTGTCTAACAAGACTGTTGAACAATTGGGCCAAGAAGAATACGAAAAGGTTGC
TATTTTAGGTTGGTGCATCGAATTATTGCAAGCCTACTGTTTGGTTGCCGATGATATGATGGA
TAAGTCCATTACTAGAAGAGGTCAGCCATGTTGGTACAAAGTTCCAGAAGTTGGAGAAATTG
CTATCAACGATGCTTTCATGTTGGAAGCAGCTATCTACAAGTTGTTGAAGTCCCATTTTAGAA
ACGAGAAGTACTACATCGATATCACCGAGTTGTTTCACGAAGTTACCTTCCAAACTGAATTGG
GTCAATTGATGGATTTGATTACTGCCCCAGAAGATAAGGTTGACTTGTCCAAATTCTCCTTGA
AGAAGCACTCTTTCATCGTTACTTTCAAGACCGCTTACTACTCTTTCTATTTGCCAGTTGCTTT
GGCTATGTACGTTGCAGGTATTACTGATGAGAAGGATTTGAAGCAAGCCAGAGATGTTTTGA
TTCCTTTGGGTGAATACTTCCAAATCCAAGATGATTACCTGGATTGCTTTGGTACTCCAGAAC
AAATTGGTAAGATCGGTACTGATATCCAAGACAACAAATGCTCCTGGGTTATTAACAAGGCTT
TGGAATTAGCTTCCGCCGAACAAAGAAAAACCTTGGACGAAAATTACGGCAAGAAGGATTCT
GTTGCCGAAGCTAAATGTAAGAAGATTTTCAACGACCTGAAAATCGAGCAGTTGTACCATGA
ATACGAAGAATCCATTGCTAAGGACTTGAAGGCTAAGATCTCTCAAGTTGATGAATCCAGAG
GTTTCAAGGCTGATGTTTTAACTGCTTTCTTGAACAAGGTCTACAAGAGGTCTAAGTAG

>*CYP71D495*_opt
ATGTTGTTCTTCATCACTGTTTTGTTCATCTTCATCGCTTTGAGAATCTGGAAGAAGTCTAAG
GCTAACTCTACTCCAAACTTGCCACCAGGTCCAAACAAGTTGCCATTGATCGGTAACGTTCA
CAACTTGGTTGGTGACTTGCCATACCACAGATTGAGAGACTTGTCTAAGAAGTACGGTCCAA
TCATGCACTTGCAATTGGGTGAAAACACTACTGTTGTTATCTCTTCTCCAGAATTGGCTCAAG
AAGTTATGAAAACTCACGACGTTAACTTCGCTCAAAGACCATTCGTTTTGGCTGGTGACATCG
TTTCTTACAAGTGTAAGGACATCGCTTTCGCTCCATACGGTGAATACTGGAGACAATTGAGAA
AGATGTGTTCTTTGGAATTGTTGACTGCTAAGAGAGTTCAATCTTTCAAGTCTATCAGAGAAG
AAGAAGTTTCTAAGTTGGTTGAATCTATCTCTTCTTCTTCTGGTTCTCCAATCAACTTCTCTAA
GATGGCTTCTTCTTTGACTTACGCTATCATCTCTAGAGCTGTTTGTGGTAAGGTTTCTAGAGG
TGAAGAAGTTTTCGTTCCAGCTGTTGAAAAGTTGGTTGAAGCTGGTAGATCTATCTCTTTGGC
TGACTTGTACCCATCTGTTAAGTTGTTCAACGCTTTGTCTGTTGTTAGAAGAAGAGTTGAAAA
GATCCACGGTGAAGTTGACAAGATCATCGAAAACATCGTTATCGAACACAGAGAAAGAAAGA
GAATGGCTCACGCTGGTATCAACTCTAAGGAAGAAGAAGATTTGGTTGACGTTTTGTTGAAG
TTCCAAGAAAACGGTGACTTGGACTCTTACTTGTCTAACGACGGTATCAAGGCTGTTATCTTG
GACATGTTCATCGCTGGTTCTGACACTTCTTCTACTACTATCGAATGGGCTATCTCTGAAATG
GTTAAGAACCCATCTATCATGGAAAAGGCTCAAGCTGAAGTTAGAGAAGTTTTCGGTTCTAA
GGGTAAGGTTGACGAAGCTGACTTGCACGAATTGAACTACTTGAAGTTGGTTATCAAGGAAA
CTTTGAGATTGCACCCAGCTGTTCCATTGTTGTTGCCAAGACAATCTAGAGAAGATTGTGTTA
TCGAAGGTTACAACATCGCTACTAAGTCTACTGTTATCGTTAACGCTTGGGCTATCGCTAGA
GATCCAAAGTACTGGGACGAAGCTGAAAGATTCTACCCAGAAAGATTCATCAACTCTTCTATC
GACTTCAAGGGTACTAACTTCGAATTCATCCCATTCGGTGCTGGTAGAAGAATGTGTCCAGG
TATGTTGTTCGGTTTGGCTTCTGTTGAATTGCCATTGGCTCAATTGTTGTACCACTTCGACTG
GAAGTTGCCAGGTGGTCAAAAGCCAGAAGATTTGGACATGTCTGACGACTTGGACGGTACT
GCTACTAGGAGACACGCTTTGTACTTGACTGCTACTCCATACTTGCCATCTGCTGTTGGTAA
GATCTCTAGATAA

>*CYP726A20*_opt
ATGGAACACCAAATCTTGTCTTTCCCAGTTTTGTTCTCTTTGTTGTTGTTCATCTTGGTTTTGT
TGAAGGTTTCTAAGAAGTTGTACAAGCACGACTCTAAGCCACCACCAGGTCCATGGAAGTTG
CCATTCATCGGTAACTTGATCCAATTGGTTGGTGACACTCCACACAGAAGATTGACTGCTTTG
GCTAAGACTTACGGTCCAGTTATGGGTGTTCAATTGGGTCAAGTTCCATTCTTGGTTGTTTCT
TCTCCAGAAACTGCTAAGGAAGTTATGAAGATCCAAGACCCAGTTTTCGCTGAAAGACCATT
GGTTTTGGCTGGTGAAATCGTTTTGTACAACAGAAACGACATCGTTTTCGGTTCTTACGGTGA
CCAATGGAGACAAATGAGAAAGTTCTGTACTTTGGAATTGTTGTCTACTAAGAGAGTTCAATC
TTTCAGACCAGTTAGAGAAGAAGAAGTTGCGAGCTTCGTTAAGTTGATGAGAACTAAGAAGG
GTACTCCAGTTAACTTGACTCACGCTTTGTTCGCTTTGACTAACTCTATCGTTGCTAGAAACG
CTGTTGGTCACAAGTCTAAGAACCAAGAAGCTTTGTTGGAAGTTATCGACGACATAGTTGTAA

GCGGTGGTGGTGTTTCTATCGTTGACATCTTCCCATCTTTGCAATGGTTGCCAACTGCTAAG
AGAGAAAGATCTAGAATCTGGAAGTTGCACCAAAACACTGACGAAATCTTGGAGGACATCTT
GCAAGAACACAGAGCTAAGAGACAAGCTACTGCTTCTAAGAACTGGGACAGATCTGAAGCT
GACAACCTCCTCGACGTGCTATTGGACTTGCAACAATCTGGTAACTTGGACGTTCCATTGAC
TGACGTTGCTATCAAGGCTGCTATCATCGACATGTTCGGTGCTGGTTCTGACACTTCTTCTAA
GACTGCTGAATGGGCTATGGCTGAATTGATGAGAAACCCAGAAGTTATGAAGAAGGCTCAAG
AAGAATTGAGAAACTTCTTCGGTGAAAACGGTAAGGTTGAAGAAGCTAAGTTGCACGAATTG
AAGTGGATCAAGTTGATCATCAAGGAAACTTTGAGATTGCACCCAGCTGTTGCTGTTATCCC
AAGAGTTTGTAGAGAAAAGACTAAGGTTTACGGTTACGACGTTGAACCAGGTACTAGAGTTT
TCATCAACGTTTGGTCTATCGGTAGAGATCCAAAGGTTTGGTCTGAAGCTGAAAGATTCAAG
CCAGAAAGATTCATCGACTCTGCTATCGACTACAGAGGTTTGAACTTCGAATTGATCCCATTC
GGTGCTGGTAAGAGAATCTGTCCAGGTATGACTTTGGGTATGGCTAACTTGGAAATCTTCTT
GGCTAACTTGTTGTACCACTTCGACTGGAAGTTCCCAAAGGGTGTTACTGCTGAAAACTTGG
ACATGAACGAAGCTTTCGGTGGTGCTGTTAAGAGAAAGGTTGACTTGGAATTGATCCCAATC
CCATTCAGACCATAA

>*JcCPR*_opt
ATGTCATCTGACTTGGTTAGATACGTTGAATCTGTTTTGGGTGTTTCTTTGGGTGGTTCTGTT
ACTGACTCTTTGTTGTTGATCGTTACTACTTCTGTTGCTGTTATCGTTGGTTTGTTCGTTTTCT
TGTTGAAGAAATCTTCTGACAGATCTAAGGAAGTTAAGCCAGTTGTTGTTCCAAAGTCTTTGA
CTGTTAAGAAGGAAGAAGATGACGCTGAAGCTTTGGCTGGTAAGACTAGAGTTATCATCTTC
TACGGTACTCAAACTGGTACTGCGGAAGGCTTCGCTAAGTCTCTCTCTGAAGAAATCAAGGC
TAGATACGAAAAGGCTGCTGTTAAGGTTGTTGACTTGGACGACTACGCTGCTGACGACGAAG
AATACGAAGAAAAGTTGAAGAAGGAAACTTTGTCTTTCTTCATGGTTGCTACTTACGGTGACG
GTGAACCAACTGACAACGCTGCTAGATTCTACAAGTGGTTGACTGAAGAAAACGAAAGAGGT
GTTTGGTTGCAACAATTGACTTTCGGTGTTTTCGGTTTGGGTAACAGACAATACGAACACTTC
AACAAGATCGCTAAGGTTTTGGACGAACAATTGTCTGAACAAGGTGCTAAGAGATTGATCCC
AGTTGGTTTGGGTGACGACGACCAATGTATCGAAGATGACTTCTCTGCTTGGAAGGAATTGT
TGTGGCCAGAATTGGACCAAATCTTGAGAGACGAAGATGACGTTAACACTCCATCTACTCCA
TACACTGCTGCTATCTTGGAATACAGAGTTGTTATCCACGACGCTTCTATGACTTCTTTCGAC
GACAAATCTTCTCACTTGGCTAACGGCAATACTGTATTCGACATCCACCATCCATGTAGAGCT
AACGTTGCTGTTCAAAAGGAATTGCACAAGCCAGAATCTGACAGATCTTGTATCCACTTGGA
ATTCGACGTTGCTGGTACTGGTATCACTTACGAAACTGGTGACCACGTTGGTGTTTACTCTG
AAAACTTCGACGAAACTGTTGAAGAAGCTGCTAAGTTGTTGGGTCAACCATTGGACTTGTTG
TTCTCTGTTTACACTGACAACGAAGATGGTACTCCATTGGGTTCTTCTTTGCCACCAGCTTTC
CCAGGTCCATGTACTTTGAGAACTGCTTTGGCTAGATACGCTGACTTGTTGAACTCTCCAAG
AAAGGCTGCTTTGATCGCTTTGGCTGCTCACGCTTCTGACCCATCTGAAGCTGAAAGATTGA
GATTCTTGGCTTCTCCACAAGGTAAGGACGAATACGCTCAATGGATCGTTGCTTCTCAGCGT
AGCTTGTTGGAAGTTATGGCTGAATTCCCATCTGCTAAGCCCCCACTCGGTGTATTCTTCGC
AGCTGTTGCTCCAAGATTGCAACCAAGATACTACTCTATCTCTTCTTCTCCAAGATTCGCTCC
ATCTAGAGTTCACGTTACTTGTGCTTTGGTTTACGGTCCAACTCCAACTGGTAGAATCCACAA
GGGTGTTTGTTCTACTTGGATGAAGAACGCTGTTCCATTGGAAAGATCTTCTGACTGTTCTTG
GGCTCCAATCTTCATCAGAACTTCTAACTTCAAGTTGCCATCTGACCCATCTGTTCCAATCAT
CATGGTTGGTCCGGGTACAGGTTTGGCTCCATTTAGGGGCTTCTTGCAAGAACGTATGGCTT
TGAAGCAAGAAGGTGCTCAATTGGGTCCAGCTTTGTTGTTCTTCGGTTGTAGAAACAGAAGA
ATGGACTTCATCTACGAAGATGAATTGAACAACTTCGTTGAACAAGGTGTTATCTCTGAATTG
ATCGTTGCTTTCTCTAGAGAAGGTCCACAAAAGGAATACGTTCAACACAAGATGGTTCAAAA
GGCTGCTCAAATCTGGGCTATCATCTCTCAAGGTGGTTACTTGTACGTTTGTGGTGACGCTA
AGGGTATGGCTAGAGACGTTCATCGTACTCTCCACAACATCGTTCAAGAACAAGGTAACTTG
GACGCTTCTAAGACTGAATCTATGGTTAAGAAGTTGCAAATGGACGGTAGATACTTGAGAGA
CGTTTGGTAA

>*JcADH1*_opt
ATGGCTTCCTCCAGCAGCCCAGCGCCCACCGCAAAGAGATTGGAAGGGAAGGTCGCACTG
ATTACTGGTGGCGCATCCGGTATAGGAGAGTGCACCGCACGTCTTTTCGCTCGTCATGGGG
CCAAGGTAATCATTGCCGATGTGCAGTCAGAGTTGGGTAGGTCTGTGGCTGAAAAGATAGG
GAGTGAGACGGGTCAGCCAGTAACATATGTAGACTGTAATGTCACCGTAGAATCAGACGTC

GAAAACGCTGTGAATACAGCGGTAAGTCTGCATGGGAAGTTGGACATTATGTTCAATAATGC
CGGCATTGCCGGGAACAACCATGACAAAATCCTTTCTACTGAGCGTGAGGACTTCATGAGAG
TGCTTGATATAAATATATATGGAGGAGTGTTAGGGGCAAAGCACGCCGCAAGAGTCATGATC
CCAGAAAAAAAAGGCTGCATTTTGTTCACGGCTTCTGTCAGTAGCGCGTTGTACGGAGGTCC
CTATGCCTATACGGCCTCAAAACATGCTGTGGTCGGATTAGCGAAAAATTTAGCAATCGAATT
GGGCCAGCATGGGATTAGAGTAAACTGTATTTCTCCCGGAGCGGTACAGACAGGGCTAGCG
AAACAGTTGGGCCTTTCTGAGCAACAAGTGCAGGAGTGGTCCAGTGCGTTGGCTAACTTGA
AGGTTGTGAAGTTAGAGGTGAACGACATAGCGGAAGCAGCCTTGTACCTGGCCTCTGATGA
TTCAAAATTTGTATCCGGGCTTAATCTGCTAGTCGATGGAGCTGCGAGTTTACCCACTACTAC
TCGTGCGTATTAG

> *EpSDR-1*_opt
ATGATGATTTTCGGGCGTAAGGGCCCCTCTGGCTTCTCAACAAAGAGTACGGCGGAGGAGG
TCACGCAAGGAATAGACGGTTCCAGTTTGACAGCGATTATCACCGGGGCATCTAGCGGGAT
TGGCGCTGAAACTGCTCGTGTGTTGGCTATGAGAGGTGTTCATGTAGTTATGCCTGTTCGTA
ACGTTCAGACGGGCACGAAAGTAAAAGAAACGATCCTGAAGCAGAACCCGTCTGCTAAGGT
CGAGGTAATGCATCTTGATCTTTCTAGTATGACGAGTATTAGAAAGTTCGCCTCCGAATACAT
TAGCAGCGGGAGACCTTTGAACATCTTAATCAATAACGCCGCAATAGCTATGATTCCGTTCA
CATTATCCGAAGATGACATCGAACTGCAATTCGCCACTAATCACGTAGGACACTTTCTGTTGA
CAGAGTTGCTATTAGAGACCATGAAGAACACAACCCGTGAGAGTAATCAGGAGGGCAGAAT
AGTGAACGTTTCCTCAGAAGGGCACAACATCGCGTACCCCGGTGGGATAAGGTTTGGGCAA
ATTAATGACCAATCTGGATATCGTAAAGACCTGGGCTACTCCCAAAGTAAGTTGGCGAATAT
CTTGCACGCGAACGAGTTGGCCCGTCGTCTAAAGGAGGACGGAGTAGATATTACAGCGAAT
AGCCTACATCCGGGTGGAATTGTAACTAACATAGTTCGTCACCACAAAATCATATATGGAATA
ACGTTCTTATTGGACAAGTTTCTGCTAAAGAACTGTCAACAGGGCGCGGCGACAACATGCTA
CGTGGCTTTGCACCCGCAAGTAAAAGGAATAAGCGGACAGTATTTCGTGGACTCAAACTTGG
CCAAGCCGAGTTCTCTTGCGACAGATGCGGAATTGGGAAAGAGATTATGGGATTTCTCCCTA
AAGTTGATTAACTCCAACTAG

>*EpSDR-5*_opt
ATGGCAATAGAAAAGAATTGTCGTTATGCTGTCGTTTCAGGTGGTAATAGGGGTATTGGGTA
TGGAGTCTGCAAGCAATTGGCCAGTAACGGAATCACGGTAATCCTTACGGCACGTGATGAAA
AGAGGGGGTTAGAGGCGGTCGAGAAGTTAAAAGATGACTCAGGTCTGAGCAATGTCTTATT
CCATCAACTTGATGTCACCGATCCCGCCTCTATAGATCTACTTGCGCAATTTATAAAAAGCCA
CTTCGGCAAGCTAGATATACTTGTAAATAATGCAGGGGTAACCGGAGTCAAGGTTGATTATG
ATGCACCAACTCAAGTGGACCCTTGCAAAGAAGTTGATCCAGGAGTCATGTGGTATGATACA
CTGTCCGAAAGATATGAGGTTCCCGAAGAGTGTTTTAGTATAAATTACTACGGTGCAAAGTC
CATGATCGAGAAACTAATACCATTGCTTCAGTTGTCTGATGCACCGAGAATCGTGAATGTCA
GTTCAGGGGCGGGGAAGATGCAATACCTTCTTCACGAATGGGCCATCAAAGTGCTGTCCGA
TGCCGACAACTTGAGCGAGGACAAAATAGATGAATTAGTCAACCAGTACCTGAAAGACTTCA
AACAAGGGTCTTTAGAGTCAAAGGGTTGGCCGGCTGACTTGTCTGGCTATAAGCTGTCTAAG
GCCACAATAAATGCATACACGCGTATCATTGCCAAGAAGTTTCCTACCTTTCGTATTAATTGT
TTGTGTCCAGGATACGTCAAGACTGACATTAATCTGAATACAGGGATTCTTAGCGTGGATGA
GGGAGCGGAATCTGTTGTTCGTTTGGCGTTGCTTCCCGATGATGGTCCTTCTGGCTCCTTTT
TCGATCAAAAAACTGAAAGTACCTTTTGA

Supplemental References

1. A. J. King *et al.*, A Cytochrome P450-Mediated Intramolecular Carbon-Carbon Ring Closure in the Biosynthesis of Multidrug-Resistance-Reversing Lathyrane Diterpenoids. *Chembiochem* **17**, 1593-1597 (2016).
2. J. L. Giner, J. D. Berkowitz, T. Andersson, Nonpolar components of the latex of Euphorbia peplus. *J Nat Prod* **63**, 267-269 (2000).
3. B. J. Haas *et al.*, De novo transcript sequence reconstruction from RNA-seq using the Trinity platform for reference generation and analysis. *Nat Protoc* **8**, 1494-1512 (2013).

4. H. Li, R. Durbin, Fast and accurate short read alignment with Burrows-Wheeler transform. *Bioinformatics* **25**, 1754-1760 (2009).
5. H. Li *et al.*, The Sequence Alignment/Map format and SAMtools. *Bioinformatics* **25**, 2078-2079 (2009).
6. M. D. Robinson, D. J. McCarthy, G. K. Smyth, edgeR: a Bioconductor package for differential expression analysis of digital gene expression data. *Bioinformatics* **26**, 139-140 (2010).
7. A. J. King, G. D. Brown, A. D. Gilday, T. R. Larson, I. A. Graham, Production of bioactive diterpenoids in the euphorbiaceae depends on evolutionarily conserved gene clusters. *Plant Cell* **26**, 3286-3298 (2014).
8. N. J. Loman, J. Quick, J. T. Simpson, A complete bacterial genome assembled de novo using only nanopore sequencing data. *Nat Methods* **12**, 733-U751 (2015).
9. F. Sainsbury, E. C. Thuenemann, G. P. Lomonossoff, pEAQ: versatile expression vectors for easy and quick transient expression of heterologous proteins in plants. *Plant Biotechnol J* **7**, 682-693 (2009).
10. R. Hofgen, L. Willmitzer, Storage of competent cells for Agrobacterium transformation. *Nucleic Acids Res* **16**, 9877 (1988).
11. E. C. F. Forestier *et al.*, Developing a Nicotiana benthamiana transgenic platform for high-value diterpene production and candidate gene evaluation. *Plant Biotechnol J* **19**, 1614-1623 (2021).
12. F. A. Trikka et al., Iterative carotenogenic screens identify combinations of yeast gene deletions that enhance sclareol production. Microb Cell Fact 14, 60 (2015).
13. Y. Shiba, E. M. Paradise, J. Kirby, D. K. Ro, J. D. Keasling, Engineering of the pyruvate dehydrogenase bypass in Saccharomyces cerevisiae for high-level production of isoprenoids. Metab Eng 9, 160-168 (2007).
14. J. Wong et al., High-titer production of lathyrane diterpenoids from sugar by engineered Saccharomyces cerevisiae. Metab Eng 45, 142-148 (2018).
15. F. Farzadfard, S. D. Perli, T. K. Lu, Tunable and multifunctional eukaryotic transcription factors based on CRISPR/Cas. ACS Synth Biol 2, 604-613 (2013).
16. J. E. DiCarlo et al., Genome engineering in Saccharomyces cerevisiae using CRISPR-Cas systems. Nucleic Acids Res 41, 4336-4343 (2013).
17. R. D. Gietz, R. H. Schiestl, Quick and easy yeast transformation using the LiAc/SS carrier DNA/PEG method. Nat Protoc 2, 35-37 (2007).
18. F. Ratcliff, A. M. Martin-Hernandez, D. C. Baulcombe, Technical Advance. Tobacco rattle virus as a vector for analysis of gene function by silencing. *Plant J* **25**, 237-245 (2001).
19. K. Khan, V. Kumar, A. Niranjan, A. Shanware, V. A. Sane, JcMYB1, a Jatropha R2R3MYB Transcription Factor Gene, Modulates Lipid Biosynthesis in Transgenic Plants. *Plant Cell Physiol* **60**, 462-475 (2019).
20. Gasic, K., Hernandez, A., and Korban, S.S. (2004). RNA extraction from different apple tissues rich in polyphenols and polysaccharides for cDNA library construction. Plant Mol. Biol. Rep. 22: 437a–437g.
21. J. M. Ruijter *et al.*, Amplification efficiency: linking baseline and bias in the analysis of quantitative PCR data. *Nucleic Acids Res* **37**, e45 (2009).
22. L. Zhang, L. L. He, Q. T. Fu, Z. F. Xu, Selection of reliable reference genes for gene expression studies in the biofuel plant Jatropha curcas using real-time quantitative PCR. *Int J Mol Sci* **14**, 24338-24354 (2013).
23. H. Moummou, Y. Kallberg, L. B. Tonfack, B. Persson, B. van der Rest, The Plant Short-Chain Dehydrogenase (SDR) superfamily: genome-wide inventory and diversification patterns. Bmc Plant Biol 12 (2012).
24. R. C. Edgar, MUSCLE: multiple sequence alignment with high accuracy and high throughput. Nucleic Acids Res 32, 1792-1797 (2004).
25. J. Castresana, Selection of conserved blocks from multiple alignments for their use in phylogenetic analysis. Mol Biol Evol 17, 540-552 (2000).
26. A. Stamatakis, RAxML-VI-HPC: maximum likelihood-based phylogenetic analyses with thousands of taxa and mixed models. Bioinformatics 22, 2688-2690 (2006).
